# Genome-Protecting Compounds as Potential Geroprotectors

**DOI:** 10.3390/ijms21124484

**Published:** 2020-06-24

**Authors:** Ekaterina Proshkina, Mikhail Shaposhnikov, Alexey Moskalev

**Affiliations:** 1 Laboratory of Geroprotective and Radioprotective Technologies, Institute of Biology, Komi Science Centre, Ural Branch, Russian Academy of Sciences, 28 Kommunisticheskaya st., 167982 Syktyvkar, Russia; kateplus@mail.ru (E.P.); mshaposhnikov@mail.ru (M.S.); 2 Pitirim Sorokin Syktyvkar State University, 55 Oktyabrsky prosp., 167001 Syktyvkar, Russia; 3 Center for Precision Genome Editing and Genetic Technologies for Biomedicine, Engelhardt Institute of Molecular Biology, Russian Academy of Sciences, 119991 Moscow, Russia

**Keywords:** geroprotectors, genomic protection, antioxidants, epidrugs, DNA repair activators, senolytics, senomorphics, aging

## Abstract

Throughout life, organisms are exposed to various exogenous and endogenous factors that cause DNA damages and somatic mutations provoking genomic instability. At a young age, compensatory mechanisms of genome protection are activated to prevent phenotypic and functional changes. However, the increasing stress and age-related deterioration in the functioning of these mechanisms result in damage accumulation, overcoming the functional threshold. This leads to aging and the development of age-related diseases. There are several ways to counteract these changes: (1) prevention of DNA damage through stimulation of antioxidant and detoxification systems, as well as transition metal chelation; (2) regulation of DNA methylation, chromatin structure, non-coding RNA activity and prevention of nuclear architecture alterations; (3) improving DNA damage response and repair; (4) selective removal of damaged non-functional and senescent cells. In the article, we have reviewed data about the effects of various trace elements, vitamins, polyphenols, terpenes, and other phytochemicals, as well as a number of synthetic pharmacological substances in these ways. Most of the compounds demonstrate the geroprotective potential and increase the lifespan in model organisms. However, their genome-protecting effects are non-selective and often are conditioned by hormesis. Consequently, the development of selective drugs targeting genome protection is an advanced direction.

## 1. Introduction

The accumulation of genome damage and somatic mutations leading to genome instability are important determinants and hallmarks of aging [1,2,3]. Somatic mutagenesis as a key mechanism of aging was proposed by Leo Szilard in 1959 [4]. At the same time, recent theories also explain the nature of aging by impairments in maintaining the genome functioning stability (particularly, somatic mutation catastrophe theory) [5].

The consequences of the failure of mechanisms to maintain genome stability are vividly illustrated by the pathological patterns of numerous accelerated asging syndromes that are caused by mutations in DNA repair genes (for example, Werner, Cocaine, Bloom syndromes, xeroderma pigmentosum, ataxia-telangiectasia, and others) and nuclear architecture maintenance genes (laminopathy, in particular, Hutchinson–Gilford syndrome) [6,7,8,9,10]. On the other hand, an increased expression of a number of genes, providing a response to DNA damage and repair, causes an increase in the lifespan of model animals [2,11]. Species with extreme longevity, such as naked mole rats, Brandt bats, whales, mole rat *Spalax*, and parrots have adaptive features of repair mechanisms that increase the stability of their DNA [12,13,14,15,16]. In addition, reliable DNA protection is one of the reasons for the immortality of germline cells [17]. Genome instability accompanies age-related diseases such as cancer, heart failure, type 2 diabetes, chronic obstructive pulmonary disease, stroke, Alzheimer’s disease and Parkinson’s disease, chronic kidney disease, atherosclerosis, osteoporosis, sarcopenia [7,18].

**Figure 1:**
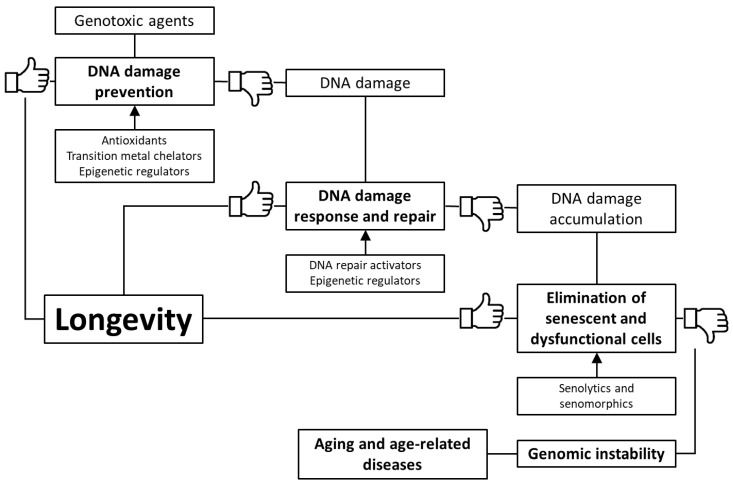
Key mechanisms of genome protection by pharmacological interventions.

Based on the foregoing, we suggest that stimulation of genome defense mechanisms may be a promising strategy to increase the lifespan and prevent the development of age-related diseases. There are several ways to achieve this goal: (1) prevention of DNA damage through stimulation of antioxidant and detoxification systems, as well as transition metal chelation; (2) regulation of DNA methylation, chromatin structure, non-coding RNA activity and prevention of nuclear architecture alterations; (3) improving DNA damage response and repair; (4) selective removal of non-functional and senescent cells (Figure 1). In the article, we have reviewed data about the genome-protecting effects of various trace elements, vitamins, polyphenols, terpenes, and other phytochemicals, as well as a number of synthetic pharmacological substances.

## 2. Impairment of the Mechanisms for Maintaining Genome Stability during Aging

Throughout life, organisms are exposed to genotoxic dangers. Sources of DNA damage and mutagenesis are a variety of external factors (including physical and chemical agents, viral infections) and intracellular causes (spontaneous hydrolytic reactions, conversion of methylated cytosine to thymine, transposition of mobile genetic elements (MGEs), reactive oxygen species (ROS), DNA replication and DNA repair errors) [2]. Switching cells from glucose metabolism to β-oxidation also increases the level of DNA damage due to lipid peroxidation [19]. In addition, the depletion of the NAD^+^ pool [20] and insufficient synthesis of nucleotide DNA [21] cause aging. Lifestyle features, such as alcohol consumption [22], tobacco smoking [23], and a disturbance of circadian rhythms can also play a negative role [24].

During aging, the frequency of DNA damage and somatic mutations in tissues of animals and humans increases, genomic instability arises, which is expressed in a burst of point mutations, breaks, cross-linking of DNA strands, transpositions, translocations, aneuploidies [2]. Application of modern methods of analysis, in particular, single-cell genome sequencing [25] and transcript sequencing [26] allows seeing the somatic mutational landscape of the human body, including the age-dependent dynamics [27]. It is worth noting that different somatic cells accumulate mutations at different rates. As a result, clones of cells with a slightly different genotype are formed in an aging organism, forming somatic mosaicism [28,29,30]. This phenomenon is extremely widespread even among healthy people [31,32].

There are several levels of the cell protection against DNA damages and the accumulation of mutations, including scavenging of DNA-damaging molecules, repair of DNA damages, and elimination of dysfunctional cells from a dividing pool in response to permanent DNA damage through the initiation of cell senescence and apoptosis. In addition, maintaining the structure of chromatin, especially constitutive heterochromatin, plays an important role in ensuring the integrity and stability of genome functioning [18,33,34,35]. At a young age, compensatory mechanisms are activated to prevent phenotypic and functional changes. However, increasing stress and age-related impairment of the functioning of these mechanisms leads to the accumulation of damage, overcoming the functional threshold [36]. Dysregulation of these pathways can lead to accelerated or premature aging, age-related decline in the functional ability of vital organs, and the development of age-related diseases.

One of the basic mechanisms for preventing damage to cell macromolecules is the antioxidant defense system. Oxidative stress leads to an age-related increase in the cellular level of oxidatively modified macromolecules, including DNA, and this increase is associated with various pathological conditions, such as aging, carcinogenesis, neurodegenerative and cardiovascular diseases. This condition is counteracted by the antioxidant defense system, which includes enzymatic (superoxide dismutase, catalase, and glutathione peroxidase, and others) and non-enzymatic (vitamins A, C, E, thiols, flavonoids, and ubiquinones) [37]. The activity of antioxidant enzymes is significantly lower at an old age compared to young, while levels of free radicals and oxidative damage to DNA are increased [38,39]. In addition, a lack of antioxidant defense systems is observed in patients with ataxia-telangiectasia and Nijmegen breakage syndrome [40].

With age, there is a decrease in the catalytic activity of DNA repair proteins, including simple repair, base excision repair (BER) and nucleotide excision repair (NER), mismatch repair (MMR), repair of double-strand breaks (DSBR) by single-stranded annealing and the non-homologous end joining (NHEJ) (but not by homologous recombination (HR)). Such changes are combined not only with a reduced ability to quickly repair damaged regions but also with an increase in the frequency of repair errors because of impaired coordination of this process. For example, impaired BER coordination can cause the formation of inappropriate apurinic/apyrimidinic sites and single-stranded structures, especially under conditions of enhanced DNA damage [2]. In addition, somatic mutations in genes involved in DNA replication and repair can lead to a feedback loop of an exponentially increasing mutational load [5].

Genome stability is also determined by the state of constitutive heterochromatin. It covers a significant part of the genome and is represented by condensed, transcriptionally inactive DNA, consisting of a large number of nucleotide repeats. In particular, centromeric and telomeric regions belong to constitutional heterochromatin. It plays a critical role in providing mitosis, DNA replication, and repair, regulating gene expression and inhibiting the activity of MGEs [33,35]. The location of constitutive heterochromatin at the periphery of the nucleus has a protective function with respect to the coding DNA in euchromatin. In the nucleus, damaging agents are absorbed, blocked, and restored by constitutive heterochromatin, and its damaged DNA is removed and excluded from the nucleus into the cytoplasm through nuclear pore complexes [34]. In the case of viral infection, due to the mechanisms of maintaining heterochromatin, there is a long-term suppression of virus replication and gene silencing at the transcription level [35]. The accumulation of DNA damage during aging is probably associated with the age-related depletion and deregulation of heterochromatin. At the same time, an increase in the total amount of heterochromatin can contribute to improving the protection of genome and DNA coding proteins [35]. The loss of constitutive heterochromatin accompanies premature aging syndromes (in particular, Werner and Hutchinson–Gilford syndromes), mediates oncogenesis, and the development of cardiovascular diseases [33,35,41].

Recently, a number of studies have demonstrated links between genomic stability, metabolism, disease, and aging, which are mediated by the NAD^+^ levels and activity of NAD^+^-dependent enzymes, such as poly(ADP-ribose) polymerases (PARPs) [42,43] and sirtuins (class III histone deacetylases (HDAC)) [44,45]. NAD^+^ declining during aging contributes to the inactivation of sirtuins [46,47], which are involved in maintaining genomic stability due to coordination of DNA repair pathways [48,49], chromatin regulation [50], and telomere maintenance [51,52]. PARPs are considered as major NAD^+^-consuming enzymes during aging [46]. These proteins are recruited by DNA single-strand breaks and initiate repair processes by auto-ADP ribosylation, which utilizes NAD^+^ [53]. PARPs-mediated NAD^+^ consumption is enhanced during aging due to increased DNA damages [43], and inhibition of PARPs activity boosts NAD^+^ levels and SIRT1 activity [42,54,55,56]. Reduction, ablation or pharmacological inhibition of PARPs increase mitochondrial metabolism and boost mitochondrial respiratory capacity. At the organism level, these changes cause beneficial effects, in particular, protection from diet-induced obesity and enhance fitness [42,54,55].

In addition to SIRT1, other chromatin-modifying proteins such as SIRT6 and the heterochromatin protein HP1 undergo age-dependent changes. Their mutations in model animals lead to a shortened lifespan, while overactivation has a geroprotective effect [35,57]. SIRT6 is an important regulator of DNA repair enzymes and a chromatin modifier in response to DNA damage; its reduction plays a critical role in genomic instability [58]. Class I HDACs also decrease their activity during aging, which is especially pronounced in the brain [59,60,61]. These proteins are assembled into the nucleosome remodeling and deacetylation complex (NuRD), which is involved in the regulation of nucleosome position, and histone deacetylase activity and controls DNA damage response [60]. A member of this class, HDAC1, provides chromatin structure maintenance as well as is essential for DNA repair and replication processes [61,62]. At the same time, enhanced activation of classes I and II HDACs causes cancer and some other chronic diseases [62,63].

Various histone methyltransferases and demethylases can also coordinate the chromatin structure and the response to DNA damage. For example, these enzymes regulate the recruitment of DNA damage response proteins to DNA lesions and provide changes in gene transcription in response to genotoxic stress. Moreover, they can interact with non-histone proteins during the response to DNA damage [64].

The depletion of constitutive heterochromatin is closely associated with the telomere shortening. The role of telomere shortening in replicative senescence is well described. Replicative DNA polymerases are not able to fully replicate telomeres. In cells with constant renewal, including embryonic cells and stem cells, the telomerase enzyme is present. It consists of reverse transcriptase (TERT) and the RNA component of telomerase (TERC) and maintains telomere length by adding de novo telomeric repeats to the ends of newly synthesized chromosomes. However, in somatic cells, telomerase in the nucleus is inactive, which leads to a cumulative loss of telomeric sequences during each division and leads to replicative senescence [7]. Telomeric dysfunction can be caused not only by the shortening of telomeres, but also by the disorder of their organization (imbalance in the formation of R-loops and guanine-quadruplexes) and by the formation of aberrant structures [65,66,67]. Abundant telomeric DNA damages contribute to genomic instability. In addition to the fact that telomeres are part of constitutive heterochromatin and are located on the periphery of the cell nucleus, their damage is not recognized by the corresponding sensors due to the presence of the shelterin complex [68,69]. In the cells of various mammalian organs, such damage accumulates, causing the formation of aging-related heterochromatic foci (SAHF) and activation of p16 [41,68,69]. In addition, TERT may be present in tissues with low replicative potential and perform non-canonical functions. It protects mitochondrial DNA from damage, maintains redox homeostasis, and protects cells from apoptosis [70,71,72].

Telomere length is not a key limiting factor in an organism lifespan [73]. This parameter varies in different tissues and cell types, and the telomere shortening rate changes over the course of an individual’s life [74,75]. At the same time, depleted telomeres are associated with an increased risk of all-cause mortality [76] and development of aging-dependent pathologies [74,77,78,79,80]. The loss of function of telomerase causes diseases characterized by premature aging, in particular, dyskeratosis congenita and its severe form, Hoyeraal–Hreidarsson syndrome [7,74,81].

As a result of the deficit of the repressive structure of constitutive heterochromatin, MGEs are activated [82,83]. They are widely represented in the eukaryotic genome (covering about 46% of the human genome; for example, *Alu*, *LINE-1*), but in the normal state, they are inactivated by transcriptional and post-transcriptional epigenetic mechanisms [84,85,86]. In the aging process, activation of MGEs occurs, which enhances genomic instability, provokes DNA damage, mutations, disruption, or change in the expression of normal genes [84,86,87].

In addition, the organization of the nuclear lamina affects the stability of the genome. A decrease in the amount of lamin B1, the accumulation of toxic levels of prelamin A and the expression of progerin (the pathogenic form of lamin A) lead to defects in the structure of the nucleus and are associated with cellular senescence and an organism aging [2,88,89]. Mutations in genes of a nuclear lamina cause premature aging syndromes called laminopathies (including Hutchinson–Gilford syndrome) [90,91]. It affects the speed of telomere shortening, the activity of genes and signaling pathways (including those associated with DNA damage response and aging), the organization of chromatin, and DNA methylation patterns [2,89]. In addition, the rigidity of the extracellular matrix through dysmorphia of the cell nucleus can provoke chromosome damages [92].

DNA damages induce a cell response that promotes the activation of signaling pathways that can drive various cell fates, including cellular senescence and apoptosis, mitochondrial dysfunction, hyperreactivity of innate immunity and inflammation [93,94,95,96].

Increasing genomic instability leads to a change in the transcription of vital genes, disruption of cellular metabolism, and causes cellular senescence. This leads to the accumulation of dysfunctional cells and genetic heterogeneity, a disruption of the regenerative potential, and physiological functions of tissues [3]. The consequences of the accumulation of DNA damages and somatic mutations are tissue-specific. In particular, the damage in macrophage DNA enhances inflammation [97], in neurons, it leads to cognitive impairment [98], in osteoprogenitor cells, it causes bone loss [99]. It is worth highlighting the accumulation of DNA damage and mutations in stem cells, as this influences their regenerative potential and creates a risk of tumor stem cells [100].

Tissue mechanisms also include a decrease in the ability of senescent cells to induce apoptosis [101] and a weakening of immunity that helps to eliminate them [102]. Cellular senescence is traditionally viewed as an irreversible cell cycle arrest that limits the proliferative potential of cells [103]. Senescent cells are involved in various physiological and pathological conditions, including tumor suppression, embryonic development, and tissue repair [104]. The senescent phenotype was described for postmitotic cells such as neurons [105], osteocytes [106], retinal cells [107], myofibrils [108] and cardiomyocytes [109].

The accumulation of senescent cells in various tissues is one of the hallmarks of aging [110] and the cause of age-dependent pathologies [111]. Cellular senescence contributes to the aging of the whole organism by reducing the regenerative potential of tissues (as a result of stem cell depletion) and through the induction of chronic inflammation (as a consequence of senescence-associated secretory phenotype (SASP) [112].

Resistance to apoptosis, in association with a decline in immune clearance, allows senescent cells to persist in the tissues for a long time, impairs tissue function, and underlies in age-related degenerative diseases, such as osteoarthritis, pulmonary fibrosis, atherosclerosis, diabetes, and Alzheimer’s disease [113]. Among the factors ensuring the resistance of senescent cells to apoptosis, ephrins (EFNB1 or 3), PI3Kδ, p21, BCL-xL, or plasminogen activator inhibitor-2 were identified [113,114].

Cellular senescence may be triggered by both external and internal stimuli [115]. External triggers arise from other senescent cells [116] and pro-inflammatory factors [117], inductors of cell proliferation (for example, growth hormone) [118], metabolic signals (for example, high glucose) [119], stress factors (for example, ionizing radiation) [120]. Internal triggers include replicative exhaustion [121] and telomere erosion [109], DNA damage [122], chromosomal instability [123], ROS [124], activation of oncogenes [125] and some other factors [93,115]. Persistent DNA damage response induces p21 and p16 cyclin-dependent kinase inhibitors and activation of the pRB retinoblastoma tumor suppressor pathway arresting the progress of the cell cycle [126,127].

## 3. Pharmacological Interventions Protecting Genome

### 3.1. Prevention of DNA Damages and Genomic Instability

The addition of exogenous antioxidants, such as vitamins A, C, E, α-lipoic acid, coenzyme Q10, glutathione, polyphenols, terpenoids, hormones, and a number of other organic compounds, as well as some minerals, including selenium, zinc, manganese can play a role in maintaining cell homeostasis and counteract the damage of cellular structures and macromolecules, including nuclear DNA [128,129] (Table 1). Firstly, a number of compounds are necessary for the proper functioning of cellular defense mechanisms; in particular, some trace elements are required for essential enzymes. For example, selenium is involved in antioxidant protection and maintenance of redox homeostasis in the form of selenoproteins (including antioxidant enzymes glutathione peroxidase, thioredoxin reductase, and selenoprotein H) [129,130,131]. Similarly, zinc is a cofactor of many enzymes, especially proteins with zinc finger domains. It is important for the functioning of Cu/Zn superoxide dismutase and metallothioneins. Zinc is an antagonist of redox transition metals such as copper or iron [132]. On the other hand, excessive concentrations of selenium and zinc have cytotoxic effects and serious consequences of organism poisoning [129,133,134]. Secondly, they can act as exogenous free radical scavengers that protect DNA molecules from oxidative damage [128,135] (Table 1). Some compounds, such as glutathione and 5,5-dimethyl-1-pyrroline-N-oxide (DMPO), can bind DNA radicals, blocking further damage propagation and cross-linking with protein molecules [136,137]. Thirdly, many biologically active compounds and pharmacological preparations stimulate the activity of internal defense systems, namely, they activate the antioxidant and detoxification enzymes [128,135]. The key role in this process is played by the activators of the KEAP1/NRF2/ARE signaling pathway, such as sulforaphane, a number of polyphenols, as well as the hormone melatonin, which has a pleiotropic effect [138] (Table 1).

Deficiency of trace elements and vitamins, which are important for antioxidant defense, often accompanies aging leading to an increase in the level of oxidative DNA damages and a predisposition to oncogenesis and the development other age-dependent diseases [132,150,192,632,633,634,635]. At the same time, supplying this deficiency has a beneficial effect on human health, especially in the elderly. The consumption of sufficient (but not excessive) amounts of vitamins and minerals maintains antioxidant profile, reduces chronic inflammation, counteracts oncogenesis and metastasis, has a neuroprotective and cardioprotective effect, supports pulmonary functions and immunity [129,150,632,633,636,637,638,639]. At the same time, in the absence of deficiency, the consumption of these substances can have a negative impact on health. 

More promising for maintaining health is the use of compounds that enhance endogenous antioxidant defense (Table 1) [640,641]. For example, these include polyphenols and terpenoids. In particular, flavonoids (quercetin, kempferol, myricetin, apigenin, luteolin, and others) and carotenoids (β-carotene, lycopene, lutein, zeaxanthin, and others) reduce the risk of cardiovascular disease (coronary disease, atherosclerosis) and cancer by eliminating ROS and protecting against DNA damage [638,639,642,643]. On the contrary, in already formed tumors, these compounds, have a cytotoxic effect and provide the sensitivity of cancer cells to treatment [644]. Biologically active substances also show a protective effect against neurodegenerative diseases (Alzheimer’s, Parkinson’s disease, as well as cerebral ischemia) due to their antioxidant effect [645]. The protective effect of phytochemicals against age-related diseases can be mediated by changes in patterns of gene expression, a decrease in chronic inflammation, and the activity of intestinal microbiota [642,646]. A pineal gland hormone and a key regulator of circadian rhythms, melatonin, is a powerful antioxidant. It protects DNA from damage by removing free radicals, chelating transition metals, coordinating redox metabolism, activating antioxidant enzymes and inhibiting prooxidant enzymes, and enhancing the effectiveness of DNA repair mechanisms [647,648]. Therefore, it can be used as an independent and additional therapy for various diseases and to improve health [649,650,651,652,653,654]. A number of pharmacological preparations (for example, metformin, rapamycin, aspirin) and synthetic compounds increase lifespan and protect against chronic diseases simultaneously with the ROS decrease and the stimulation of antioxidant defense mechanisms (Table 1). Nevertheless, this is not the main mechanism of their geroprotective action.

At the same time, the accumulated data on the geroprotective effects of antioxidants often contradict each other and indicate their inefficiency or potential genotoxic effects [128,655,656]. For example, the consumption of β-carotene, vitamin A, vitamin C, vitamin E chronically and in high doses is ineffective or has a negative effect on longevity, as was shown in studies in humans and mice [657,658,659,660]. The consumption of exogenous antioxidant substances can cause a compensatory decrease in mechanisms of endogenous defense, which cancels the general decrease in the accumulated oxidative DNA damage [658]. Their action may be due to the hormesis effect, in which small doses of these compounds cause moderate stress and stimulate the protective systems of a cell and organism. At the same time, their use at higher concentrations or for a longer time has a harmful effect [138,656]. The effects of their application largely depend on the type of cells, tissues, biochemical status, and physiological state of an organism. For example, the pro- or antioxidant effect of phytochemicals depends on the copper ion level in a cell [661]. The use of copper-trapping compounds, such as melatonin, improves antioxidant therapy [662]. At the same time, natural compounds and pharmacological substances can cause toxic effects and side effects that exceed the benefits of taking as an antioxidant supplement. For example, prolonged use of resveratrol may act as a prooxidant and adversely affect the condition and function of the thyroid gland [663]. In addition, over-treatment with antioxidants can lead to lower beneficial ROS concentrations and impaired cellular signaling [128,135].

Some biologically active compounds are able to bind and intercalate with DNA molecules. On the one hand, this allows the antioxidant to be as close as possible to the DNA site that has undergone mutagenic exposure, and it is better to perform the function of preventing or repairing the damage. On the other hand, such substances themselves can cause structural changes in the DNA molecule and at high levels provoke DNA damages and alter gene expression [367,657,664].

Another point is the rapid metabolism of phytochemicals. Often it is not the substance itself that acts on cells, but its derivatives, whose activity cannot always be predicted. Antioxidant substances can interact with each other (when used in a mixture or already present in an organism or food) and gut microbiota, which also affects their kinetics and metabolism [642,663,665]. Antioxidants consumed with food can bind to serum proteins (in particular, human serum albumin). As a result, serum proteins can modulate their concentration and the delivery of antioxidants to tissues, accumulate substances, and perform the function of their pool in an organism. Moreover, the interaction between different antioxidants can also affect their kinetics and metabolism in the liver, which leads to an increase in the level of circulating antioxidants [663,665,666]. When using various gene protective agents, it should be taken into account that there is an aging-dependent impairment of the absorption, distribution, metabolism, and functions of the consumed substances in the elderly, which is associated with a deterioration in the functions of vital organs such as the intestines, liver, and kidneys [129].

Transition and heavy metals are powerful DNA damaging agents and enhance the formation of ROS in cells. Their elimination from the body depends on the activity of antioxidant defense and detoxification systems [667]. Metal chelators also have a protective effect against genome damages. In addition to synthetic molecules, a number of polyphenolic compounds have the ability to chelate iron and copper ions [289,661] (Table 1). However, their use requires consideration of side effects. For example, metal ions are necessary for the synthesis of enzymes and the mediation of cellular chemical reactions. Therefore, their excessive removal will destabilize the functioning of cells. In particular, iron-binding tannins inhibit the activity of DNA repair enzymes [668]. Copper levels are elevated in various malignant tumors, which provides increased oxidative stress in cancer cells compared to normal cells. Some phytochemicals can increase this oxidative stress and kill tumor cells without affecting the proliferation of normal cells [669]. However, the removal of copper blocks this anti-cancer mechanism.

### 3.2. Telomere Protection

Telomere shortening prevention and telomere rejuvenation are considered as a promising anti-aging strategy. The relationship between telomere length and longevity is contradictory [73], but it is clear that depleted and dysfunctional telomeres are one of the determinants of aging [67]. Telomere attrition is associated with cancer, age-dependent diseases of the cardiovascular system (atherosclerosis, hypertension, vascular dementia, coronary heart disease, atrial fibrillation), the nervous system (dementia, Alzheimer’s disease, Parkinson’s disease, senile depression) and type 2 diabetes [74,77,78,79,80]. Telomeres are also shortened in cells of patients with syndromes of premature aging [7,74]. Therefore, therapeutic methods aimed at protecting telomeric DNA can be useful at least to reduce the risk of age-dependent pathologies.

Higher mineral and vitamin consumption is associated with longer telomeres among adults [670]. For example, folate, which provides the precursors for the synthesis of nucleotides, and vitamin B_12_ affects the integrity of telomere DNA and is associated with the length of telomeres in humans [671,672,673]. Normal folate levels are also necessary to regulate the unwinding of guanine-quadruplexes [674]. Supplementation of these vitamins to the diet delays aging in the elderly, preventing a decrease in the telomere length and the number of mitochondrial DNA copies [674].

Telomere protection can be performed by several mechanisms: reduction of the telomere DNA damage and stimulation of the expression of shelterin proteins (particularly, TIN2); prevention of the telomere shortening, and the formation of aberrant structures; increase in the telomerase activity. The ability to slow telomere shortening and activate telomerase has been shown for many natural compounds (Table 2). Most of them protect telomeric DNA by reducing damage by genotoxic agents, but their effect is small [81,675]. A promising strategy could also be coordinating the organization and stability of telomeres, for example, by targeting guanine-quadruplexes. On the other hand, these structures reduce the availability of telomeric DNA for telomerase and provide *TERT* repression (along with pro-oncogenes). Known substances that regulate guanine-quadruplexes are mainly used as anticancer treatments. They suppress telomerase activity and block cell division. Geroprotection and therapy of other diseases require the development of selective drugs [65,66].

Selective telomerase activators are more effective for telomere protection. For example, the consumption of TA-65 (a small molecule derived from *Astragalus membranaceus* extracts) leads to moderate lengthening of telomeres and improves aging-related parameters in mice and humans, but does not affect lifespan [81]. Clinical trials have shown that TA-65 in combination with vitamins improves bone density, blood pressure, metabolic markers, and macular function [57]. A positive effect was also found for sex hormones. Particularly, in mice with aplastic anemia and danazol administration [742] and in patients with telomeropathies [743], testosterone therapy led to elongation of leukocyte telomeres and improved health parameters. It is worth noting that the activation of TERT for maintaining the integrity of nuclear DNA is not relevant in all types of cells (normally, TERT is active only in embryonic and stem cells). The exogenous telomerase reactivation may be associated with a risk of oncogenesis. In cancer cells, the telomerase expression is increased by amplification and mutations of the *TERT* and *TERC* genes, changes in the methylation status of their promoters [74,81,674]. On the other hand, malignant transformation is observed mainly in cells with initially shortened telomeres and impaired structural organization [74]. Accordingly, the combination of TERT activators with substances that support its length and the correct structural organization can prevent oncogenesis. However, this approach requires careful monitoring. Gene therapy by administering TERT using an adeno-associated virus can be more effective and have a low risk of cancer. This therapy temporarily increases telomerase activity and rapidly expands telomeres, after which telomeres resume shortening, because the adeno-associated virus loses its activity after cell division [744].

TERT performs noncanonical functions and functions in mitochondria of various types of cells (including weakly proliferating and postmitotic cells). It regulates redox homeostasis and ensures the integrity of mitochondrial DNA. Thus, the activation of TERT prevents mitochondrial dysfunction, reducing the production of pathogenic ROS concentrations. As a result, its activity can indirectly prevent damage to the nuclear genome and regulate metabolic pathways [70,71,72,745]. Accordingly, exogenous stimulation of TERT gives good results in the treatment of age-dependent pathological conditions caused by mitochondrial dysfunction. For example, feeding mice with rapamycin increased the TERT activity in mitochondria in the brain and decreased the release of ROS, which at the organism level had a beneficial effect on maintaining the cognitive functions in aged animals [70,745].

### 3.3. Epidrugs and Genome Protection

Currently, compounds influencing the epigenome are coming advanced geroprotective agents (Table 3). Epigenetic modifications and their controlling proteins are attractive targets for pharmacological interventions, as they are potentially reversible and quickly respond to endogenous stimuli [128,746,747,748]. Most of the identified epidrugs have been studied in the context of their anti-cancer effects [128,749,750]. Accordingly, their effectiveness has been shown to inhibit cell proliferation and selective apoptosis. However, the use of these compounds in relation to normal cells and tissues may be useful to protect the genome from damage and deregulation [748]. A number of compounds influencing epigenetics have therapeutic potential in the treatment of cardiovascular, metabolic, and neurodegenerative diseases [749].

A balanced intake of vitamins, trace elements, and some phytochemicals have a beneficial effect on human health and prevent age-related diseases through the modulation of DNA methylation, as well as reduces biological age. For example, the co-administration of folic acid and vitamin B_12_, as well as vitamin D_3_ consumption delays the epigenetic age estimated by Horvath and Hannum methods [780,786]. At the same time, excessive consumption of certain trace elements may be associated with its increase [883]. 

Food composition can affect DNA methylation by changing the availability of methyl donors (in particular, vitamins B_6_, B_9_, B_12_, methionine, choline) and the activity of DNA methyltransferases (DNMTs) (selenium, genistein, quercetin, curcumin, green tea polyphenols, apigenin, resveratrol, sulforaphane) [748,884,885]. These compounds increase the level of DNA methylation, protecting the genome and preventing the activation of pathogenic genes. However, they do not solve the problem of hypermethylation of specific loci of genes associated with DNA repair, apoptosis, and cancer suppression [886,887,888]. Intake of vitamin A and retinoic acid, vitamin C, vitamin E, vitamin D can potentially modulate the global DNA methylation profile, histone modifications, and microRNA activity [763,774,889,890]. Polyamines spermine and spermidine stimulate the activity of DNMT and inhibit aberrant DNA methylation [891]. The geroprotective effect of certain pharmacological substances (for example, ascorbic acid and metformin) can be mediated by the modulation of TET2 methylcytosine dioxygenase [892,893]. In addition, selective inhibitors of DNMTs have been developed. However, they do not have a geroprotective effect and are applicable for the treatment of cancer addressing chemoresistance [894,895,896].

A large number of compounds are known regulators of chromatin-modifying enzymes. These include sirtuin activators (HDAC class III) and HDAC class I and II inhibitors [128,884,897]. 

Activation of sirtuins is associated with maintaining the chromatin structure, suppressing genome instability, and stimulating stress resistance mechanisms. These proteins not only determine histone acetylation but also interact with non-histone proteins that regulate aging and longevity via the insulin/IGF-1, AMPK, FOXO signaling pathways. Thereafter, sirtuin activators are considered as attractive substances for increasing lifespan and treating age-related diseases [57,898]. 

First of all, it can be achieved by restoring the deficiency of NAD^+^, for example, by vitamin B_3_ and its derivatives (particularly, nicotinamide mononucleotide), or tryptophan amino acid [20,41,848,899,900]. Pharmacological restoration of NAD^+^ bioavailability activates sirtuins, prevents age-associated metabolic decline, and promotes longevity in different animal models. A favorable outcome of NAD^+^ precursors’ application has been shown in a number of age-related diseases, including cardiovascular, metabolic, neurodegenerative disorders, sarcopenia, and muscular degeneration, osteoarthritis, visual and hearing loss, cancers and others [46,900,901,902]. Particularly, boosting NAD^+^ levels by administration of nicotinamide mononucleotide attenuates the age-associated physiological decline in mice, increases healthspan and lifespan [903]. In aged mice, this compound restores the arterial SIRT1 activity and reverses vascular dysfunction [904]. Nicotinamide riboside supplementation (a form of vitamin B3) in mice with a high-fat diet increases NAD^+^ levels and activates sirtuins, culminating in enhanced oxidative metabolism and protection against metabolic abnormalities [905]. Nicotinamide increases the cellular energy status and enhances the DNA repair activity after UV irradiation in vitro and in vivo, and prevents age-related skin changes and carcinogenesis [906]. Potential risks of using NAD^+^ precursors include the accumulation of putative toxic metabolites, oncogenesis, and stimulation of cellular senescence; their assessment requires detailed and long-term studies [900].

Expression of sirtuins is enhanced by polyphenolic compounds related to flavones, stilbenes, catechins, chalcones, and anthocyanidins (Table 3). Most of these compounds increase the lifespan of model organisms and improve the health status of patients with age-related diseases [57,128,884,897,907]. For SIRT1, the highest activity is shown for resveratrol [128,898,907]. Currently, synthetic resveratrol derivatives have been developed. They are characterized by reduced toxicity and activate SIRT1 more efficiently. At least two of them, SRT1720 and SRT2104, have proven geroprotective effects [57,128,898,907]. These compounds have demonstrated beneficial action in the treatment of aging-related diseases in preliminary clinical trials [57]. Synthetic SIRT1 activators can protect against cancer, neurodegeneration, cardiovascular and metabolic diseases, prevents degenerative changes in the bone tissue [898,908]. However, there is no evidence of their genome-protective effect and their availability to improve health and longevity in humans is unclear [57]. Other sirtuins can also serve as targets for gene-protective and geroprotective interventions. For example, an age-dependent decrease in SIRT6 is associated with cardiovascular and metabolic diseases, myopathy, liver dysfunction, and cancer [909,910]. However, the development of selective drugs to target this protein is difficult due to the structural features of the sirtuin family [907].

Class I and II HDAC inhibitors are mainly used as anti-cancer agents [894,895]. One of their effects is to increase histone acetylation and decondensation. In the context of genomic instability, the use of these compounds has a dual effect [41,748]. On the one hand, constitutive heterochromatin is important for ensuring the stability of the genome and suppressing the mutagenic activity of transposons. Chromatin decondensation makes the gene more vulnerable to genotoxic agents and can lead to its protective functions [33,34,35]. On the other hand, the discovery of areas of optional heterochromatin is important in the context of toxic effects to quickly launch compensatory mechanisms such as antioxidant defense and DNA repair [748]. Indeed, the use of HDAC inhibitors trichostatin A, vorinostat, and valproic acid stimulates various mechanisms of DNA repair (Table 4). Studies in AS52 Chinese hamster ovary cells and HeLa cells showed that a decrease in chromatin compaction after treatment with trichostatin A or butyrate slightly increases the generation of damages and does not reduce the rate of DNA repair. On the contrary, incubation of AS52 cells with resveratrol at concentrations that cause significant chromatin compaction has only a moderate effect on cell proliferation leading to a significant decrease in the DNA repair rate [837]. However, rapamycin prevents age-related epigenetic changes and maintains the structure of heterochromatin, affecting the RSC chromatin remodeling complex and HDAC expression [41]. Currently, a number of HDAC inhibitors have shown the ability to increase the lifespan of model organisms, which is accompanied by improved health and motor functions, increased activity of stress response genes (including antioxidant protection and DNA repair), and suppression of inflammation [911,912,913,914,915]. However, their gene protection and geroprotective effects require detailed study, taking into account possible toxic effects and side effects.

HDAC inhibitors can be used as medications for the treatment of age-related diseases. Their role in the suppression of carcinogenesis is well described. They increase the sensitivity of many types of cancer to chemotherapy [894,895,896]. They can also be used to treat arthritis, diabetes, heart disease, neurodegenerative diseases, and epilepsy, and HIV infection [908]. For example, the selective inhibition of certain HDACs has a pronounced neuroprotective effect, reduces the symptoms of Alzheimer’s disease in model animals and age-dependent cognitive decline [916]. However, their geno- and geroprotective effects require detailed study, taking into account possible toxic effects and side effects. In particular, inhibition of HDAC can cause skeletal abnormalities and increase bone fragility [908].

At the same time, HDAC1 activation could be effective in improving the maintenance of genomic stability and preventing the development of age-related human diseases. Recently, it has been found that HDAC1 stimulates the OGG1 DNA glycosylase, which is involved in BER and removes 8-oxoG. Pharmacological activation of HDAC1 with exifone attenuates 8-oxoG repair in old wild-type mice and in a model of Alzheimer’s disease, while HDAC1 deficiency has the opposite effect [61].

MicroRNAs are promising targets for therapeutic use. MicroRNAs play a critical role in the coordination of DNA damage response [917]. In particular, they regulate the activity of DNA damage sensors (ATM, ATR, RAD9, RAD1) and NER enzymes (RPA, XPC) [918]. Since microRNAs have multiple targets in cell networks, their regulation allows influencing signaling pathways of aging and age-related diseases [128]. Biologically active compounds can affect the activity of genes and signaling pathways associated with stress resistance, DNA repair, regulation of aging, and longevity through the activity of microRNAs [917]. MicroRNAs can be used as target molecules in the treatment of certain diseases. For example, these technologies are being developed for the treatment of cancer [919]. Currently, two main methodological approaches are used to change the activity of microRNAs. The first of them is the modulation of the microRNA function by means of overexpression based on a viral vector or synthetic double-stranded microRNAs, and the second is the inhibition of microRNAs by chemically modified antisense oligonucleotides [920]. In addition, metformin, as well as the antibiotic enoxacin, can stimulate microRNA biogenesis, which mediates their gene and geroprotective activity [921,922,923].

In addition, some compounds help maintain nuclear architecture by reducing the expression of prelamin A and progerin. However, quite a few compounds that can prevent their formation have been identified. These compounds (in particular, sulforaphane, metformin, rapamycin) cleave prelamin A and progerin by autophagic degradation [924,925] (Table 3).

### 3.4. Stimulation of DNA Repair

An important condition for ensuring genome stability is maintaining a balance of trace elements and vitamins in cells and an organism. These compounds are essential for nucleotide synthesis and DNA replication (folate, vitamin B_12_, magnesium, zinc, iron), maintenance of DNA methylation and chromosome stability (folate, vitamin B_12_), prevention of DNA oxidation (vitamin C, vitamin E, zinc, manganese, selenium), and DNA damage recognition and repair (niacin, zinc, iron, magnesium, vitamin D) (Table 4) [885,926]. Their deficiency causes DNA replication stress and genome instability, alters susceptibility to DNA damage, and provokes cellular senescence and apoptosis [885]. For example, zinc and iron-containing nutrition are necessary for the formation of enzymes with zinc finger domains and with Fe/S clusters. These enzymes include a wide range of proteins involved in DNA synthesis, DNA damage response and repair, telomere maintenance, DNA methylation, histone acetylation, and other processes important for maintaining genome stability [132,927,928]. However, excessive consumption can also have a toxic effect [129,133,883,929]. Folate and vitamin B_12_ are essential for DNA metabolism and nucleotide synthesis. Their deficiency leads to stress of DNA replication, insufficient DNA repair, DNA strand breaks, and chromosome aberrations, and results in accelerating aging of organs and tissues [930,931,932]. The application of NAD^+^ precursors is also effective in stimulating DNA repair, primarily due to improved energy metabolism and SIRT1-mediated regulation [900,902]. Supplementation of NAD^+^ precursors can improve genomic stability and health even in model animals with mutations in DNA repair genes that demonstrate its potential in the treatment of patients with premature aging syndromes [902]. Adequate intake of vitamin D_3_ and retinoic acid, which activates the DSBR, ensures the formation of a chromatin structure, supports telomere length, reduces progerin production, and helps maintain genome stability as well. Moreover, there are specific receptors that respond to vitamin levels and trigger the appropriate signaling cascades. Their induction is essential for the initiation of DNA damage response in cancers, progerias and after genotoxic exposures [789,933,934,935,936,937]. Consumption of B vitamins, vitamins C and E protects against aging-related dementia and Alzheimer’s disease through the regulation of the pathways of DNA damage response and repair [926,938].

To ensure the smooth functioning of DNA damage response systems, it is also important to maintain a balance of macronutrients (in particular, proteins and amino acids) in food and its caloric content [885]. Despite the fact that a moderate decrease in methionine and choline levels in the diet has a positive effect on lifespan and health, their critical deficiency increases the generation of DNA damages, causes significant epigenomic changes leading to organ and tissue dysfunction and carcinogenesis [980,1097,1098]. On the other hand, excessive calorie intake and being overweight are also associated with a high increase in DNA damage and inhibition of DNA repair systems, which indicates the important role of proper macronutrient intake in maintaining genome integrity [885,938,1099].

For some polyphenolic compounds (for example, curcumin, epigallocatechin gallate, resveratrol, naringenin, chrysin, quercetin, and others), the ability to reduce the level of DNA damages and stimulate the DNA damage response is described, including the regulation of sensors, transducers, and mediators [135,1000]. Proanthocyanidins and their microbial metabolites increase the expression of DNA repair genes and activate the ATM and ATR proteins [383,1029,1030]. In addition, a number of other phytochemicals and some pharmacological drugs used to treat aging-related conditions can stimulate DNA repair systems (Table 4). Inactivation of proteins involved in the DNA damage response process has been described in a number of age-dependent diseases, including cancer, as well as progeroid syndromes. Therefore, modulation of DNA repair signaling pathways directly, or through their epigenetic regulation, is one of the potential therapeutic strategies [747,1100,1101]. In particular, the brain is an organ with a high level of oxygen and energy consumption. On the one hand, this leads to an increased ROS production and a high oxidative damage level. On the other hand, it requires the supply of energy donors and coordinated energy metabolism, for example, by modulating the NAD^+^ level [1102,1103]. Targeting DNA damage repair and filling the deficiency of NAD^+^ is a promising strategy for the prevention and treatment of neurodegenerative diseases.

However, most DNA repair activators have a non-selective effect on the corresponding targets, and their effect is due to the hormetic effect (same as the activators of the antioxidant defense and detoxification systems) [138]. The development of selective drugs could be promising. However, there are a couple of pitfalls. First, a study of the effects of overactivation of DNA damage response and repair genes in model animals showed that stimulation of key regulators of DNA damage response is most effective. However, in human cells, their excessive regulation can not only stimulate the restoration of genome integrity but also provoke other reactions to genotoxic stress—cell aging and apoptosis. Secondly, the stimulation of DNA repair requires large energy investments, as well as access to the material for the assembly of nucleotides. Therefore, it is worth considering the use of adjuvant tools to fill this shortcoming [1104,1105].

### 3.5. Senolytics and Senomorphics

The pharmacological interventions that specifically target senescent cells are named senotherapeutics [1106,1107] (Table 5). Senotherapeutics are classified as senolytics, which selectively induce death of senescent cells and senomorphic (or senostatics), which block SASP [112,1107,1108].

Potential targets of senolytics are factors that ensure the resistance of senescent cells to apoptosis. Senolytics include caspase activators (piperlongumine and fisetin) [1109,1112,1129], tyrosine kinase inhibitors (dasatinib and quercetin, curcumin analogs, A-1331852, A-1155463, navitoclax) [114,1109,1110,1118,1120,1130], HSP90 inhibitors (17-DMAG, 17AAG, AT13387, BIIB021, Geldanamycin, Ganetespib, NYP-AUY922, PU-H71) [1121], FOXO4 inhibitors (FOXO4-DRI) [1119], autophagy activators (azithromycin and roxithromycin) [1115] and some other substances (Table 5).

Most of the known senolytics, except some natural compounds, have a number of undesired harmful effects that may limit their clinical applications. In addition, senescent cells are required to maintain the structure, function, and regeneration of tissues [112]. To improve the specificity and reduce the adverse effects of senolytics, drugs may be encapsulated with galactooligosaccharides, sensitive to lysosomal β-galactosidase [1131] or galactose-modified prodrugs [1132] may be used. Senolytics targeting cell-surface proteins such as DPP4 (dipeptidyl peptidase 4) [1133] and CD9 receptors [1134] enable preferential elimination of senescent cells.

Senomorphics may be free from the adverse side effects of senolytics because they target SASP without affecting the irreversible cell cycle arrest. According to the known SASP activation mechanisms, potential senomorphics targets are mTOR [1122], JAK/STAT [1123], MRE11, JNK, HDAC [1124], MDM2 [1125], p38 [1126], MK2 [1127], BRD4 [1128], GATA4 [1135], NF-κB [1126,1136], and cGAS-STING [1137] (Table 5). 

A number of senolytics and senomorphics have been proven to prevent or treat diverse age-related pathologies and diseases in animal models [1107]. Fisetin [1129,1138], the combination of dasatinib and quercetin [114], FOXO4-DRI [1119], 17-DMAG [1121], navitoclax [1130], and ruxolitinib [1139] were among the most effective compounds that reduce senescence markers in multiple tissues, restore tissue homeostasis, extend healthspan, reduce age-related pathology, and extend lifespan in progeroid or chronologically aged wild-type mice. Numerous additional anti-aging effects of senotherapeutics in human and murine cases include anti-inflammatory activity (azithromycin and ruxolitinib) [1115,1123], amelioration of lung fibrosis (digoxin) [1114], and promotion of hair regrowth (roxithromycin) [1140].

## 4. Conclusions

The aging process is accompanied by a progressive accumulation of DNA damages, epigenetic ‘DNA scars’, somatic mutations, and epimutations that provoke genomic instability. These changes cause disturbances in the activity of vital genes, disruption of cellular metabolism, and cellular senescence. As a result, dysfunctional cells accumulate in organs and tissues of an organism, inducing chronic inflammation, functional and metabolic deterioration, and the regenerative potential decreases, which condition the development of the aging process itself and risk of aging-related diseases. Preservation of the genetic stability of stem cells, which otherwise may cause aberrant differentiation or become tumor stem cells, is especially important.

Fortunately, there are a number of trace elements, vitamins, polyphenols, terpenes, polyamines, and other phytochemicals, as well as a number of synthetic pharmacological substances, that have genome-protective and geroprotective effects. Some of them are cofactors of antioxidant enzymes, DNA repair, or epigenetic regulation enzymes (in particular, Zn, Cu, Mg, NAD^+^, vitamin C, vitamin A, butyrate, glutathione). Others have free radical and advanced glycation endproduct scavenging, anti-inflammatory, heavy metal chelator effects preventing oxidative DNA damages, DNA adduct formation, as well as reducing DNA breaks and cross-linking. More promising compounds targeted on epigenetic mechanisms or stimulate pathways of DNA damage response and repair. Currently, the clinical effectiveness of their application for geroprotection and possible side effects are not clear enough and require future investigation. Unfortunately, most substances have a non-selective effect and are often conditioned by hormesis, a non-selective stress response. Furthermore, they require adjuvant therapy. Additionally, senolytics and senomorphics may be useful to eliminate or prevent the accumulation of harmful cells in an organism. However, they also need additional conditions, in particular, sufficient regenerative potential to be replaced by functional cells. Their effect is more selective but is associated with a number of side effects. For example, they can induce apoptosis of normal cells or promote the proliferation of tumor cells, increase their survival during therapy, or promote metastasis.

Consequently, the development of selective drugs or complex therapy targeted on maintaining the genome integrity and its coordinated functioning could become an advanced direction of gerontology and pharmacology.

## Figures and Tables

**Table 1:** Compounds preventing DNA damages due to the stimulation of antioxidant and detoxification systems and transition metal chelation.

Compounds	Mechanisms	References
**Trace elements**		
Selenium	Reduction of the ROS production and MAD levels. Participation in the formation of antioxidant enzymes. Activation of antioxidant enzymes (GPx, TRXR, CAT, SOD, and others). Improvement of protein levels of NRF2, HO-1, NQO-1. Metal ion chelation by selenium nanoparticles.	[139,140,141,142,143,144,145,146,147]
Zinc	Reduction of the ROS production (by NADPH oxidation inhibition). Formation of Cu/Zn SOD. Maintaining the antioxidant defense (including NRF2), improvement of the antioxidant profile. Recovery of the antioxidant enzyme activity (SOD, CAT, GPx). Protection against DNA damages induced by other trace element supplementation. Metallothionein modulation.	[141,148,149,150,151,152,153,154,155]
Iron	Suppression of the mitochondrial respiratory deficiency phenotype and decreases oxidative stress. Activation of antioxidant pathways (CAT, SOD).	[156,157,158,159]
Magnesium	Removing the excess ROS. Elevation of the activity of antioxidant enzymes.	[160]
Manganese	Protecting against ROS and MDA. Maintaining antioxidant defense, including positive regulation of NRF2 and antioxidant enzymes. Formation and enhancement of MnSOD.	[161,162]
**Vitamins and their derivatives**		
Vitamin A (retinol)	Free radical scavenging. Improvement of the activity of NRF2, HO-1, NQO-1.	[163,164,165]
Vitamin B3 (nicotinic acid, or niacin; nicotinamide; nicotinamide riboside)	The decrease in the ROS production, reduction of mitochondrial defects. Increase in the total antioxidant capacity. NRF2 activation.	[166,167,168]
Vitamin B6 (pyridoxine, pyridoxamine, pyridoxal)	The decrease in ROS and lipid peroxide levels. Improvement of the antioxidant profile. Protection of DNA integrity against hyperglycemia.	[169,170,171]
Vitamin B9 (folic acid, or folate)	The decrease in the free radical production and promotion of the activity of antioxidant enzymes. May be involved in DNA damage in the elderly.	[172,173,174,175,176,177,178]
Vitamin B12 (cobalamin)	The decrease in the free radical production and promotion of the activity of antioxidant enzymes.	[174,176]
Vitamin C (ascorbic acid)	Free radical scavenging activity. ROS production and MDA levels decrease. Protects against genotoxic damage induced by endogenous nitrosation. Activation of antioxidant enzymes (CAT, SOD, and others) and an increase in the GSH level.	[179,180,181,182,183,184]
Vitamin D3	ROS production decrease. Activation of the NRF2 signaling and the expression of antioxidant enzymes.	[185,186,187,188,189,190,191,192,193]
Vitamin E (α-, γ-, δ-tocopherols, tocotrienols)	ROS and RNS scavenging. Antioxidant activity, decrease in the MDA level. γ-Tocopherol converts nitrogen dioxide to nitric oxide. Stimulation of antioxidant enzymes and an increase in the GSH level.	[164,194,195,196,197,198,199,200,201,202,203,204]
**Coenzymes**		
Coenzyme Q10	ROS scavenging and a decrease in ROS production. Increase in the total antioxidant capacity, improvement of antioxidant status. Increase in the activity of antioxidant enzymes and GSH levels.	[205,206,207,208,209,210,211,212]
Glutathione	An electron source in enzymatic reactions as a part of antioxidant defense. Excessive ROS level suppression. It can directly intercept DNA radicals to prevent permanent DNA damage. It can inhibit the binding of potential DNA damaging agents with DNA preventing its cleavage.	[136,213,214]
**Amino acids and their derivatives**		
Trimethylglycine (betaine)	Enhancement of the total antioxidant capacity, the activity of antioxidant enzymes (SOD, CAT, GPx), an increase in the GSH content.	[215,216,217]
Carnosine	Prevention of oxidative stress and a decrease in ROS production. Free radical scavenging. Improving the antioxidant status.	[218,219,220,221,222]
L-Carnitine	ROS and MDA production decrease. Increase in the total antioxidant capacity. NRF2 activation. Improvement of the production and activity of antioxidant enzymes (SOD1, CAT, GPX1, PDRX4, and others), as well as the GSH level.	[223,224,225,226,227,228,229,230,231,232]
Histidine	Required for the maintenance of the ROS level, the activity of NRF2, and antioxidant enzymes (both deficiency and excess are harmful). Chelating divalent metal ions.	[233,234]
N-Acetylcysteine	ROS, RNS, and MDA production decrease. Enhancement of the total antioxidant capacity. Involved in glutathione synthesis, which is a cofactor of several antioxidant enzymes. Increase in the NRF2 activity and levels of antioxidant enzymes (GPx, SOD, GST) and GSH.	[173,218,219,235,236,237,238,239,240,241,242,243,244,245,246,247]
γ-Glutamylcysteine	Increases the activity of the antioxidant enzymes and total antioxidant capacity.	[248]
**Polyphenols**		
Green tea polyphenols	ROS production decrease. Restore and stimulate the functioning of antioxidant enzymes (SOD, CAT, XO, PRDX6).	[202,249,250,251,252]
Epigallocatechin gallate	Free radical scavenging activity, ROS, and MDA production decrease. Protection against genotoxic damage by endogenous nitrosation. NRF2 and antioxidant defense activation (or prevention of its impairment). Iron chelating.	[253,254,255,256,257,258,259,260,261]
Epicatechin gallate	The decrease in ROS production, scavenging free radicals. Enhancement of the activities of antioxidant enzymes and the GSH level.	[262,263,264,265]
Catechin	The decrease in the production of ROS and RNS. Stimulation of NRF2 signaling and antioxidant defense (GPx, GST). Modulation of phase 1 and 2 enzyme activities.	[266,267,268,269,270,271,272]
Epicatechin	Free radical scavenging. Metal chelating. Recovery of the antioxidant status. Modulation of phase 1 and 2 enzyme activities.	[265,268,271,273,274,275]
Theaflavin	Inhibition of the ROS and MDA generation. Activation of NRF2 and antioxidant defense enzymes (GPx, CAT, SOD). Modulation of the AKT/FOXO3a signaling. Suppression of cytochrome P450.	[271,276,277,278,279]
Apigenin	Intercalation with DNA bases. Free radical scavenging. Reduction of the ROS, MDA levels, and myeloperoxidase activity. Increase and restore of the GSH, GST, SOD, CAT, GPx levels.	[280,281,282,283,284]
Luteolin	Intercalation with DNA bases. Free radical scavenging. Enhancement of the NRF2 and HO-1 expression. Iron chelator.	[285,286,287,288,289]
Chrysin	Restoration of the antioxidant status after genotoxic treatment. Iron chelator.	[289,290,291,292]
Curcumin	Free radical scavenging. ROS and MDA production decrease. Increase in the total antioxidant capacity. Stimulation of the ARE/NRF2 signaling and antioxidant defense enzymes.	[293,294,295,296,297,298,299,300]
Quercetin	Binding with DNA bases prevents their damage. ROS scavenging, decrease in ROS production. Increase in the total antioxidant capacity. Activation of the NRF2 signaling pathway, enzymatic and non-enzymatic antioxidants. Amelioration of hyperglycemia and nitrosative stress. Iron chelating.	[258,268,301,302,303,304,305,306,307,308,309]
Rutin	Encircles and binds nucleotides preventing DNA damage. ROS scavenging. The decrease in ROS and MDA production. Stimulation of NRF2. Activation or restoration of antioxidant defense enzymes.	[183,301,309,310,311,312,313,314,315,316]
Isoquercitrin	ROS and RNS scavenging activity. Restoration of the antioxidant defense.	[310,317]
Hyperoside	ROS scavenging. MDA production decrease. SOD, CAT, GPx activation.	[318,319]
Kaempferol	Intercalation with DNA bases. The decrease in ROS and MDA levels. Activation of NRF2 and SIRT1. Increase or restoration of the expression of antioxidant enzymes (SOD1, SOD2, CAT, GPx, GCLC) and the GSH level. Suppression of cytochrome P450.	[286,320,321,322]
Myricetin	Binding with DNA bases prevents their damage. The decrease in ROS and MDA levels.	[268,301,323,324,325]
Morin	Free radical scavenging. Decreases the ROS production and content, the level of nitrites. Stimulation of the NRF2, HO-1 activity, and antioxidant defense (SOD, CAT, GSH). Iron binding and oxidation.	[326,327,328,329,330,331,332]
Fisetin	ROS scavenging and decrease in the ROS generation. Increase in GSH, GCLC levels.	[333,334,335,336]
Naringenin	ROS production decrease. Improvement of the antioxidant defense. Suppression of cytochrome P450.	[337,338,339,340,341]
Naringin	Decrease in ROS, NO, XO, MDA. Increases levels of antioxidant enzymes and GSH.	[342,343,344,345,346,347]
Hesperidin	ROS, RNS, MDA production decrease. Increase in the total antioxidant capacity. Activation of antioxidant defense enzymes.	[348,349,350,351,352,353,354]
Diosmin	ROS and RNS production decrease. Antioxidant status maintaining. Activation of antioxidant defense enzymes and GSH.	[354,355,356]
Silymarin and flavonolignans (Silybin)	ROS scavenging, decrease in the ROS generation. Activation of HO-1. Increase or restoration of the enzymatic and non-enzymatic antioxidant defense. Copper chelating agents.	[357,358,359,360,361,362,363,364,365]
Genistein	Intercalation into DNA. ROS production decrease. Free radical scavenging, including nitric oxide or peroxynitrite scavenging activities. Increase in the total antioxidant capacity. Enhancement of the NRF2 and HO-1 expression. Prevention of the antioxidant defense impairment. Induction of the expression of metallothioneins. It can chelate metabolites of polycyclic aromatic hydrocarbons.	[366,367,368,369,370,371,372,373,374,375,376]
Daidzein	Free radical scavenging, including nitric oxide or peroxynitrite scavenging activities. Increase in the total antioxidant capacity. Prevention of antioxidant defense impairment.	[366,371,374,375]
Grape seed procyanidin and proanthocyanidins	The decrease in the ROS and MDA generation. Activation of NRF2 and HO-1. Stimulation of the expression of SOD, CAT, GPx, GCLC, NQO1, and the GSH level. Metal chelating.	[377,378,379,380,381,382]
Pyrogallol	Antioxidant defense stimulation (total antioxidant capacity, GPx).	[383]
Pyrocatechol	Antioxidant defense stimulation (total antioxidant capacity, GPx).	[383]
Cyanidin	ROS generation and accumulation decrease. Inhibition of endogenous nitrosation.	[384,385,386,387]
Cyanidin-3-O-glucoside	Intercalation into DNA. The decrease in the ROS generation. Increase in the expression of NRF2 and detoxifying defense enzymes. Modulation of the GSH system. Down-regulation of the cytochrome P450 expression.	[388,389,390,391]
Pelargonidin	Inhibition of the ROS generation and endogenous nitrosation. NRF2 activation. Modulation of antioxidative and detoxification enzymes (particularly, HO-1, GST, GPx, SOD, NQO1).	[387,392,393,394,395]
Delphinidin	Suppression of the ROS formation. Restoration and activation of antioxidant and phase 2 detoxification enzymes (particularly, HO-1, GST, NQO1). Xenobiotic detoxification.	[396,397,398,399]
Honokiol	Suppression of the ROS production. Prevention of the inflammation-induced oxidative stress.	[400,401,402]
Sesamin	The decrease in the intracellular ROS and MDA production. NRF2 activation. Activation and restoration of antioxidant defense genes and enzymes (SOD, CAT, GSTD, GPx, and others), GSH level increase.	[403,404,405,406,407,408]
Sesamol	High free radical scavenging activity. The decrease in intracellular ROS production. Activation and restoration of antioxidant defense genes and enzymes (SOD, CAT, GSTD, GPx, and others), GSH level increase.	[403,409,410,411,412]
Resveratrol	Free radical scavenging activity. Recovering the nucleotide from its radical. ROS production decrease. NRF2 activation. Increases the enzymatic and non-enzymatic antioxidants status. Suppression of cytochrome P450.	[185,257,413,414,415,416,417,418,419]
Polydatin (piceid)	Free radical scavenging, inhibition of oxidative stress. Enhances the antioxidant defense.	[420,421,422,423,424]
Caffeic acid and its esters	Inhibition of the ROS generation and xanthine oxidase activity Free radical scavenging. Total antioxidant activity increase, NRF2 activation. Recovery of the GHS content and the activity of antioxidant enzymes. Iron chelating.	[303,309,425,426,427,428,429,430,431,432,433]
Chlorogenic acid	Free radical scavenging activity. The decrease in ROS production. Protection against the genotoxic damage by endogenous nitrosation. Improves the expressions of NRF2, HO-1, SOD, GSH. Iron chelating.	[253,433,434,435,436]
Rosmarinic acid	Encircling and binding nucleotides to prevent DNA damage. ROS scavenging, MDA decrease. Increase in the total antioxidant activity. Increase in the NRF2 activity and the expression of antioxidant and phase 2 detoxification enzymes, GSH level. Iron chelating.	[303,428,433,437,438,439,440]
Cinnamic acid	Increase in the antioxidant capacity. Stimulation of the activity of antioxidant enzymes. Iron chelating.	[431,441,442]
Coumaric acid	NRF2 activation. Stabilization of the antioxidant status. Blocking an increase in the xanthine oxidase activity. Iron chelating.	[431,443,444,445]
Ferulic acid	Free radical scavenging activity. Protection against the genotoxic damage by endogenous nitrosation. Activation of NRF2 and antioxidant defense enzymes. Decrease in the inflammation-induced oxidative stress. Iron chelating.	[253,309,431,433,446,447,448,449,450,451]
Salvianolic acid B	Improvement of the expressions of NRF2, HO-1, SOD, GSH.	[436]
Ellagic acid	ROS scavenging activity. Activation of NRF2, antioxidant, and phase 2 detoxification enzymes, GSH level increase. Reduction of the expression of cytochrome P450.	[452,453,454,455,456,457]
Gallic acid	Free radical scavenging activity. Protection against the genotoxic damage by endogenous nitrosation. Stimulation of the activity of antioxidant enzymes and an increase in the GSH level.	[253,458,459]
Vanillic acid	Free radical scavenging. Increase in the antioxidant capacity. Iron chelating.	[433,441]
Tannins	Iron and copper chelators. Free radical scavenging activity. Reversion of the ROS production. Stimulation of antioxidant enzymes.	[460,461,462,463,464,465,466]
Xanthohumol	ROS scavenging and improvement of the redox status. Activation of the NRF2 signaling. Induction of the glutathione related detoxification and the level of quinone reductase. The decrease in iron accumulation.	[467,468,469,470,471]
Rambutan peel phenolics	High iron and copper chelating activities. The decrease in the production of hydroxyl radical and nitric oxide.	[472]
**Terpenes and terpenoids**		
Safranal	Protection against genotoxicants. The decrease in ROS, MDA, NO levels. Improvement of the redox status. Activation of the ARE/NRF2 signaling. Improvement of the antioxidant defense, including the activity of SOD, CAT, and the GSH level.	[473,474,475,476]
Limonene	Antioxidant activity. The decrease in the MDA level. Activation of antioxidant enzymes (SOD, CAT, GPx) and GSH increasing.	[477,478,479]
Thymol	Antioxidant activity. Free radical scavenging. The decrease in ROS and MDA levels. Prevention of the decrease in SOD, CAT, GSH levels.	[480,481,482,483,484,485]
Carvacrol	Free radical scavenging. NRF2 activation. Increase in levels of GSH, GPx, SOD, CAT. Increase in metallothionein.	[481,483,486,487,488,489]
Geraniol	Protects against methylating DNA damage. Activation of SOD, CAT, GPx, GST, QR, increase in the GSH level. The decrease in the cytochrome P450 activity.	[480,490,491]
β-Caryophyllene	Decrease in oxidative and nitrative stresses. Activation of antioxidant enzymes. Antioxidant activity mediated by cannabinoid type-2 receptor activation.	[492,493,494,495]
Borneol	Iron chelating. Increase in the GSH level.	[483,496]
Ursolic acid	Free radical scavenging. The decrease in the ROS and RNS generation. Increase in the total antioxidant capacity. NRF2 activation. Improvement of the enzymatic and non-enzymatic antioxidant status.	[497,498,499,500,501]
Oleanolic acid	The decrease in the ROS, NO, MDA levels. Stimulation of antioxidant enzymes (SOD, CAT, GPx, GR) and an increase in the GSH level.	[502,503]
Lupeol	Reducing the ROS and MDA production. Prevention of DNA alkylation. Induction and restoration of the activity of antioxidant enzymes (SOD, CAT, GSH).	[504,505,506,507]
Ginsenosides	ROS scavenging. Reducing the ROS, NO, and MDA levels, ROS absorption. Improvement of the total antioxidant capacity. NRF2 activation. Activation of antioxidant and phase 2 detoxification enzymes (SOD, GPx, NQO1), GSH level increasing.	[508,509,510,511,512,513]
Gypenosides	Inhibition of the ROS production. Increase in the antioxidant enzyme activity (GST, GPx) and the GSH level.	[514,515,516,517]
Glycyrrhetinic acid	The decrease in the ROS generation. NRF2 activation.	[518,519]
Glycyrrhizic acid	Free radical scavenging. Reduction of ROS production. Restoration of levels of antioxidant enzymes and GSH.	[520,521,522]
Astaxanthin	ROS scavenging activity. Prevention of the mitochondrial dysfunction. The decrease in ROS and MDA production. Increase in the total antioxidant capacity. Activation of NRF2 and antioxidant enzymes (SOD1, SOD2), increase in the GSH level. Reduction of the expression of cytochrome P450.	[523,524,525,526,527,528]
Fucoxanthin	ROS scavenging activity. The decrease in the ROS level. Recovery of antioxidative enzymes and the GSH levels.	[529,530,531,532]
Zeaxanthin	ROS scavenging activity. The decrease in ROS, RNS, and MDA levels. Recovery and increase in the expression of antioxidant enzymes (SOD, CAT, GPx) and the GSH level.	[203,523,533,534,535]
Lutein	ROS scavenging activity. The decrease in ROS, RNS, and MDA levels. Influence on the expression of antioxidant defense genes, especially genes of oxygen transporters. Increase in the GSH level.	[203,523,535,536,537,538]
Lycopene	Reduction of ROS, NO, and MDA levels. Activation of the NRF2 and HO-1 pathways. Antioxidant enzymatic and non-enzymatic defense stimulation.	[539,540,541,542,543,544,545,546,547]
Bixin	NRF2 activator. Increase in the GSH level.	[548,549,550]
Crocin	Protection against genotoxicants. The decrease in ROS, MDA, NO levels. Increase in the total antioxidant capacity. Stimulation of SOD, CAT.	[473,551,552]
**Organic acids**		
α-Lipoic acid	ROS and MDA production decrease. Stimulation of enzymatic and non-enzymatic antioxidant defense, as well as the NRF2/ARE/ERE signaling. It can inhibit the binding of potential damaging agents with DNA preventing its cleavage. Iron chelating.	[136,208,228,257,258,553,554,555,556]
**Isothiocyanates**		
Sulforaphane	Activator of NRF2/ARE and HO-1. Restoration of levels of antioxidant and phase 2 detoxification enzymes, GSH level increase. Decrease in glucose metabolism and the level of associated enzymes.	[417,557,558,559,560,561,562,563,564,565,566]
Raphasatin	In low doses, it demonstrates anti-genotoxic and antioxidant activities.	[557,567]
**Polyamines**		
Spermine	Free radical scavenging.	[568,569]
**Alkaloids**		
Berberine	Free radical scavenging activity. Reduction of ROS, RNS, and MDA levels. Improvement of the total antioxidant capacity. Stimulation of the NRF2/HO-1 pathway, the expression of antioxidant enzymes and genes, increase in the GSH level.	[185,570,571,572,573,574,575,576]
**Indoles**		
3,3′-Diindolylmethane	The decrease in ROS and MDA levels. Activation of NRF2/ARE. Increase in the expression of HO-1, NQO1, GST, and the GSH level.	[577,578,579,580]
**Other phytochemicals**		
Vanillin and its derivatives	Free radical scavenging. The decrease in ROS and MDA levels. Modulation of enzymatic and non-enzymatic antioxidant defense.	[581,582,583,584,585]
Fucoidan	The decrease in the ROS level. Stimulation of NRF2, HO-1, and antioxidant defense enzymes. Metal ion chelating.	[586,587,588]
Eugenol and isoeugenol	ROS scavenging. The decrease in the ROS and MDA production, block the DNA oxidation. Improvement of the antioxidant status. Activation and decline prevention of antioxidant enzymes (SOD, CAT) and GSH. The decrease in the cytochrome P450 activity.	[483,589,590,591,592,593]
Chlorophyllin	The decrease in ROS and MDA levels. Activation NRF2 and antioxidant enzymes, increase in the GSH level. Reduction of the expression of cytochrome P450. Prevention of DNA fragmentation by poliovirus.	[455,594,595]
Theaphenon-E	Activation NRF2 and antioxidant enzymes. Reduction of the expression of cytochrome P450.	[455]
**Hormones**		
Melatonin	Free radical scavenging. The decrease in ROS and MDA levels. Increase in the total antioxidant capacity. NRF2 activation. Enhancement of the activity of antioxidant and phase 2 detoxification enzymes (GPx, SOD, CAT, HO-1, NQO1) and the GSH level. Copper chelating agent.	[257,596,597,598,599,600,601,602,603,604]
17β-Estradiol	Intercalation into DNA. The decrease in ROS and MDA production. Modulation of enzymatic and non-enzymatic antioxidant systems.	[246,367,605]
Raloxifene	ROS production decrease. Modulation of enzymatic and non-enzymatic antioxidant systems.	[605]
Tamoxifen	ROS production decrease. Modulation of enzymatic and non-enzymatic antioxidant systems.	[605]
**Synthetic compounds**		
Metformin	ROS production inhibition due to AMPK activation. Modulation of NRF2 and antioxidant enzymes. Reduction of the expression of cytochrome P450.	[185,606,607,608,609,610,611]
Rapamycin	Intracellular ROS production decrease. Modulation of intracellular antioxidants.	[185,612,613]
Aspirin and bis(aspirinato)zinc(II)	Free radical scavenging. The decrease in intracellular ROS production. Increase in SOD, CAT, GPx levels.	[185,614,615,616]
Alpha phenyl-tert-butyl nitrone and its derivatives	Free radical scavenging activity. The decrease in ROS production.	[182,617,618,619,620,621]
5,5-dimethyl-1-pyrroline-N-oxide (DMPO)	Scavenging of DNA radicals.	[235]
Trolox	Decrease in ROS and RNS levels. Maintaining the antioxidant status.	[235,622,623,624]
Rosuvastatin	Decrease in ROS and RNS levels. Maintaining the antioxidant status.	[235]
Valproic acid	Decrease in the ROS and MDA production. Stimulates the Nrf2/HO-1 pathway and the expression of antioxidant enzymes. But can induce DNA damages.	[625,626]
RG108	Decrease in the ROS and MDA production. Stimulation of the expression of NRF2 and antioxidant enzymes.	[626]
Ethylenediaminetetraacetic acid (EDTA)	Chelating of bivalent metals and radionuclides. Decrease in ROS and MDA levels. Maintaining the antioxidant status.	[169,426,627,628,629]
Deferoxamine (Desferal)	Iron chelator. It can act as an antioxidant in stress conditions. But can influence the DNA damage response mechanism, particularly, by inhibition of PARP.	[245,258,630,631]
Bathocuproine disulfonate	Copper chelating agent.	[604]

**Table 2:** Compounds preventing DNA damage due to epigenetic regulation, telomere maintenance, and nuclear architecture modulation.

Compounds	Mechanisms	References
**Trace elements**		
Selenium	Telomere length maintenance. Regulation of the telomerase activity in malignant and normal telomerase-positive cell types.	[676,677,678]
Zinc	The decrease in telomere damage. Increase in the telomere length, *hTERT* gene expression, and telomerase activity.	[150,679]
Iron	Increase in the telomerase activity.	[680]
Magnesium	Telomere length and telomerase activity maintenance.	[681,682,683]
**Vitamins and their derivatives**		
Nicotinamide mononucleotide (a niacin derivative)	Telomere length maintenance.	[684]
Vitamin B_9_	Telomere length maintenance. Prevention of the formation of guanine-quadruplexes in normal cells and B-lymphocytes from Werner patients.	[672,674,685,686,687]
Vitamin B_12_	Telomere length maintenance.	[672,685]
Vitamin C	Telomere length maintenance.	[180,686,688,689]
Vitamin D_3_	Prevention of the telomere shortening and induction of the telomerase activity.	[188,690]
Vitamin E	Prevention of the telomere shortening and induction of the telomerase activity.	[676,691,692]
**Coenzymes**		
Coenzyme Q10	Prevention of the telomere length shortening due to the decrease in oxidative stress.	[210,693]
Glutathione	Prevention of the telomere shortening and induction of the TERT activity.	[694]
**Amino Acids**		
Carnosine	Reduction of the telomere shortening rate and damages in telomeric DNA.	[695]
L-Carnitine	Telomere length increases. Promotion of the telomerase activity and the *hTERT* promoter methylation.	[696,697]
N-Acetylcysteine	Increasing the expression of telomerase. Reduction of the oxidative stress-induced telomere shortening, telomere fusion, and chromosomal instability.	[698,699,700,701,702]
**Polyphenols**		
Epigallocatechin gallate	Prevention of the telomere attrition and TRF2 loss.	[703,704]
Luteolin	Stabilization of the guanine-quadruplex DNA. Increase in the expression level of TERT.	[705,706]
Curcumin	Prevention of the telomere attrition, stabilization of the Guanine-quadruplex DNA. Increase in the *TERT* expression.	[707,708,709,710,711]
Quercetin	Stabilization of the guanine-quadruplex DNA. Prevention of the telomere attrition and TRF2 loss. Regulation of the TERT activity.	[704,706,712]
Rutin	Stabilization of the guanine-quadruplex DNA.	[706]
Fisetin	Regulation of the guanine-quadruplex DNA.	[713]
Silymarin and flavonolignans (Silybin)	Influence on the telomerase activity.	[714]
Genistein	Stabilization of the guanine-quadruplex DNA.	[706]
Daidzein	Stabilization of the guanine-quadruplex DNA.	[715]
Grape seed procyanidin and proanthocyanidins	Telomere length maintenance.	[716]
Delphinidin	Increase in the expression level of TERT.	[705]
Resveratrol	Telomere length and telomerase activity increase.	[717,718]
Rosmarinic acid	Telomere DNA methylation maintenance.	[719]
Ellagic acid	Stabilization of the guanine-quadruplex DNA.	[707]
**Terpenes and terpenoids**		
Oleanolic acid	Telomerase activation.	[720]
Ginsenosides	Increase in the telomere length and telomerase activity.	[721,722,723]
Astragaloside IV	Telomerase activator.	[724]
Cycloastragenol	Telomerase activator.	[724,725,726]
Zeaxanthin	Telomere length maintenance.	[688]
Lutein	Telomere length maintenance.	[688]
**Organic acids**		
α-Lipoic acid	Upregulation of PGC-1α-dependent TERT level.	[553,727]
**Hormones**		
Melatonin	Telomere maintaining and rejuvenation. Telomerase activity stimulation.	[728,729,730]
17β-Estradiol	Telomere length increases.	[731]
**Synthetic compounds**		
TA-65	Telomerase activation, elongation of short telomeres.	[732,733]
RG108	DNMT inhibitor that blocks methylation at the *TERT* promoter and increases its expression.	[734]
Farnesyltransferase inhibitor	Tethering telomeres to the nucleoskeleton.	[735]
Metformin	Increase in the TERT expression.	[736]
Rapamycin	Maintenance of the telomere length and the shelterin complex. Increase in *TIN2* and *TERT* expression. But inhibition of mTOR decreases lifespan in mice with *TERC* deficiency.	[737,738,739,740]
Aspirin	Improvement of the TERT activity.	[741]

**Table 3:** Compounds preventing DNA damage due to epigenetic regulation, telomere maintenance, and nuclear architecture modulation.

Compounds	Mechanisms	References
**Trace elements**		
Selenium	Global DNA methylation increase, LINE-1 methylation. Regulation of the expression of DNMT1, DNMT3a, DNMT3b. Influence on active and repressive histone marks. HDAC1 inhibition.	[144,751,752,753]
Zinc	Changes in the methylation status of *hTERT* promoter. Inhibition of global DNA hypomethylation and H3K9 acetylation. Increase in the metallothionein IV mRNA expression due to the reduced DNA methylation and increased H3K9ac of the promoter.	[679,754,755,756]
Iron	Modulation of DNA demethylation due to TET enzymes. Participation in the facultative heterochromatin assembly.	[757,758]
Magnesium	Mediates the nucleosome self-assembly and DNA self-assembly, heterochromatin formation.	[759,760,761]
Manganese	Influence on epigenetic modifications (particularly, reducing DNA methylation and increasing H3K9 acetylation). Inhibition of the acetylation of core histones and regulation of the activity of HDACs and HATs.	[162,762]
**Vitamins and their derivatives**		
Vitamin A	Influence on global and site-specific DNA methylation profiles.	[763,764]
Retinoic acid	Regulation of epigenetic processes, including DNA methylation, histone modifications, the formation of polycomb repressive complex 2, and induction of transcription factors. The decrease in DNMT1 and DNMT3b expression. Repressive chromatin-remodeling mediated by RIP140, G9a, and HP1γ. The decrease in the progerin and prelamin A expression.	[765,766,767,768,769,770,771,772,773,774]
Vitamin B_3_	NAD^+^ precursor, it provides sirtuin activity. HDAC III inhibitor, determines heterochromatin remodeling.	[775,776,777,778]
Nicotinamide mononucleotide (a niacin derivative)	Activation of sirtuins. Anti-aging changes in the miRNA expression profile.	[684,779]
Vitamin B_9_	Regulation of DNA methylation and heterochromatin structure. DNMT activation.	[175,674,780,781]
Vitamin B_12_	Regulation of DNA methylation.	[780]
Vitamin C	Regulation of DNA methylation due to TET activity (vitamin C is a TET co-factor). Alteration of the expression of genes involved in chromatin condensation, cell cycle regulation, DNA replication, and DNA damage repair pathways. Prevention of the heterochromatin disorganization. Inhibition of the expression of prelamin A and prevention of the nuclear lamina disorganization.	[180,782,783,784,785]
Vitamin D_3_	Association with DNA methylation age. Increase in global DNA methylation. Enhancement of the LINE-1 methylation and suppression of the endogenous retroviruses activity. Gene-specific hypomethylation and inhibition of DNMT1 и DNMT3B. NAD^+^ level decreases and SIRT1 activation. Reduction of progerin production.	[190,690,786,787,788,789,790]
Vitamin E	Increase in global DNA methylation. Induction of the MLH1 and DNMT1 gene expression.	[763,791,792]
**Amino Acids**		
Trimethylglycine (Betaine)	Modulation of global DNA methylation.	[793,794,795]
Methionine	Modulation of DNA methylation. Determination of the activity of TETs and DNMT1.	[796,797]
Choline	Modulation of DNA methylation. Determination of the activity of TETs and DNMT1.	[795,796]
N-Acetylcysteine	Reduce of DNA hypermethylation. Increase in the expression sirtuins. Prevention of chromatin decondensation. Decrease in prelamin A.	[237,698,798,799]
**Polyphenols**		
Epigallocatechin gallate	It can influence methylation patterns and the activity of DNMT1. Inhibition of DNMT1 and demethylation of the DNA repair gene promoter. HDAC inhibition and SIRT1 activation. Promotion of chromatin relaxation. Increase in histone acetylation (H3K9/14ac, H3ac), and methylation of both active (H3K4me3) and repressive (H3K9me3) chromatin marks. Influence on the expression of epigenome modulators.	[578,800,801,802,803,804]
Luteolin	HAT inhibition and SIRT1 activation.	[805]
Chrysin	Binding with the active site of SAM-dependent methyltransferase.	[806]
Curcumin	Locus-specific modulation of DNA methylation. Restoration of the DNA methylation status, expression of DNMTs, MBD4, MeCP2 after genotoxic treatment. Gene-specific hypomethylation (in the case of BRCA1 promoter) by upregulation of the *TET1* gene. Increase in the LINE-1 methylation. Influence on the SIRT1 and p300/CBP signaling pathways. It can act as an HDAC inhibitor. Modulates chromatin condensation.	[807,808,809,810,811,812,813,814]
Quercetin	Influence on the chromatin condensation and restoration of the heterochromatin architecture. Modulation of different proteins related to epigenetic modifications. SIRT1 activation and HDAC inhibitor.	[815,816,817]
Rutin	Modulation of miRNA and lncRNA profiles. Particularly, the regulation of the miRNA expression associated with DNA repair.	[818,819,820]
Hyperoside	SIRT1 activation.	[318]
Kaempferol	SIRT1 deacetylase activity stimulation.	[322]
Morin	Prevention of the chromatin condensation and hypodiploid DNA in stress conditions. Restoration of the miRNA profile.	[327,330]
Fisetin	HAT inhibition and SIRT1 activation.	[805]
Naringin	Prevention of chromatin hypercondensation.	[821,822]
Silymarin and flavonolignans (Silybin)	Stimulation of SIRT1.	[823]
Genistein	Decrease in the gene-specific DNA methylation (including tumor suppressor genes). Modulation of DNA methylation, histone modification patterns, and the activity of chromatin-remodeling proteins. Modification of the binding topology of chromatin-bound proteins.	[824,825,826,827]
Daidzein	Modulation of DNA methylation, histone modification patterns, and the activity of chromatin-remodeling proteins.	[826,827]
Grape seed procyanidin and proanthocyanidins	Mediation of the DNMT and HDAC activity. The decrease in the expression of miRNA-153 preventing the post-transcriptional repression of Nrf2 (as well as AKT and GSK-3β).	[382,828,829]
Cyanidin	SIRT6 activation.	[830]
Cyanidin-3-O-glucoside	Prevention of histone modifications.	[831]
Pelargonidin	Mediation of the DNMT and HDAC activity. The decrease in the DNA methylation in the *NRF2* promoter.	[395]
Resveratrol	Activator of SIRT1 and other sirtuins. Increases SIRT1 binding with lamin A. The decrease in the acetylation of histones and other target proteins (particularly, p53). Modulation chromatin condensation. Reversion of the activity of DNMTs and the methylation of LINE-1. Modulation of the activity of miR-135a and other miRNAs that influences the sirtuin activity and the expression of DNA repair proteins (particularly, KU70 и WRN). It causes chromatin compaction but can decrease DNA repair rates.	[717,718,832,833,834,835,836,837,838,839,840]
Ellagic acid	SIRT1 activator.	[841]
Gallic acid	Activation of TLK1 that mediates chromatin remodeling, replication, DNA damage response, and repair.	[842,843]
**Terpenes and terpenoids**		
Ursolic acid	Changes of the DNA methylation pattern and histone methylation (due to the SETD7 methyltransferase), particularly targeted on the NRF2 signaling.	[501,844]
Ginsenosides	Regulation of the NAD-PARP-SIRT signaling pathway. Up-regulation of the miR-15b expression and prevention of DNA damage by an influenza virus.	[845,846]
**Organic acids**		
α-Lipoic acid	Stimulation of SIRT1, SIRT3, and their targets (FOXO3a, PGC1β).	[553,847]
β-Hydroxybutyrate	Prevention of heterochromatin instability. Activation of SIRT1.	[848,849]
**Isothiocyanates**		
Sulforaphane	Regulation of cell cycle and DNA damage response by influencing transcription patterns and DNA methylation. HDAC inhibition, enhancement of the histone H4 acetylation status. Enhancement of the progerin clearance by autophagy.	[850,851,852,853,854]
**Polyamines**		
Spermidine	Regulation of DNA conformation and chromatin condensation. Impairment of the interaction between lamin A and CK2 promoting DNA damage repair.	[855,856]
Spermine	Regulation of DNA conformation and chromatin condensation.	[856,857]
**Hormones**		
Melatonin	Modulation of DNA methylation patterns and histone marks. Participation in the chromatin packaging. Demethylation of promoters of antioxidant defense genes (SOD1, GPx, CAT). SIRT1 activation. Inhibition of miR-24, which targets genes involved in the DNA damage response and repair, and other processes. Prevention of the inhibition of the lncRNA H19 and miR-675 that regulate DNA damage response and cellular senescence.	[730,858,859,860,861,862]
17β-Estradiol	Telomere length increases. Modulation of DNA methylation patterns, histone marks, and the activity of chromatin-remodeling proteins.	[827,863]
**Synthetic compounds**		
SRT2183	Activation of SIRT1 and stimulation of DNA damage response.	[864]
Exifone	HDAC1 activator that modulates *OGG1* expression.	[61]
Trichostatin A	HDAC inhibitor that decreases chromatin condensation. Promotion of prompt and slow DNA repair in the open chromatin conformation. Inhibition of deacetylation of H3K18 acetylation in the promoter regions of NER genes.	[865,866,867,868]
Suberoylanilide hydroxamic acid (SAHA, Vorinostat)	HDAC inhibitor that decreases chromatin condensation. Restoration of acetylation levels of H3 and H4 after irradiation. Prevention of elevated recruitment of DNMT1 and DNMT3b to the promoter of a DNA repair gene (OGG1). Abrogation of viral DNA amplification and inhibition of DNA replication in infected cells (but not in uninfected).	[869,870,871,872]
Valproic acid	HDAC inhibitor that decreases chromatin condensation. Modulation of the antioxidant defense. Initiation of DNA damage response due to histone acetylation regulation. Relaxing chromatin state stimulates immediate DNA repair. Enhancement of the DNA sensitivity to specific enzymes and increase in the interaction with intercalating agents.	[625,626,869,873,874]
RG108	DNMT inhibitor. Blocking methylation at the *TERT* promoter and increasing its expression. Modulation of the antioxidant defense. Decrease in marks of DNA damage and cellular senescence.	[626,734,875]
Farnesyltransferase inhibitor	Chromosome positioning in the nuclei.	[735]
Metformin	Restoration of the NAD^+^ level (by AMPK activation). Increase in the SIRT1 gene and protein expression, and SIRT1 promoter chromatin accessibility. The decrease in the progerin expression and alteration of the LMNA pre-mRNA splicing ratio. Normalization of the expression of microRNAs after genotoxic treatment.	[609,736,876,877,878,879,880]
Rapamycin	Slowing of epigenetic aging. Modulation of DNA methylation. Restoration of the SIRT1 localization and distribution of chromatin markers. Elicits release of the transcription factor Oct-1. Induction of the autophagic degradation of progerin and prelamin A. Recovery of chromatin-associated nuclear envelope proteins LAP2α and BAF.	[738,772,881,882]

**Table 4:** Compounds that stimulate DNA damage response and repair.

Compounds	Mechanisms	References
**Trace elements**		
Selenium	DNA damage response activation through ATM (mediated by MLH1), p53, REF1, and BRCA1. Enhancement of BER. Stimulation of MMR.	[939,940,941,942,943]
Zinc	Maintaining DNA damage response and DNA repair, improvement of the DNA repair capacity. Formation and functioning of zinc finger DNA repair protein (particularly, p53, APE, PARP-1). DNA binding of PARP-1 (associates with BER pathway) and XPA (associates with NER pathway) to chromatin.	[944,945,946,947,948]
Iron	The functioning of iron-dependent enzymes involved in DNA replication and repair, cell cycle regulation. Iron-sulfur clusters that mediate DNA damage response. GADD45α inhibition (Fe depletion leads to its activation).	[159,949,950,951]
Magnesium	Coordination of DNA replication and DNA repair. Regulation of the NHEJ pathway of DSBR break repair.	[952,953,954]
Manganese	Recovery of DNA replication after stress. Production and activation of DNA repair enzymes. Regulation of the NHEJ pathway of DSBR.	[954,955,956]
**Vitamins and their derivatives**		
Retinoic acid	Mediation of DSBR due to regulation of ATM and the NHEJ pathway. Activation of the expression of proteins of the cell cycle control (p16, p21, p27, ERK, cyclin D1, CDK2).	[765,935,957,958]
Vitamin B3 (nicotinic acid, or niacin)	DNA repair enhancing. Influence on pathways related to the cell cycle and DNA replication and repair. Activation of NER genes. Functioning regulation and activation of sirtuins and PARP. Improvement of DNA integrity in the model of Alzheimer’s disease with DNA repair deficiency.	[167,775,959,960,961,962,963]
Nicotinamide mononucleotide	Mediation of the cell cycle progression. Stimulation of the PARP-dependent DNA repair capacity.	[244,964]
Vitamin B6 (pyridoxine)	Promotion of NER (XPA, XPC expression). Cell cycle regulation.	[965,966]
Vitamin B9 (folic acid, or folate)	Mediation of MMR. DNA methylation of genes involved in DNA damage response and DNA repair (*p16, MLH1, MGMT*). Increase in the BRCA1 and BRCA2 expression.	[967,968,969]
Vitamin B12 (cobalamin)	DNA methylation of genes involved in DNA damage response and DNA repair (*p16, MLH1, MGMT*).	[967]
Vitamin C (ascorbic acid)	DNA repair enhancement. Alteration of the expression of genes involved in chromatin condensation, cell cycle regulation, DNA replication, and DNA damage repair pathways. Inhibition of DSBR-activating ATM or DNA-PK kinases.	[180,182,970]
Vitamin D3	Increase in DNA synthesis, DNA repair (particularly, repair of cyclobutane pyrimidine dimers), and energy availability. Influence on pathways related to the cell cycle, DNA replication, and repair. BRCA1 and 53BP1 stabilization. Restoration of levels of key DNA repair members belonging to DSBR sensors (MRE11, NBS1, RAD50), mediators, and effectors (CHECK2, BRCA1, RAD51). Regulation of p53-p21 and p16-Rb signaling pathways.	[186,187,933,962,971,972,973,974,975,976]
Vitamin E	Increase in the DNA repair protein expression (in particular, RAD50, GADD45α, XRCC6, PARP1).	[202,977,978]
**Amino acids and derivatives**		
Trimethylglycine (Betaine)	Enhancement of DNA repair (and *OGG1* expression) due to the regulation of DNA methylation.	[217,979]
Methionine	Requires for DNA repair and DNA methylation.	[980,981]
L-Carnitine	Enhancement of the rate and extent of DNA repair Increase in the mRNA expression of a set of DNA repair genes. Induction of the PCNA activity.	[226,982]
N-Acetylcysteine	Mediation of the cell cycle progression. Increase in the mRNA expression of a set of DNA repair genes. Activation of ATM and ATR for DNA repair. Stimulation of OGG1 and MGMT. Down-regulation of the expression of senescence markers (p16, p53).	[202,238,244,702,982,983,984,985,986]
**Polyphenols**		
Green tea polyphenols	Effects are mediated by NER and IL-12-dependent DNA repair. Stimulation of DNA repair. Increase in the expression of genes involved in different DNA repair mechanisms (*XRCC6, GADD45α, RAD51,* and others).	[202,249,987,988,989,990,991]
Epigallocatechin gallate	FOXO and SIRT1 activation. Increase in the expression of genes involved in HR, BER, NER, MMR. Suppression of DNA damage-responsive genes. Stimulation of the IL-12-and XPA- dependent DNA repair.	[801,990,992,993,994,995]
Theaflavin	Increase in the expression of DNA repair genes (*XRCC1, XRCC3, ERCC3*).	[994]
Chafuroside B	Promotion of the repair of UVB-induced DNA damage	[996]
Apigenin	Intercalation with DNA bases and ROS levels reduction. Increase in the excision of DNA damages, maintaining the expression of NER genes (*XPC, XPB, XPG, XPF, TFIIH, ERCC1*).	[273,280,997,998]
Luteolin	Increase in DNA repair capacity.	[999]
Chrysin	Activation of the ATM-Chk2 pathway in the absence of DNA damages.	[1000]
Curcumin	Activation of DNA repair genes (in particular, *CCNH* and *XRCC5*). Stimulation of TP53, BRCA1, BRCA2, ERCC1, GADD45α as well as APE1, PARP1, MGMT, and pol β.	[299,300,814,983,1001,1002,1003]
Quercetin	Non-enzymatic repair mechanism. Increase in the non-specific endonuclease activity. Stimulation of the expression of some DNA repair genes and enzymes (in particular, ATM, APE1, OGG1, XRCC1, ERCC2).	[273,308,1004,1005,1006,1007]
Rutin (troxerutin)	Maintaining DNA repair capacity due to non-enzymatic repair mechanisms and NER. In combination with podophyllotoxin (G-003M) increased levels of DNA-PK, KU80, Ligase IV, MRE11, RAD50, NBS1.	[818,1004,1008,1009]
Myricetin	Activation of BER and NHEJ. Modulation of the activity of DNA repair genes.	[1010,1011,1012,1013]
Sakuranetin	Increase in the non-specific endonuclease activity and the excision of DNA damages. Activation of NHEJ.	[273,1013]
Naringenin	Stimulation of BER, stimulation of the OGG1 expression.	[1014,1015]
Naringin	Stimulation of DNA repair.	[1014,1016]
Hesperidin	Stimulation of BER and DNA photo-damage repair.	[1014,1017]
Silymarin and flavonolignans (Silybin)	Induction of BER and NER, IL-12-dependent DNA repair. Increase in the expression of p53, DNA-PK, GADD45, XPA, XPB, XPC, XPG, as well as MGMT. Effects are mediated by *GADD45, XPA, XPB* genes. It can regulate cell cycle arrest providing a prolonged time for efficient DNA repair.	[823,1014,1018,1019,1020,1021,1022,1023]
Genistein	Preservation of proliferation and DNA repair. Enhancement of the DNA repair efficiency. Induction of the activity of proteins ATM, p53, HUS1 and others, and expression of the *GADD45* gene.	[369,1006,1024,1025,1026,1027,1028]
Daidzein	Enhancement of the DNA repair efficiency. Induction of the *GADD45* expression.	[1025,1026,1027]
Grape seed proanthocyanidins	Enhancement of the expression of DNA repair genes (*XPA, XPC, DDB2, RPA1*). The effects are mediated by XPA and its interaction with ERCC1.	[1029,1030,1031,1032]
Pyrogallol	Effect on DNA damage response proteins, particularly ATR up-regulation.	[383]
Pyrocatechol	Effect on DNA damage response proteins, particularly ATM up-regulation.	[383]
Pelargonidin	Activation of DNA repair cascades (PARP and p53).	[394]
Sesamin	Activation of the SIRT1-SIRT3-FOXO3a expression. Activation of the expression of DNA repair genes (*GADD45* and AP lyase).	[403,404]
Sesamol	Stimulation of the repair of radiation-induced damages. Activation of the SIRT1-SIRT3-FOXO3a expression.	[403,1033,1034]
Resveratrol	SIRT1 activator, regulates DNA damage repair proteins (particularly, KU70 and WRN). Directly activates ATM. Increase in the promoter activity of the *TP53* gene. Enhancement of BER (promotes APE1, OGG1, MGMT activity). Contribution to DSBR. Stimulation of the activity of tyrosyl-tRNA synthetase (TyrRS) due to SIRT1.	[839,983,1035,1036,1037,1038,1039,1040,1041]
Piceatannol	Enhancement of levels and enzymatic activity of DNA repair-related polymerases.	[1042,1043]
Caffeic acid	Stimulation of NER and the expression of XPC, XPE, TFIIH, and ERCC1 proteins.	[425]
Chlorogenic acid	Stimulation of BER.	[435]
Ferulic acid	Activation of DNA repair. Increase in NHEJ.	[447,1044]
Rosmarinic acid	Stimulation of BER and OGG1 expression.	[1045]
Ellagic acid	Enhancement of the expression of *OGG1, XPA, XPD, XPG, XRCC1, ERCC5, DNL3*, which participate in different DNA repair mechanisms. GADD45α activation.	[455,841,1046]
Gallic acid	Promotion of DNA repair, particularly, due to HR. Induction of the expression of DNA repair genes.	[459,843,1047]
Tannins	Increase in the efficacy of DNA repair systems. Induction of NER (XPC, ERCC1) and its regulation (SP1, SIRT1). However, inhibition of the activity of iron-containing enzymes (including some DNA repair enzymes).	[464,668,1048,1049]
**Terpenes and terpenoids**		
Camphor	Stimulation of error-free DNA repair (NER, MMR).	[1050,1051]
Eucalyptol	Stimulation of error-free DNA repair (NER, MMR).	[1050,1051]
Thujone	Stimulation of error-free DNA repair (NER, MMR).	[1050,1051]
Ursolic acid	Increase in the DNA repair capacity.	[999,1052]
Lupeol	Induces DNA repair genes (*hOGG1, XRCC1*).	[504]
Ginsenosides	Increase in the DNA repair capacity. Stimulation of the activity of endonucleases VIII that provides BER. Enhancement of NER and induction of levels of XPC and ERCC1.	[517,1053,1054,1055,1056,1057]
Astaxanthin	Increase in the DNA repair capacity. Recovery of the MRE11 expression. Enhancement of the expression of OGG1, XPD, XPG, XRCC1.	[455,1053,1058]
Fucoxanthin	Recovery of the MRE11 expression. Induces DNA damage response genes.	[1058,1059]
Lycopene	Reversion of alterations in cell-cycle distribution, ATM- and ATR-mediated DNA damage response. Prevention of the loss of Ku70.	[547,1060]
**Organic acids**		
α-Lipoic acid	Upregulation of the DNA repair protein, PCNA.	[1061]
**Isothiocyanates**		
Sulforaphane	Activation of DNA repair in normal cells. Enhancement of the expression of the BER protein MGMT.	[851,983,1062]
**Polyamines**		
Spermidine	Regulation of DNA repair due to DNA conformation and chromatin condensation.	[856]
Spermine	Regulation of DNA repair due to DNA conformation and chromatin condensation.	[856]
**Indoles**		
3,3′-Diindolylmethane	Stimulation of DNA damage response due to ATM activation.	[1063]
**Other compounds**		
Vanillin and its derivatives	Elicit recombinational DNA repair. Promotion of the DNA-PKcs activity. Modulation of p53.	[1064,1065,1066]
Chlorophyllin	Modulation of the activity of DNA repair genes. Enhancement of the expression of OGG1, XPD, XPG, XRCC1.	[455,1067]
Theaphenon-E	Enhancement of the expression of OGG1, XPD, XPG, XRCC1.	[455]
**Hormones**		
Melatonin	DNA repair stimulation by different mechanisms, particularly, BER, NER, and NHEJ. Increase in the expression of *OGG1, APE1, XRCC1, CDKN1a, RAD50, Ku70 XRCC4* genes. Modulation of the ATM and p53 activity.	[601,1068,1069,1070,1071,1072,1073,1074]
**Synthetic compounds**		
Trolox	Stimulation of DNA damage repair. Activation of ATM and ATR for DNA repair.	[624,984]
Metformin	Enhancement of DNA damage repair. Modulation of expression patterns of genes involved in cell cycle regulation, DNA replication, recombination, and repair. Stimulation BER and NER. Regulation of the ATM and p53 activity, an increase in the expression of *OGG1, APE1* genes, and the level of XRCC1, XPC proteins that were controlled by AMPK.	[606,607,1075,1076,1077,1078,1079,1080,1081]
Rapamycin	Regulation of cellular proliferation and PARP1 expression. Induction of DNA damage repair. Upregulation of BER repair enzyme OGG1.	[735,772,1082]
Aspirin	Regulation of the expression of genes involved in DNA damage response and repair. Protection against pathological processes in diseases associated with mutations in DNA repair genes (particularly, MMR genes and BRCA1).	[1083,1084,1085,1086,1087]
Nicorandil	Enhancement of BER, increase in the APE1 expression.	[1088]
Trichostatin A	Enhancement of the DNA repair capacity, modulation of the expression of DNA damage response, and repair genes. Promotion of the expression of NER genes (XPA, XPD, XPF). Improvement of DSBR. Activation of DNA-PK. Enhancement of NHEJ (but inhibition of HR). Improvement of the Ing1-mediated DNA damage response.	[865,866,867,868,1089,1090,1091,1092]
Suberoylanilide hydroxamic acid (SAHA, Vorinostat)	Modulation of the expression of genes involved in BER, NER, MMR, DSBR, but enhancement of DNA damages in mesenchymal stem cells. Amelioration of DNA repair efficiency. OGG1 activation by DNA demethylation and HDAC inhibition. Improvement of functions in a model of Cockayne syndrome.	[870,871,1093,1094]
Valproic acid	Modulation of the expression of genes involved in cell cycle control and DNA repair. Stimulation of immediate DNA repair.	[869,1095]
Farnesyltransferase inhibitor	Stimulation of DSBR.	[1096]
Enoxacin	Stimulation of DSBR (NHEJ) due to the formation of DNA damage response RNAs and the recruiting of DNA repair enzymes.	[923]

**Table 5:** Compounds with senotherapeutic potential.

Compounds	Mechanisms	References
**Senolytics**		
**Polyphenols**		
Fisetin	Activates caspases-7,8 and 9.	[1109]
Quercetin	Inhibits PI3K, other kinases, and serpines.	[114]
EF24 (curcumin analog)	Downregulates the Bcl-2 family proteins.	[1110]
Apigenin	NF-κB p65 inhibitor.	[1111]
Kaempferol	NF-κB p65 inhibitor.	[1111]
**Alkaloids**		
Piperlongumine	Activates caspase-3. Degradation of PARP.	[1112]
**Cardiac glycosides**		
Ouabain	Inhibitor of Na^+^/K^+^ ATPase on the plasma membrane.	[1113]
Digoxin	Inhibitor of Na^+^/K^+^ ATPase on the plasma membrane.	[1114]
**Synthetic compounds**		
Dasatinib	Inhibitor of multiple tyrosine kinases (alone and in the combination with Quercetin).	[114]
Azithromycin	Induces both aerobic glycolysis and autophagy.	[1115]
Fenofibrate	PPARα agonist.	[1116]
Panobinostat	Non-selective HDAC inhibitor.	[1117]
ABT-737	BCL-2, BCL-W, and BCL-XL inhibitor.	[1118]
A-1331852	Selective BCL-X_L_ inhibitor.	[1109]
A-1155463	Selective BCL-X_L_ inhibitor.	[1109]
FOXO4-DRI (modified FOXO4-p53 interfering peptide)	It causes p53 nuclear exclusion and cell-intrinsic apoptosis.	[1119]
Navitoclax (ABT-263)	Bcl-2 family inhibitor.	[1120]
17-DMAG	HSP90 inhibitor.	[1121]
17AAG	HSP90 inhibitor.	[1121]
AT13387	HSP90 inhibitor.	[1121]
BIIB021	HSP90 inhibitor.	[1121]
Geldanamycin	HSP90 inhibitor.	[1121]
Ganetespib	HSP90 inhibitor.	[1121]
NYP-AUY922	HSP90 inhibitor.	[1121]
PU-H71	HSP90 inhibitor.	[1121]
**Senomorphics**		
**Synthetic compounds**		
Rapamycin	mTOR inhibitor.	[1122]
Ruxolitinib	JAK inhibitor.	[1123]
Trichostatin A and Vorinostat	HDAC inhibitors.	[1124]
Mirin	Inhibitor of MRE11-mediated end resection.	[1124]
SP600125	JNK inhibitor.	[1124]
Nutlin-3a	MDM2 inhibitors.	[1125]
MI-63	MDM2 inhibitors.	[1125]
SB203580	p38 inhibitor.	[1126]
UR-13756	p38 inhibitor.	[1127]
BIRB 796	p38 inhibitor.	[1127]
PF-3644022	MK2 inhibitor.	[1127]
MK2.III	MK2 inhibitor.	[1127]
JQ1	BRD4 inhibitor.	[1128]
I-BET762	BRD4 inhibitor.	[1128]

## Abbreviations

ROS	Reactive oxygen species
RNS	Reactive nitrogen species
MAD	Malonic dialdehyde
MGE	Mobile genetic elements
BER	Base excision repair
NER	Nucleotide excision repair
MMR	Mismatch repair
DSBR	Repair of double-strand breaks
NHEJ	Non-homologous end joining
HR	Homologous recombination
DNA-PK	DNA-dependent protein kinase
DNMT	DNA methyltransferase
TET	Tet methylcytosine dioxygenase
HDAC	Histone deacetylase
HAT	Histone acetyltransferase
SASP	Senescence-associated secretory phenotype
PARP	Poly(ADP-ribose) polymerase
NAD^+^	Nicotinamide adenine dinucleotide

## References

[1] López-Otín C., Blasco M.A., Partridge L., Serrano M., Kroemer G. (2013). The hallmarks of aging. Cell.

[2] Moskalev A.A., Shaposhnikov M.V., Plyusnina E.N., Zhavoronkov A., Budovsky A., Yanai H., Fraifeld V.E. (2013). The role of DNA damage and repair in aging through the prism of Koch-like criteria. Ageing Res. Rev..

[3] Niedernhofer L.J., Gurkar A.U., Wang Y., Vijg J., Hoeijmakers J.H.J., Robbins P.D. (2018). Nuclear Genomic Instability and Aging. Annu. Rev. Biochem..

[4] Szilard L. (1959). On the nature of the aging process. Proc. Natl. Acad. Sci. USA.

[5] Milholland B., Suh Y., Vijg J. (2017). Mutation and catastrophe in the aging genome. Exp. Gerontol..

[6] Burtner C.R., Kennedy B.K. (2010). Progeria syndromes and ageing: What is the connection?. Nat. Rev. Mol. Cell Biol..

[7] Kubben N., Misteli T. (2017). Shared molecular and cellular mechanisms of premature ageing and ageing-associated diseases. Nat. Rev. Mol. Cell Biol..

[8] Keijzers G., Bakula D., Scheibye-Knudsen M. (2017). Monogenic Diseases of DNA Repair. N. Engl. J. Med..

[9] Zhavoronkov A., Smit-McBride Z., Guinan K.J., Litovchenko M., Moskalev A. (2012). Potential therapeutic approaches for modulating expression and accumulation of defective lamin A in laminopathies and age-related diseases. J. Mol. Med..

[10] Cenni V., Capanni C., Mattioli E., Schena E., Squarzoni S., Bacalini M.G., Garagnani P., Salvioli S., Franceschi C., Lattanzi G. (2020). Lamin A involvement in ageing processes. Ageing Res. Rev..

[11] Proshkina E.N., Shaposhnikov M.V., Sadritdinova A.F., Kudryavtseva A.V., Moskalev A.A. (2015). Basic mechanisms of longevity: A case study of Drosophila pro-longevity genes. Ageing Res. Rev..

[12] Petruseva I.O., Evdokimov A.N., Lavrik O.I. (2017). Genome Stability Maintenance in Naked Mole-Rat. Acta Nat..

[13] Seim I., Fang X., Xiong Z., Lobanov A.V., Huang Z., Ma S., Feng Y., Turanov A.A., Zhu Y., Lenz T.L. (2013). Genome analysis reveals insights into physiology and longevity of the Brandt’s bat Myotis brandtii. Nat. Commun..

[14] Keane M., Semeiks J., Webb A.E., Li Y.I., Quesada V., Craig T., Madsen L.B., van Dam S., Brawand D., Marques P.I. (2015). Insights into the evolution of longevity from the bowhead whale genome. Cell Rep..

[15] Schmidt H., Malik A., Bicker A., Poetzsch G., Avivi A., Shams I., Hankeln T. (2017). Hypoxia tolerance, longevity and cancer-resistance in the mole rat Spalax—A liver transcriptomics approach. Sci. Rep..

[16] Wirthlin M., Lima N., Guedes R., Soares A., Almeida L., Cavaleiro N.P., Loss de Morais G., Chaves A.V., Howard J.T., Teixeira M.M. (2018). Parrot Genomes and the Evolution of Heightened Longevity and Cognition. Curr. Biol..

[17] Bhargava V., Goldstein C.D., Russell L., Xu L., Ahmed M., Li W., Casey A., Servage K., Kollipara R., Picciarelli Z. (2020). GCNA Preserves Genome Integrity and Fertility across Species. Dev. Cell.

[18] Tiwari V., Wilson D.M. (2019). DNA Damage and Associated DNA Repair Defects in Disease and Premature Aging. Am. J. Hum. Genet..

[19] Cardoso A.C., Pereira A., Sadek H.A. (2020). Mitochondrial substrate utilization regulates cardiomyocyte cell-cycle progression. Nat. Metab..

[20] Mendelsohn A.R., Larrick J.W. (2017). The NAD+/PARP1/SIRT1 Axis in Aging. Rejuvenation Res..

[21] Hämäläinen R.H., Landoni J.C., Ahlqvist K.J., Goffart S., Ryytty S., Rahman M.O., Brilhante V., Icay K., Hautaniemi S., Wang L. (2019). Defects in mtDNA replication challenge nuclear genome stability through nucleotide depletion and provide a unifying mechanism for mouse progerias. Nat. Metab..

[22] Hämäläinen R.H., Hodskinson M.R., Bolner A., Sato K., Kamimae-Lanning A.N., Rooijers K., Witte M., Mahesh M., Silhan J., Petek M. (2020). Alcohol-derived DNA crosslinks are repaired by two distinct mechanisms. Nature.

[23] Yoshida K., Gowers K., Lee-Six H., Chandrasekharan D.P., Coorens T., Maughan E.F., Beal K., Menzies A., Millar F.R., Anderson E. (2020). Tobacco smoking and somatic mutations in human bronchial epithelium. Nature.

[24] Cheung V., Yuen V.M., Wong G.T.C., Choi S.W. (2019). The effect of sleep deprivation and disruption on DNA damage and health of doctors. Anaesthesia.

[25] Zhang L., Dong X., Lee M., Maslov A.Y., Wang T., Vijg J. (2019). Single-cell whole-genome sequencing reveals the functional landscape of somatic mutations in B lymphocytes across the human lifespan. Proc. Natl. Acad. Sci. USA.

[26] García-Nieto P.E., Morrison A.J., Fraser H.B. (2019). The somatic mutation landscape of the human body. Genome Biol..

[27] Zhang L., Vijg J. (2018). Somatic Mutagenesis in Mammals and Its Implications for Human Disease and Aging. Annu. Rev. Genet..

[28] De S. (2011). Somatic mosaicism in healthy human tissues. Trends Genet..

[29] Risques R.A., Kennedy S.R. (2018). Aging and the rise of somatic cancer-associated mutations in normal tissues. PLoS Genet..

[30] Forsberg L.A., Gisselsson D., Dumanski J.P. (2017). Mosaicism in health and disease—Clones picking up speed. Nat. Rev. Genet..

[31] Young A.L., Challen G.A., Birmann B.M., Druley T.E. (2016). Clonal haematopoiesis harbouring AML-associated mutations is ubiquitous in healthy adults. Nat. Commun..

[32] Krimmel J.D., Schmitt M.W., Harrell M.I., Agnew K.J., Kennedy S.R., Emond M.J., Loeb L.A., Swisher E.M., Risques R.A. (2016). Ultra-deep sequencing detects ovarian cancer cells in peritoneal fluid and reveals somatic TP53 mutations in noncancerous tissues. Proc. Natl. Acad. Sci. USA.

[33] Janssen A., Colmenares S.U., Karpen G.H. (2018). Heterochromatin: Guardian of the Genome. Annu. Rev. Cell Dev. Biol..

[34] Qiu G.-H., Huang C., Zheng X., Yang X. (2018). The protective function of noncoding DNA in genome defense of eukaryotic male germ cells. Epigenomics.

[35] Qiu G.H., Zheng X., Fu M., Huang C., Yang X. (2019). The protective function of non-coding DNA in DNA damage accumulation with age and its roles in age-related diseases. Biogerontology.

[36] Ferrucci L., Gonzalez-Freire M., Fabbri E., Simonsick E., Tanaka T., Moore Z., Salimi S., Sierra F., de Cabo R. (2020). Measuring biological aging in humans: A quest. Aging Cell.

[37] Olinski R., Siomek A., Rozalski R., Gackowski D., Foksinski M., Guz J., Dziaman T., Szpila A., Tudek B. (2007). Oxidative damage to DNA and antioxidant status in aging and age-related diseases. Acta Biochim. Pol..

[38] Reddy K.K., Reddy T.P., Somasekharaiah B.V., Kumarl K.S. (1998). Changes in antioxidant enzyme levels and DNA damage during aging. Indian J. Clin. Biochem..

[39] Humphreys V., Martin R.M., Ratcliffe B., Duthie S., Wood S., Gunnell D., Collins A.R. (2007). Age-related increases in DNA repair and antioxidant protection: A comparison of the Boyd Orr Cohort of elderly subjects with a younger population sample. Age Ageing.

[40] Maciejczyk M., Heropolitanska-Pliszka E., Pietrucha B., Sawicka-Powierza J., Bernatowska E., Wolska-Kusnierz B., Pac M., Car H., Zalewska A., Mikoluc B. (2019). Antioxidant Defense, Redox Homeostasis, and Oxidative Damage in Children with Ataxia Telangiectasia and Nijmegen Breakage Syndrome. Front. Immunol..

[41] Kane A.E., Sinclair D.A. (2019). Epigenetic changes during aging and their reprogramming potential. Crit. Rev. Biochem. Mol. Biol..

[42] Bai P., Cantó C., Oudart H., Brunyánszki A., Cen Y., Thomas C., Yamamoto H., Huber A., Kiss B., Houtkooper R.H. (2011). PARP-1 inhibition increases mitochondrial metabolism through SIRT1 activation. Cell Metab..

[43] Kim M.Y., Zhang T., Kraus W.L. (2005). Poly(ADP-ribosyl)ation by PARP-_1_: ‘PAR-laying’ NAD^+^ Into a Nuclear Signal. Genes Dev..

[44] Klein M.A., Liu C., Kuznetsov V.I., Feltenberger J.B., Tang W., Denu J.M. (2020). Mechanism of Activation for the Sirtuin 6 Protein Deacylase. J. Biol. Chem..

[45] Yaku K., Okabe K., Nakagawa T. (2018). NAD Metabolism: Implications in Aging and Longevity. Ageing Res. Rev..

[46] Mouchiroud L., Houtkooper R.H., Moullan N., Katsyuba E., Ryu D., Cantó C., Mottis A., Jo Y.S., Viswanathan M., Schoonjans K. (2013). The NAD^+^/Sirtuin Pathway Modulates Longevity through Activation of Mitochondrial UPR and FOXO Signaling. Cell.

[47] Imai S., Guarente L. (2014). NAD^+^ and Sirtuins in Aging and Disease. Trends Cell Biol..

[48] Palacios J.A., Herranz D., De Bonis M.L., Velasco S., Serrano M., Blasco M.A. (2010). SIRT1 Contributes to Telomere Maintenance and Augments Global Homologous Recombination. J. Cell Biol..

[49] Lombard D.B., Chua K.F., Mostoslavsky R., Franco S., Gostissa M., Alt F.W. (2005). DNA Repair, Genome Stability, and Aging. Cell.

[50] Vaquero A. (2009). The Conserved Role of Sirtuins in Chromatin Regulation. Int. J. Dev. Biol..

[51] Jia G., Su L., Singhal S., Liu X. (2012). Emerging Roles of SIRT6 on Telomere Maintenance, DNA Repair, Metabolism and Mammalian Aging. Mol. Cell. Biochem..

[52] Michishita E., McCord R.A., Berber E., Kioi M., Padilla-Nash H., Damian M., Cheung P., Kusumoto R., Kawahara T.L., Barrett J.C. (2008). SIRT6 Is a Histone H3 Lysine 9 Deacetylase That Modulates Telomeric Chromatin. Nature.

[53] Eustermann S., Wu W.F., Langelier M.F., Yang J.C., Easton L.E., Riccio A.A., Pascal J.M., Neuhaus D. (2015). Structural Basis of Detection and Signaling of DNA Single-Strand Breaks by Human PARP-1. Mol. Cell.

[54] Pirinen E., Cantó C., Jo Y.S., Morato L., Zhang H., Menzies K.J., Williams E.G., Mouchiroud L., Moullan N., Hagberg C. (2014). Pharmacological Inhibition of poly(ADP-ribose) Polymerases Improves Fitness and Mitochondrial Function in Skeletal Muscle. Cell Metab..

[55] Bai P., Canto C., Brunyánszki A., Huber A., Szántó M., Cen Y., Yamamoto H., Houten S.M., Kiss B., Oudart H. (2011). PARP-2 Regulates SIRT1 Expression and Whole-Body Energy Expenditure. Cell Metab..

[56] Fang E.F., Scheibye-Knudsen M., Brace L.E., Kassahun H., SenGupta T., Nilsen H., Mitchell J.R., Croteau D.L., Bohr V.A. (2014). Defective Mitophagy in XPA via PARP-1 Hyperactivation and NAD^+^/SIRT1 Reduction. Cell.

[57] Martel J., Ojcius D.M., Ko Y.-F., Chang C.-J., Young J.D. (2019). Antiaging effects of bioactive molecules isolated from plants and fungi. Med. Res. Rev..

[58] Chang A.R., Ferrer C.M., Mostoslavsky R. (2020). SIRT6, a Mammalian Deacylase with Multitasking Abilities. Physiol. Rev..

[59] Zupkovitz G., Lagger S., Martin D., Steiner M., Hagelkruys A., Seiser C., Schöfer C., Pusch O. (2018). Histone deacetylase 1 expression is inversely correlated with age in the short-lived fish Nothobranchius furzeri. Histochem. Cell Biol..

[60] Pegoraro G., Kubben N., Wickert U., Göhler H., Hoffmann K., Misteli T. (2009). Ageing-related chromatin defects through loss of the NURD complex. Nat. Cell Biol..

[61] Pao P.C., Patnaik D., Watson L.A., Gao F., Pan L., Wang J., Adaikkan C., Penney J., Cam H.P., Huang W.C. (2020). HDAC1 Modulates OGG1-initiated Oxidative DNA Damage Repair in the Aging Brain and Alzheimer’s Disease. Nat. Commun..

[62] Bhaskara S. (2015). Histone deacetylases 1 and 2 regulate DNA replication and DNA repair: Potential targets for genome stability-mechanism-based therapeutics for a subset of cancers. Cell Cycle.

[63] Walsh M.E., van Remmen H. (2016). Emerging roles for histone deacetylases in age-related muscle atrophy. Nutr. Healthy Aging.

[64] Chen Y., Zhu W.-G. (2016). Biological function and regulation of histone and non-histone lysine methylation in response to DNA damage. Acta Biochim. Biophys. Sin..

[65] Tan J., Lan L. (2020). The DNA Secondary Structures at Telomeres and Genome Instability. Cell Biosci..

[66] Varshney D., Spiegel J., Zyner K., Tannahill D., Balasubramanian S. (2020). The Regulation and Functions of DNA and RNA G-quadruplexes. Nat. Rev. Mol. Cell Biol..

[67] Boccardi V., Cari L., Nocentini G., Riccardi C., Cecchetti R., Ruggiero C., Arosio B., Paolisso G., Herbig U., Mecocci P. (2020). Telomeres Increasingly Develop Aberrant Structures in Aging Humans. J. Gerontol. Ser. A Biol. Sci. Med. Sci..

[68] Hewitt G., Jurk D., Marques F.D., Correia-Melo C., Hardy T., Gackowska A., Anderson R., Taschuk M., Mann J., Passos J.F. (2012). Telomeres are favoured targets of a persistent DNA damage response in ageing and stress-induced senescence. Nat. Commun..

[69] Fumagalli M., Rossiello F., Clerici M., Barozzi S., Cittaro D., Kaplunov J.M., Bucci G., Dobreva M., Matti V., Beausejour C.M. (2012). Telomeric DNA damage is irreparable and causes persistent DNA-damage-response activation. Nat. Cell Biol..

[70] Zheng Q., Huang J., Wang G. (2019). Mitochondria, Telomeres and Telomerase Subunits. Front. Cell Dev. Biol..

[71] Moro L. (2019). Mitochondrial Dysfunction in Aging and Cancer. J. Clin. Med..

[72] Rosen J., Jakobs P., Ale-Agha N., Altschmied J., Haendeler J. (2020). Non-canonical functions of Telomerase Reverse Transcriptase—Impact on redox homeostasis. Redox Biol..

[73] de Magalhães J.P., Passos J.F. (2018). Stress, cell senescence and organismal ageing. Mech. Ageing Dev..

[74] Turner K.J., Vasu V., Griffin D.K. (2019). Telomere Biology and Human Phenotype. Cells.

[75] Lin Y., Damjanovic A., Metter E.J., Nguyen H., Truong T., Najarro K., Morris C., Longo D.L., Zhan M., Ferrucci L. (2015). Age-associated telomere attrition of lymphocytes in vivo is co-ordinated with changes in telomerase activity, composition of lymphocyte subsets and health conditions. Clin. Sci..

[76] Wang Q., Zhan Y., Pedersen N.L., Fang F., Hägg S. (2018). Telomere Length and All-Cause Mortality: A Meta-analysis. Ageing Res. Rev..

[77] Kuszel L., Trzeciak T., Richter M., Czarny-Ratajczak M. (2015). Osteoarthritis and telomere shortening. J. Appl. Genet..

[78] Carlquist J.F., Knight S., Cawthon R.M., Le V.T., Jared Bunch T., Horne B.D., Rollo J.S., Huntinghouse J.A., Brent Muhlestein J., Anderson J.L. (2016). Shortened telomere length is associated with paroxysmal atrial fibrillation among cardiovascular patients enrolled in the Intermountain Heart Collaborative Study. Heart Rhythm..

[79] Hunt S.C., Kimura M., Hopkins P.N., Carr J.J., Heiss G., Province M.A., Aviv A. (2015). Leukocyte telomere length and coronary artery calcium. Am. J. Cardiol..

[80] Boccardi M., Boccardi V. (2019). Psychological Wellbeing and Healthy Aging: Focus on Telomeres. Geriatrics.

[81] Martínez P., Blasco M.A. (2017). Telomere-driven diseases and telomere-targeting therapies. J. Cell Biol..

[82] Wood J.G., Helfand S.L. (2013). Chromatin structure and transposable elements in organismal aging. Front. Genet..

[83] De Cecco M., Criscione S.W., Peckham E.J., Hillenmeyer S., Hamm E.A., Manivannan J., Peterson A.L., Kreiling J.A., Neretti N., Sedivy J.M. (2013). Genomes of replicatively senescent cells undergo global epigenetic changes leading to gene silencing and activation of transposable elements. Aging Cell.

[84] Cardelli M. (2018). The epigenetic alterations of endogenous retroelements in aging. Mech. Ageing Dev..

[85] Lenart P., Novak J., Bienertova-Vasku J. (2018). PIWI-piRNA pathway: Setting the pace of aging by reducing DNA damage. Mech. Ageing Dev..

[86] Andrenacci D., Cavaliere V., Lattanzi G. (2020). The role of transposable elements activity in aging and their possible involvement in laminopathic diseases. Ageing Res. Rev..

[87] Buzdin A.A., Prassolov V., Garazha A.V. (2017). Friends-Enemies: Endogenous Retroviruses Are Major Transcriptional Regulators of Human DNA. Front. Chem..

[88] Mattioli E., Andrenacci D., Garofalo C., Prencipe S., Scotlandi K., Remondini D., Gentilini D., Di Blasio A.M., Valente S., Scarano E. (2018). Altered modulation of lamin A/C-HDAC2 interaction and p21 expression during oxidative stress response in HGPS. Aging Cell.

[89] Ashapkin V.V., Kutueva L.I., Kurchashova S.Y., Kireev I.I. (2019). Are There Common Mechanisms Between the Hutchinson-Gilford Progeria Syndrome and Natural Aging?. Front. Genet..

[90] Worman H.J. (2012). Nuclear lamins and laminopathies. J. Pathol..

[91] Romero-Bueno R., de la Cruz Ruiz P., Artal-Sanz M., Askjaer P., Dobrzynska A. (2019). Nuclear Organization in Stress and Aging. Cells.

[92] Cho S., Vashisth M., Abbas A., Majkut S., Vogel K., Xia Y., Ivanovska I.L., Irianto J., Tewari M., Zhu K. (2019). Mechanosensing by the Lamina Protects against Nuclear Rupture, DNA Damage, and Cell-Cycle Arrest. Dev. Cell.

[93] Hernandez-Segura A., Nehme J., Demaria M. (2018). Hallmarks of Cellular Senescence. Trends Cell Biol..

[94] Zhang L., Yousefzadeh M.J., Suh Y., Niedernhofer L.J., Robbins P.D. (2019). Signal Transduction, Ageing and Disease. Sub Cell. Biochem..

[95] Brace L.E., Vose S.C., Stanya K., Gathungu R.M., Marur V.R., Longchamp A., Treviño-Villarreal H., Mejia P., Vargas D., Inouye K. (2016). Increased oxidative phosphorylation in response to acute and chronic DNA damage. NPJ Aging Mech. Dis..

[96] Nakad R., Schumacher B. (2016). DNA Damage Response and Immune Defense: Links and Mechanisms. Front. Genet..

[97] Goulielmaki E., Ioannidou A., Tsekrekou M., Stratigi K., Poutakidou I.K., Gkirtzimanaki K., Aivaliotis M., Evangelou K., Topalis P., Altmüller J. (2020). Tissue-infiltrating macrophages mediate an exosome-based metabolic reprogramming upon DNA damage. Nat. Commun..

[98] Shanbhag N.M., Evans M.D., Mao W., Nana A.L., Seeley W.W., Adame A., Rissman R.A., Masliah E., Mucke L. (2019). Early neuronal accumulation of DNA double strand breaks in Alzheimer’s disease. Acta Neuropathol. Commun..

[99] Kim H.N., Chang J., Shao L., Han L., Iyer S., Manolagas S.C., O’Brien C.A., Jilka R.L., Zhou D., Almeida M. (2017). DNA damage and senescence in osteoprogenitors expressing Osx1 cause their decrease with age. Aging Cell.

[100] Walter D., Lier A., Geiselhart A., Thalheimer F.B., Huntscha S., Sobotta M.C., Moehrle B., Brocks D., Bayindir I., Kaschutnig P. (2015). Exit from dormancy provokes DNA-damage-induced attrition in haematopoietic stem cells. Nature.

[101] Huang W.T., Akhter H., Jiang C., MacEwen M., Ding Q., Antony V., Thannickal V.J., Liu R.M. (2015). Plasminogen activator inhibitor 1, fibroblast apoptosis resistance, and aging-related susceptibility to lung fibrosis. Exp. Gerontol..

[102] Soria-Valles C., López-Soto A., Osorio F.G., López-Otín C. (2017). Immune and inflammatory responses to DNA damage in cancer and aging. Mech. Ageing Dev..

[103] Campisi J., d’Adda di Fagagna F. (2007). Cellular senescence: When bad things happen to good cells. Nat. Rev. Mol. Cell Biol..

[104] Aravinthan A. (2015). Cellular senescence: A hitchhiker’s guide. Hum. Cell.

[105] Jurk D., Wang C., Miwa S., Maddick M., Korolchuk V., Tsolou A., Gonos E.S., Thrasivoulou C., Saffrey M.J., Cameron K. (2012). Postmitotic neurons develop a p21-dependent senescence-like phenotype driven by a DNA damage response. Aging Cell.

[106] Farr J.N., Fraser D.G., Wang H., Jaehn K., Ogrodnik M.B., Weivoda M.M., Drake M.T., Tchkonia T., LeBrasseur N.K., Kirkland J.L. (2016). Identification of Senescent Cells in the Bone Microenvironment. J. Bone Miner. Res. Off. J. Am. Soc. Bone Miner. Res..

[107] Freund A., Laberge R.-M., Demaria M., Campisi J. (2012). Lamin B1 loss is a senescence-associated biomarker. Mol. Biol. Cell.

[108] da Silva Araujo G., Behm D.G., Monteiro E.R., de Melo Fiuza A., Gomes T.M., Vianna J.M., Reis M.S., da Silva Novaes J. (2019). Order Effects of Resistance and Stretching Exercises on Heart Rate Variability and Blood Pressure in Healthy Adults. J. Strength Cond. Res..

[109] Anderson R., Lagnado A., Maggiorani D., Walaszczyk A., Dookun E., Chapman J., Birch J., Salmonowicz H., Ogrodnik M., Jurk D. (2019). Length-independent telomere damage drives post-mitotic cardiomyocyte senescence. EMBO J..

[110] Herbig U., Ferreira M., Condel L., Carey D., Sedivy J.M. (2006). Cellular senescence in aging primates. Science.

[111] Chapman J., Fielder E., Passos J.F. (2019). Mitochondrial dysfunction and cell senescence: Deciphering a complex relationship. FEBS Lett..

[112] Kang C. (2019). Senolytics and Senostatics: A Two-Pronged Approach to Target Cellular Senescence for Delaying Aging and Age-Related Diseases. Mol. Cells.

[113] Kirkland J.L., Tchkonia T. (2017). Cellular Senescence: A Translational Perspective. EBioMedicine.

[114] Zhu Y., Tchkonia T., Pirtskhalava T., Gower A.C., Ding H., Giorgadze N., Palmer A.K., Ikeno Y., Hubbard G.B., Lenburg M. (2015). The Achilles’ heel of senescent cells: From transcriptome to senolytic drugs. Aging Cell.

[115] Khosla S., Farr J.N., Tchkonia T., Kirkland J.L. (2020). The role of cellular senescence in ageing and endocrine disease. Nat. Rev. Endocrinol..

[116] da Silva P., Ogrodnik M., Kucheryavenko O., Glibert J., Miwa S., Cameron K., Ishaq A., Saretzki G., Nagaraja-Grellscheid S., Nelson G. (2019). The bystander effect contributes to the accumulation of senescent cells in vivo. Aging Cell.

[117] Freund A., Orjalo A.V., Desprez P.-Y., Campisi J. (2010). Inflammatory networks during cellular senescence: Causes and consequences. Trends Mol. Med..

[118] Stout M.B., Tchkonia T., Pirtskhalava T., Palmer A.K., List E.O., Berryman D.E., Lubbers E.R., Escande C., Spong A., Masternak M.M. (2014). Growth hormone action predicts age-related white adipose tissue dysfunction and senescent cell burden in mice. Aging.

[119] del Nogal M., Troyano N., Calleros L., Griera M., Rodriguez-Puyol M., Rodriguez-Puyol D., Ruiz-Torres M.P. (2014). Hyperosmolarity induced by high glucose promotes senescence in human glomerular mesangial cells. Int. J. Biochem. Cell Biol..

[120] Li M., You L., Xue J., Lu Y. (2018). Ionizing Radiation-Induced Cellular Senescence in Normal, Non-transformed Cells and the Involved DNA Damage Response: A Mini Review. Front. Pharmacol..

[121] von Zglinicki T., Petrie J., Kirkwood T.B.L. (2003). Telomere-driven replicative senescence is a stress response. Nat. Biotechnol..

[122] da Silva P.F.L., Schumacher B. (2019). DNA damage responses in ageing. Open Biol..

[123] Andriani G.A., Almeida V.P., Faggioli F., Mauro M., Tsai W.L., Santambrogio L., Maslov A., Gadina M., Campisi J., Vijg J. (2016). Whole Chromosome Instability induces senescence and promotes SASP. Sci. Rep..

[124] Korolchuk V.I., Miwa S., Carroll B., von Zglinicki T. (2017). Mitochondria in Cell Senescence: Is Mitophagy the Weakest Link?. EBioMedicine.

[125] Serrano M., Lin A.W., McCurrach M.E., Beach D., Lowe S.W. (1997). Oncogenic ras Provokes Premature Cell Senescence Associated with Accumulation of p53 and p16INK4a. Cell.

[126] Ohtani N., Yamakoshi K., Takahashi A., Hara E. (2004). The p16INK4a-RB pathway: Molecular link between cellular senescence and tumor suppression. J. Med. Investig..

[127] Brown J.P., Wei W., Sedivy J.M. (1997). Bypass of senescence after disruption of p21CIP1/WAF1 gene in normal diploid human fibroblasts. Science.

[128] Vaiserman A.M., Lushchak O.V., Koliada A.K. (2016). Anti-aging pharmacology: Promises and pitfalls. Ageing Res. Rev..

[129] Cai Z., Zhang J., Li H. (2018). Selenium, aging and aging-related diseases. Aging Clin. Exp. Res..

[130] Zhang L., Zeng H., Cheng W.-H. (2018). Beneficial and paradoxical roles of selenium at nutritional levels of intake in healthspan and longevity. Free Radic. Biol. Med..

[131] Ferguson L.R., Karunasinghe N., Zhu S., Wang A.H. (2012). Selenium and its role in the maintenance of genomic stability. Mutat. Res..

[132] Yildiz A., Kaya Y., Tanriverdi O. (2019). Effect of the Interaction between Selenium and Zinc on DNA Repair in Association with Cancer Prevention. J. Cancer Prev..

[133] Wu J., Lyons G.H., Graham R.D., Fenech M.F. (2009). The effect of selenium, as selenomethionine, on genome stability and cytotoxicity in human lymphocytes measured using the cytokinesis-block micronucleus cytome assay. Mutagenesis.

[134] Singh N., Das M.K., Gautam R., Ramteke A., Rajamani P. (2019). Assessment of Intermittent Exposure of Zinc Oxide Nanoparticle (ZNP)-mediated Toxicity and Biochemical Alterations in the Splenocytes of Male Wistar Rat. Environ. Sci. Pollut. Res..

[135] Davies J. (1991). Specification and Proof in Real-Time Systems. Ph.D. Thesis.

[136] Fucassi F., Lowe J.E., Pavey K.D., Shah S., Faragher R.G., Green M.H., Paul F., O’Hare D., Cragg P.J. (2007). alpha-Lipoic acid and glutathione protect against the prooxidant activity of SOD/catalase mimetic manganese salen derivatives. J. Inorg. Biochem..

[137] Dogan S., Ozlem Elpek G., Kirimlioglu Konuk E., Demir N., Aslan M. (2012). Measurement of intracellular biomolecular oxidation in liver ischemia-reperfusion injury via immuno-spin trapping. Free Radic. Biol. Med..

[138] Martel J., Ojcius D.M., Ko Y.F., Ke P.Y., Wu C.Y., Peng H.H., Young J.D. (2019). Hormetic Effects of Phytochemicals on Health and Longevity. Trends Endocrinol. Metab..

[139] Erkekoglu P., Chao M.W., Tseng C.Y., Engelward B.P., Kose O., Kocer-Gumusel B., Wogan G.N., Tannenbaum S.R. (2019). Antioxidants and selenocompounds inhibit 3,5-dimethylaminophenol toxicity to human urothelial cells. Arch. Ind. Hyg. Toxicol..

[140] Verma P., Kunwar A., Indira Priyadarsini K. (2017). Effect of Low-Dose Selenium Supplementation on the Genotoxicity, Tissue Injury and Survival of Mice Exposed to Acute Whole-Body Irradiation. Biol. Trace Elem. Res..

[141] Tariba B., Živković T., Gajski G., Gerić M., Gluščić V., Garaj-Vrhovac V., Peraica M., Pizent A. (2017). In vitro effects of simultaneous exposure to platinum and cadmium on the activity of antioxidant enzymes and DNA damage and potential protective effects of selenium and zinc. Drug Chem. Toxicol..

[142] Li B., Li W., Tian Y., Guo S., Qian L., Xu D., Cao N. (2019). Selenium-Alleviated Hepatocyte Necrosis and DNA Damage in Cyclophosphamide-Treated Geese by Mitigating Oxidative Stress. Biol. Trace Elem. Res..

[143] Sadek K.M., Lebda M.A., Abouzed T.K., Nasr S.M., Shoukry M. (2017). Neuro- and nephrotoxicity of subchronic cadmium chloride exposure and the potential chemoprotective effects of selenium nanoparticles. Metab. Brain Dis..

[144] Gan F., Zhou Y., Hu Z., Hou L., Chen X., Xu S., Huang K. (2020). GPx1-mediated DNMT1 expression is involved in the blocking effects of selenium on OTA-induced cytotoxicity and DNA damage. Int. J. Biol. Macromol..

[145] Xu C., Qiao L., Ma L., Guo Y., Dou X., Yan S., Zhang B., Roman A. (2019). Biogenic selenium nanoparticles synthesized by Lactobacillus casei ATCC 393 alleviate intestinal epithelial barrier dysfunction caused by oxidative stress via Nrf2 signaling-mediated mitochondrial pathway. Int. J. Nanomed..

[146] Sengul E., Gelen V., Yildirim S., Tekin S., Dag Y. (2020). The Effects of Selenium in Acrylamide-Induced Nephrotoxicity in Rats: Roles of Oxidative Stress, Inflammation, Apoptosis, and DNA Damage. Biol. Trace Elem. Res..

[147] Ruggeri R.M., D’Ascola A., Vicchio T.M., Campo S., Gianì F., Giovinazzo S., Frasca F., Cannavò S., Campennì A., Trimarchi F. (2019). Selenium exerts protective effects against oxidative stress and cell damage in human thyrocytes and fibroblasts. Endocrine.

[148] Aravind P., Prasad M.N.V., Malec P., Waloszek A., Strzałka K. (2009). Zinc protects *Ceratophyllum demersum* L. (free-floating hydrophyte) against reactive oxygen species induced by cadmium. J. Trace Elem. Med. Biol. Organ Soc. Miner. Trace Elem..

[149] Emri E., Miko E., Bai P., Boros G., Nagy G., Rózsa D., Juhász T., Hegedűs C., Horkay I., Remenyik É. (2015). Effects of non-toxic zinc exposure on human epidermal keratinocytes. Metallomics.

[150] Sharif R., Thomas P., Zalewski P., Fenech M. (2015). Zinc supplementation influences genomic stability biomarkers, antioxidant activity, and zinc transporter genes in an elderly Australian population with low zinc status. Mol. Nutr. Food Res..

[151] Romualdo G.R., Goto R.L., Henrique Fernandes A.A., Cogliati B., Barbisan L.F. (2016). Dietary zinc deficiency predisposes mice to the development of preneoplastic lesions in chemically-induced hepatocarcinogenesis. Food Chem. Toxicol. Int. J. Publ. Br. Ind. Biol. Res. Assoc..

[152] Kaluza J., Madej D., Rusaczonek A., Siedlecka E., Pietruszka B. (2014). The effect of iron and zinc supplementation and its discontinuation on liver antioxidant status in rats fed deficient diets. Eur. J. Nutr..

[153] Brzóska M.M., Rogalska J. (2013). Protective effect of zinc supplementation against cadmium-induced oxidative stress and the RANK/RANKL/OPG system imbalance in the bone tissue of rats. Toxicol. Appl. Pharmacol..

[154] Maremanda K.P., Khan S., Jena G. (2014). Zinc protects cyclophosphamide-induced testicular damage in rat: Involvement of metallothionein, tesmin and Nrf2. Biochem. Biophys. Res. Commun..

[155] Sefi M., Chaâbane M., Elwej A., Bejaoui S., Marrekchi R., Jamoussi K., Gouiaa N., Boudawara Sellami T., El Cafsi M., Zeghal N. (2020). Zinc alleviates maneb-induced kidney injury in adult mice through modulation of oxidative stress, genotoxicity, and histopathological changes. Environ. Sci. Pollut. Res. Int..

[156] Piloni N.E., Caro A.A., Puntarulo S. (2018). Iron overload prevents oxidative damage to rat brain after chlorpromazine administration. Biometals Int. J. Role Met. Ions Biol. Biochem. Med..

[157] Díaz-Castro J., García Y., López-Aliaga I., Alférez M.J., Hijano S., Ramos A., Campos M.S. (2013). Influence of several sources and amounts of iron on DNA, lipid and protein oxidative damage during anaemia recovery. Biol. Trace Elem. Res..

[158] Gambaro R.C., Seoane A., Padula G. (2019). Oxidative Stress and Genomic Damage Induced In Vitro in Human Peripheral Blood by Two Preventive Treatments of Iron Deficiency Anemia. Biol. Trace Elem. Res..

[159] Chen K.L., Ven T.N., Crane M.M., Brunner M., Pun A.K., Helget K.L., Brower K., Chen D.E., Doan H., Dillard-Telm J.D. (2020). Loss of vacuolar acidity results in iron-sulfur cluster defects and divergent homeostatic responses during aging in Saccharomyces cerevisiae. Geroscience.

[160] Chen Y., Xiong S., Zhao F., Lu X., Wu B., Yang B. (2019). Effect of magnesium on reducing the UV-induced oxidative damage in marrow mesenchymal stem cells. J. Biomed. Mater. Res. Part A.

[161] Jiang W.D., Tang R.J., Liu Y., Wu P., Kuang S.Y., Jiang J., Tang L., Tang W.N., Zhang Y.A., Zhou X.Q. (2017). Impairment of gill structural integrity by manganese deficiency or excess related to induction of oxidative damage, apoptosis and dysfunction of the physical barrier as regulated by NF-κB, caspase and Nrf2 signaling in fish. Fish Shellfish Immunol..

[162] Zhu Y., Lu L., Liao X., Li W., Zhang L., Ji C., Lin X., Liu H.C., Odle J., Luo X. (2017). Maternal dietary manganese protects chick embryos against maternal heat stress via epigenetic-activated antioxidant and anti-apoptotic abilities. Oncotarget.

[163] Changizi V., Haeri S.A., Abbasi S., Rajabi Z., Mirdoraghi M. (2019). Radioprotective effects of vitamin A against gamma radiation in mouse bone marrow cells. MethodsX.

[164] Choudhry Q.N., Kim M.J., Kim T.G., Pan J.H., Kim J.H., Park S.J., Lee J.H., Kim Y.J. (2016). Saponin-Based Nanoemulsification Improves the Antioxidant Properties of Vitamin A and E in AML-12 Cells. Int. J. Mol. Sci..

[165] Wang G., Xiu P., Li F., Xin C., Li K. (2014). Vitamin A supplementation alleviates extrahepatic cholestasis liver injury through Nrf2 activation. Oxid. Med. Cell. Longev..

[166] Lehmann S., Loh S.H.Y., Martins L.M. (2017). Enhancing NAD salvage metabolism is neuroprotective in a PINK1 model of Parkinson’s disease. Biol. Open.

[167] Chhabra G., Garvey D.R., Singh C.K., Mintie C.A., Ahmad N. (2019). Effects and Mechanism of Nicotinamide Against UVA- and/or UVB-mediated DNA Damages in Normal Melanocytes. Photochem. Photobiol..

[168] Luo D., Peng Z., Yang L., Qu M., Xiong X., Xu L., Zhao X., Pan K., Ouyang K. (2019). Niacin Protects against Butyrate-Induced Apoptosis in Rumen Epithelial Cells. Oxid. Med. Cell. Longev..

[169] Endo N., Nishiyama K., Okabe M., Matsumoto M., Kanouchi H., Oka T. (2007). Vitamin B6 suppresses apoptosis of NM-1 bovine endothelial cells induced by homocysteine and copper. Biochim. Biophys. Acta.

[170] Abdou H.M., Wahby M.M. (2016). Neuroprotection of Grape Seed Extract and Pyridoxine against Triton-Induced Neurotoxicity. Oxid. Med. Cell. Longev..

[171] Merigliano C., Mascolo E., la Torre M., Saggio I., Vernì F. (2018). Protective role of vitamin B6 (PLP) against DNA damage in Drosophila models of type 2 diabetes. Sci. Rep..

[172] Ojeda M.L., Rua R.M., Nogales F., Díaz-Castro J., Murillo M.L., Carreras O. (2016). The Benefits of Administering Folic Acid in Order to Combat the Oxidative Damage Caused by Binge Drinking in Adolescent Rats. Alcohol Alcohol..

[173] Tu H.C., Lin M.Y., Lin C.Y., Hsiao T.H., Wen Z.H., Chen B.H., Fu T.F. (2019). Supplementation with 5-formyltetrahydrofolate alleviates ultraviolet B-inflicted oxidative damage in folate-deficient zebrafish. Ecotoxicol. Environ. Saf..

[174] Padmanabhan S., Waly M.I., Taranikanti V., Guizani N., Ali A., Rahman M.S., Al-Attabi Z., Al-Malky R.N., Al-Maskari S., Al-Ruqaishi B. (2019). Folate/Vitamin B12 Supplementation Combats Oxidative Stress-Associated Carcinogenesis in a Rat Model of Colon Cancer. Nutr. Cancer.

[175] Cui S., Lv X., Li W., Li Z., Liu H., Gao Y., Huang G. (2018). Folic acid modulates VPO1 DNA methylation levels and alleviates oxidative stress-induced apoptosis in vivo and in vitro. Redox Biol..

[176] Acharyya N., Deb B., Chattopadhyay S., Maiti S. (2015). Arsenic-Induced Antioxidant Depletion, Oxidative DNA Breakage, and Tissue Damages are Prevented by the Combined Action of Folate and Vitamin B12. Biol. Trace Elem. Res..

[177] Gómez-Meda B.C., Zamora-Perez A.L., Muñoz-Magallanes T., Sánchez-Parada M.G., García Bañuelos J.J., Guerrero-Velázquez C., Sánchez-Orozco L.V., Vera-Cruz J.M., Armendáriz-Borunda J., Zúñiga-González G.M. (2016). Nuclear abnormalities in buccal mucosa cells of patients with type I and II diabetes treated with folic acid. Mutat. Res. Genet. Toxicol. Environ. Mutagen..

[178] Baierle M., Göethel G., Nascimento S.N., Charão M.F., Moro A.M., Brucker N., Sauer E., Gauer B., Souto C., Durgante J. (2017). DNA damage in the elderly is associated with 5-MTHF levels: A pro-oxidant activity. Toxicol. Res..

[179] Boyacioglu M., Sekkin S., Kum C., Korkmaz D., Kiral F., Yalinkilinc H.S., Ak M.O., Akar F. (2014). The protective effects of vitamin C on the DNA damage, antioxidant defenses and aorta histopathology in chronic hyperhomocysteinemia induced rats. Exp. Toxicol. Pathol. Off. J. Ges. Toxikol. Pathol..

[180] Li Y., Zhang W., Chang L., Han Y., Sun L., Gong X., Tang H., Liu Z., Deng H., Ye Y. (2016). Vitamin C alleviates aging defects in a stem cell model for Werner syndrome. Protein Cell.

[181] Kawashima S., Funakoshi T., Sato Y., Saito N., Ohsawa H., Kurita K., Nagata K., Yoshida M., Ishigami A. (2018). Protective effect of pre- and post-vitamin C treatments on UVB-irradiation-induced skin damage. Sci. Rep..

[182] Johnson A.A., Naaldijk Y., Hohaus C., Meisel H.J., Krystel I., Stolzing A. (2016). Protective effects of alpha phenyl-tert-butyl nitrone and ascorbic acid in human adipose derived mesenchymal stem cells from differently aged donors. Aging.

[183] Gegotek A., Jarocka-Karpowicz I., Skrzydlewska E. (2019). Synergistic Cytoprotective Effects of Rutin and Ascorbic Acid on the Proteomic Profile of 3D-Cultured Keratinocytes Exposed to UVA or UVB Radiation. Nutrients.

[184] Alhusaini A.M., Faddah L.M., Hasan I.H., Jarallah S.J., Alghamdi S.H., Alhadab N.M., Badr A., Elorabi N., Zakaria E., Al-Anazi A. (2019). Vitamin C and Turmeric Attenuate Bax and Bcl-2 Proteins’ Expressions and DNA Damage in Lead Acetate-Induced Liver Injury. Dose Response.

[185] Halicka H.D., Zhao H., Li J., Lee Y.S., Hsieh T.C., Wu J.M., Darzynkiewicz Z. (2012). Potential anti-aging agents suppress the level of constitutive mTOR- and DNA damage-signaling. Aging.

[186] Chen L., Yang R., Qiao W., Zhang W., Chen J., Mao L., Goltzman D., Miao D. (2019). 1,25-Dihydroxyvitamin D exerts an antiaging role by activation of Nrf2-antioxidant signaling and inactivation of p16/p53-senescence signaling. Aging Cell.

[187] Chaiprasongsuk A., Janjetovic Z., Kim T.K., Jarrett S.G., D’Orazio J.A., Holick M.F., Tang E., Tuckey R.C., Panich U., Li W. (2019). Protective effects of novel derivatives of vitamin D and lumisterol against UVB-induced damage in human keratinocytes involve activation of Nrf2 and p53 defense mechanisms. Redox Biol..

[188] Siebert C., Dos Santos T.M., Bertó C.G., Parisi M.M., Coelho R.P., Manfredini V., Barbé-Tuana F.M., Wyse A. (2018). Vitamin D Supplementation Reverses DNA Damage and Telomeres Shortening Caused by Ovariectomy in Hippocampus of Wistar Rats. Neurotox. Res..

[189] Iqbal S., Khan S., Naseem I. (2018). Antioxidant Role of Vitamin D in Mice with Alloxan-Induced Diabetes. Can. J. Diabetes.

[190] Chang E. (2019). 1,25-Dihydroxyvitamin D Decreases Tertiary Butyl-Hydrogen Peroxide-Induced Oxidative Stress and Increases AMPK/SIRT1 Activation in C2C12 Muscle Cells. Molecules.

[191] Mehri N., Haddadi R., Ganji M., Shahidi S., Soleimani Asl S., Taheri Azandariani M., Ranjbar A. (2020). Effects of vitamin D in an animal model of Alzheimer’s disease: Behavioral assessment with biochemical investigation of Hippocampus and serum. Metab. Brain Dis..

[192] Qiao W., Yu S., Sun H., Chen L., Wang R., Wu X., Goltzman D., Miao D. (2020). 1,25-Dihydroxyvitamin D insufficiency accelerates age-related bone loss by increasing oxidative stress and cell senescence. Am. J. Transl. Res..

[193] Philips N., Samuel P., Keller T., Alharbi A., Alshalan S., Shamlan S.A. (2020). Beneficial Regulation of Cellular Oxidative Stress Effects, and Expression of Inflammatory, Angiogenic, and the Extracellular Matrix Remodeling Proteins by 1alpha,25-Dihydroxyvitamin D3 in a Melanoma Cell Line. Molecules.

[194] La Fata G., van Vliet N., Barnhoorn S., Brandt R., Etheve S., Chenal E., Grunenwald C., Seifert N., Weber P., Hoeijmakers J. (2017). Vitamin E Supplementation Reduces Cellular Loss in the Brain of a Premature Aging Mouse Model. J. Prev. Alzheimers Dis..

[195] Goon J.A., Nor Azman N.H.E., Abdul Ghani S.M., Hamid Z., Wan Ngah W.Z. (2017). Comparing palm oil tocotrienol rich fraction with α-tocopherol supplementation on oxidative stress in healthy older adults. Clin. Nutr. ESPEN.

[196] Bak M.J., Das Gupta S., Wahler J., Lee H.J., Li X., Lee M.J., Yang C.S., Suh N. (2017). Inhibitory Effects of γ- and δ-Tocopherols on Estrogen-Stimulated Breast Cancer in vitro and in vivo. Cancer Prev. Res..

[197] Aiub C.A.F., Pinto L.F.R., Felzenszwalb I. (2009). DNA-repair genes and vitamin E in the prevention of N-nitrosodiethylamine mutagenicity. Cell Biol. Toxicol..

[198] Taridi N.M., Abd Rani N., Abd Latiff A., Ngah W.Z.W., Mazlan M. (2014). Tocotrienol rich fraction reverses age-related deficits in spatial learning and memory in aged rats. Lipids.

[199] Ryan M.J., Dudash H.J., Docherty M., Geronilla K.B., Baker B.A., Haff G.G., Cutlip R.G., Alway S.E. (2010). Vitamin E and C supplementation reduces oxidative stress, improves antioxidant enzymes and positive muscle work in chronically loaded muscles of aged rats. Exp. Gerontol..

[200] Cooney R.V., Harwood P.J., Franke A.A., Narala K., Sundström A.K., Berggren P.O., Mordan L.J. (1995). Products of gamma-tocopherol reaction with NO2 and their formation in rat insulinoma (RINm5F) cells. Free Radic. Biol. Med..

[201] Chen J.X., Liu A., Lee M.J., Wang H., Yu S., Chi E., Reuhl K., Suh N., Yang C.S. (2017). δ- and γ-tocopherols inhibit phIP/DSS-induced colon carcinogenesis by protection against early cellular and DNA damages. Mol. Carcinog..

[202] Pu X., Wang Z., Zhou S., Klaunig J.E. (2016). Protective effects of antioxidants on acrylonitrile-induced oxidative stress in female F344 rats. Environ. Toxicol..

[203] Gao S., Qin T., Liu Z., Caceres M.A., Ronchi C.F., Chen C.Y., Yeum K.J., Taylor A., Blumberg J.B., Liu Y. (2011). Lutein and zeaxanthin supplementation reduces H_2_O_2_-induced oxidative damage in human lens epithelial cells. Mol. Vis..

[204] Baj A., Cedrowski J., Olchowik-Grabarek E., Ratkiewicz A., Witkowski S. (2019). Synthesis, DFT Calculations, and In Vitro Antioxidant Study on Novel Carba-Analogs of Vitamin E. Antioxidants.

[205] Moccia M., Capacchione A., Lanzillo R., Carbone F., Micillo T., Perna F., De Rosa A., Carotenuto A., Albero R., Matarese G. (2019). Coenzyme Q10 supplementation reduces peripheral oxidative stress and inflammation in interferon-β1a-treated multiple sclerosis. Ther. Adv. Neurol. Disord..

[206] Varela-López A., Ochoa J.J., Llamas-Elvira J.M., López-Frías M., Planells E., Ramirez-Tortosa M., Ramirez-Tortosa C.L., Giampieri F., Battino M., Quiles J.L. (2017). Age-Related Loss in Bone Mineral Density of Rats Fed Lifelong on a Fish Oil-Based Diet Is Avoided by Coenzyme Q Addition. Nutrients.

[207] Quiles J.L., Ochoa J.J., Battino M., Gutierrez-Rios P., Nepomuceno E.A., Frías M.L., Huertas J.R., Mataix J. (2005). Life-long supplementation with a low dosage of coenzyme Q10 in the rat: Effects on antioxidant status and DNA damage. BioFactors.

[208] Silvestri S., Orlando P., Armeni T., Padella L., Brugè F., Seddaiu G., Littarru G.P., Tiano L. (2015). Coenzyme Q10 and α-lipoic acid: Antioxidant and pro-oxidant effects in plasma and peripheral blood lymphocytes of supplemented subjects. J. Clin. Biochem. Nutr..

[209] Schniertshauer D., Müller S., Mayr T., Sonntag T., Gebhard D., Bergemann J. (2016). Accelerated Regeneration of ATP Level after Irradiation in Human Skin Fibroblasts by Coenzyme Q10. Photochem. Photobiol..

[210] Tarry-Adkins J.L., Blackmore H.L., Martin-Gronert M.S., Fernandez-Twinn D.S., McConnell J.M., Hargreaves I.P., Giussani D.A., Ozanne S.E. (2013). Coenzyme Q10 prevents accelerated cardiac aging in a rat model of poor maternal nutrition and accelerated postnatal growth. Mol. Metab..

[211] Carneiro M.F.H., Shin N., Karthikraj R., Barbosa F., Kannan K., Colaiacovo M.P. (2020). Antioxidant CoQ10 Restores Fertility by Rescuing Bisphenol A-Induced Oxidative DNA Damage in the *Caenorhabditis elegans* Germline. Genetics.

[212] Zhang M., ShiYang X., Zhang Y., Miao Y., Chen Y., Cui Z., Xiong B. (2019). Coenzyme Q10 ameliorates the quality of postovulatory aged oocytes by suppressing DNA damage and apoptosis. Free Radic. Biol. Med..

[213] Zhou C., Zhang X., Chen Y., Liu X., Sun Y., Xiong B. (2019). Glutathione alleviates the cadmium exposure-caused porcine oocyte meiotic defects via eliminating the excessive ROS. Environ. Pollut..

[214] Safaeipour M., Jauregui J., Castillo S., Bekarian M., Esparza D., Sanchez M., Stemp E. (2019). Glutathione Directly Intercepts DNA Radicals to Inhibit Oxidative DNA-Protein Cross-Linking Induced by the One-Electron Oxidation of Guanine. Biochemistry.

[215] Hagar H., Al Malki W. (2014). Betaine supplementation protects against renal injury induced by cadmium intoxication in rats: Role of oxidative stress and caspase-3. Environ. Toxicol. Pharmacol..

[216] Du Y., Peng J., Sun A., Tang Z., Ling W., Zhu H. (2009). Assessment of the effect of betaine on p16 and c-myc DNA methylation and mRNA expression in a chemical induced rat liver cancer model. BMC Cancer.

[217] Attia Y.A., El-Naggar A.S., Abou-Shehema B.M., Abdella A.A. (2019). Effect of Supplementation with Trimethylglycine (Betaine) and/or Vitamins on Semen Quality, Fertility, Antioxidant Status, DNA Repair and Welfare of Roosters Exposed to Chronic Heat Stress. Animals.

[218] Ansari F.A., Khan A.A., Mahmood R. (2019). Ameliorative effect of carnosine and *N*-acetylcysteine against sodium nitrite induced nephrotoxicity in rats. J. Cell. Biochem..

[219] Ansari F.A., Khan A.A., Mahmood R. (2018). Protective effect of carnosine and *N*-acetylcysteine against sodium nitrite-induced oxidative stress and DNA damage in rat intestine. Environ. Sci. Pollut. Res. Int..

[220] Kang J.H. (2009). Ferritin enhances salsolinol-mediated DNA strand breakage: Protection by carnosine and related compounds. Toxicol. Lett..

[221] Kang J.H. (2010). Protective effects of carnosine and homocarnosine on ferritin and hydrogen peroxide-mediated DNA damage. BMB Rep..

[222] Deng J., Zhong Y.F., Wu Y.P., Luo Z., Sun Y.M., Wang G.E., Kurihara H., Li Y.F., He R.R. (2018). Carnosine attenuates cyclophosphamide-induced bone marrow suppression by reducing oxidative DNA damage. Redox Biol..

[223] Hua X., Deng R., Li J., Chi W., Su Z., Lin J., Pflugfelder S.C., Li D.Q. (2015). Protective Effects of L-Carnitine against Oxidative Injury by Hyperosmolarity in Human Corneal Epithelial Cells. Invest. Ophthalmol. Vis. Sci..

[224] Thangasamy T., Jeyakumar P., Sittadjody S., Joyee A.G., Chinnakannu P. (2009). L-carnitine mediates protection against DNA damage in lymphocytes of aged rats. Biogerontology.

[225] Li J., Zhang Y., Luan H., Chen X., Han Y., Wang C. (2016). L-carnitine protects human hepatocytes from oxidative stress-induced toxicity through Akt-mediated activation of Nrf2 signaling pathway. Can. J. Physiol. Pharmacol..

[226] Salama S.A., Arab H.H., Omar H.A., Gad H.S., Abd-Allah G.M., Maghrabi I.A., Al Robaian M.M. (2018). L-carnitine mitigates UVA-induced skin tissue injury in rats through downregulation of oxidative stress, p38/c-Fos signaling, and the proinflammatory cytokines. Chem. Interact..

[227] Haripriya D., Sangeetha P., Kanchana A., Balu M., Panneerselvam C. (2005). Modulation of age-associated oxidative DNA damage in rat brain cerebral cortex, striatum and hippocampus by L-carnitine. Exp. Gerontol..

[228] Muthuswamy A.D., Vedagiri K., Ganesan M., Chinnakannu P. (2006). Oxidative stress-mediated macromolecular damage and dwindle in antioxidant status in aged rat brain regions: Role of L-carnitine and DL-alpha-lipoic acid. Clin. Chim. Acta Int. J. Clin. Chem..

[229] Juliet P.A.R., Joyee A.G., Jayaraman G., Mohankumar M.N., Panneerselvam C. (2005). Effect of L-carnitine on nucleic acid status of aged rat brain. Exp. Neurol..

[230] Ibrahim A.B., Mansour H.H., Shouman S.A., Eissa A.A., Abu El Nour S.M. (2014). Modulatory effects of L-carnitine on tamoxifen toxicity and oncolytic activity: In vivo study. Hum. Exp. Toxicol..

[231] Shadboorestan A., Shokrzadeh M., Ahangar N., Abdollahi M., Omidi M., Payam S.S.H. (2015). The chemoprotective effects of L-carnitine against genotoxicity induced by diazinon in rat blood lymphocyte. Toxicol. Ind. Health.

[232] Yu J., Ye J., Liu X., Han Y., Wang C. (2011). Protective effect of L-carnitine against H_2_O_2_-induced neurotoxicity in neuroblastoma (SH-SY5Y) cells. Neurol. Res..

[233] Jiang W.D., Feng L., Qu B., Wu P., Kuang S.Y., Jiang J., Tang L., Tang W.N., Zhang Y.A., Zhou X.Q. (2016). Changes in integrity of the gill during histidine deficiency or excess due to depression of cellular anti-oxidative ability, induction of apoptosis, inflammation and impair of cell-cell tight junctions related to Nrf2, TOR and NF-κB signaling in fish. Fish Shellfish Immunol..

[234] Ząbek-Adamska A., Drożdż R., Naskalski J.W. (2013). Dynamics of reactive oxygen species generation in the presence of copper (II)-histidine complex and cysteine. Acta Biochim. Pol..

[235] Marchetti D.P., Steffens L., Jacques C.E., Guerreiro G.B., Mescka C.P., Deon M., de Coelho D.M., Moura D.J., Viario A.G., Poletto F. (2018). Oxidative Imbalance, Nitrative Stress, and Inflammation in C6 Glial Cells Exposed to Hexacosanoic Acid: Protective Effect of *N*-acetyl-L-cysteine, Trolox, and Rosuvastatin. Cell. Mol. Neurobiol..

[236] Alam R.T., Imam T.S., Abo-Elmaaty A.M.A., Arisha A.H. (2019). Amelioration of fenitrothion induced oxidative DNA damage and inactivation of caspase-3 in the brain and spleen tissues of male rats by *N*-acetylcysteine. Life Sci..

[237] Yuan C., Wang L., Zhu L., Ran B., Xue X., Wang Z. (2019). *N*-acetylcysteine alleviated bisphenol A-induced testicular DNA hypermethylation of rare minnow (*Gobiocypris rarus*) by increasing cysteine contents. Ecotoxicol. Environ. Saf..

[238] Kim K.C., Ruwan Kumara M., Kang K.A., Piao M.J., Oh M.C., Ryu Y.S., Jo J.O., Mok Y.S., Shin J.H., Park Y. (2017). Exposure of keratinocytes to non-thermal dielectric barrier discharge plasma increases the level of 8-oxoguanine via inhibition of its repair enzyme. Mol. Med. Rep..

[239] Bigarella C.L., Li J., Rimmelé P., Liang R., Sobol R.W., Ghaffari S. (2017). FOXO3 Transcription Factor Is Essential for Protecting Hematopoietic Stem and Progenitor Cells from Oxidative DNA Damage. J. Biol. Chem..

[240] Jin J., Lv X., Chen L., Zhang W., Li J., Wang Q., Wang R., Lu X., Miao D. (2014). Bmi-1 plays a critical role in protection from renal tubulointerstitial injury by maintaining redox balance. Aging Cell.

[241] Yin Y., Xue X., Wang Q., Chen N., Miao D. (2016). Bmi1 plays an important role in dentin and mandible homeostasis by maintaining redox balance. Am. J. Transl. Res..

[242] Komoike Y., Matsuoka M. (2019). In vitro and in vivo studies of oxidative stress responses against acrylamide toxicity in zebrafish. J. Hazard. Mater..

[243] Shahat A.S., Hassan W.A., El-Sayed W.M. (2020). *N*-Acetylcysteine and Safranal prevented the brain damage induced by hyperthyroidism in adult male rats. Nutr. Neurosci..

[244] Ding Y.Y., Luan J.J., Fan Y., Olatunji O.J., Song J., Zuo J. (2020). Alpha-Mangostin reduced the viability of A594 cells in vitro by provoking ROS production through downregulation of NAMPT/NAD. Cell Stress Chaperon.

[245] Han Z., Xu Z., Chen L., Ye D., Yu Y., Zhang Y., Cao Y., Djibril B., Guo X., Gao X. (2020). Iron overload inhibits self-renewal of human pluripotent stem cells via DNA damage and generation of reactive oxygen species. FEBS Open Bio.

[246] Zhou X., Wang Z., Ni Y., Yu Y., Wang G., Chen L. (2020). Suppression effect of *N*-acetylcysteine on bone loss in ovariectomized mice. Am. J. Transl. Res..

[247] Chen L., Wang G., Wang Q., Liu Q., Sun Q. (2019). *N*-acetylcysteine prevents orchiectomy-induced osteoporosis by inhibiting oxidative stress and osteocyte senescence. Am. J. Transl. Res..

[248] Braidy N., Zarka M., Jugder B.E., Welch J., Jayasena T., Chan D., Sachdev P., Bridge W. (2019). The Precursor to Glutathione (GSH), γ-Glutamylcysteine (GGC), Can Ameliorate Oxidative Damage and Neuroinflammation Induced by Aβ_40_ Oligomers in Human Astrocytes. Front. Aging Neurosci..

[249] Acharyya N., Chattopadhyay S., Maiti S. (2014). Chemoprevention Against Arsenic-Induced Mutagenic DNA Breakage and Apoptotic Liver Damage in Rat Via Antioxidant and SOD1 Upregulation by Green Tea (*Camellia sinensis*) which Recovers Broken DNA Resulted from Arsenic-H_2_O_2_ Related In Vitro Oxidant Stress. J. Environ. Sci. Health Part C.

[250] Dickinson D., DeRossi S., Yu H., Thomas C., Kragor C., Paquin B., Hahn E., Ohno S., Yamamoto T., Hsu S. (2014). Epigallocatechin-3-gallate modulates anti-oxidant defense enzyme expression in murine submandibular and pancreatic exocrine gland cells and human HSG cells. Autoimmunity.

[251] Acharyya N., Sajed Ali S., Deb B., Chattopadhyay S., Maiti S. (2015). Green tea (*Camellia sinensis*) alleviates arsenic-induced damages to DNA and intestinal tissues in rat and in situ intestinal loop by reinforcing antioxidant system. Environ. Toxicol..

[252] Xu Y., Zhang J., Xiong L., Zhang L., Sun D., Liu H. (2010). Green tea polyphenols inhibit cognitive impairment induced by chronic cerebral hypoperfusion via modulating oxidative stress. J. Nutr. Biochem..

[253] Abraham S.K., Khandelwal N. (2013). Ascorbic acid and dietary polyphenol combinations protect against genotoxic damage induced in mice by endogenous nitrosation. Mutat. Res..

[254] Oršolić N., Sirovina D., Gajski G., Garaj-Vrhovac V., Jazvinšćak Jembrek M., Kosalec I. (2013). Assessment of DNA damage and lipid peroxidation in diabetic mice: Effects of propolis and epigallocatechin gallate (EGCG). Mutat. Res..

[255] Meng Q., Velalar C.N., Ruan R. (2008). Regulating the age-related oxidative damage, mitochondrial integrity, and antioxidative enzyme activity in Fischer 344 rats by supplementation of the antioxidant epigallocatechin-3-gallate. Rejuvenation Res..

[256] Pandır D. (2015). Protective effect of (−)-epigallocatechin-3-gallate on capsaicin-induced DNA damage and oxidative stress in human erythrocyes and leucocytes in vitro. Cytotechnology.

[257] López-Burillo S., Tan D.-X., Mayo J.C., Sainz R.M., Manchester L.C., Reiter R.J. (2003). Melatonin, xanthurenic acid, resveratrol, EGCG, vitamin C and alpha-lipoic acid differentially reduce oxidative DNA damage induced by Fenton reagents: A study of their individual and synergistic actions. J. Pineal Res..

[258] Shackelford R.E., Fu Y., Manuszak R.P., Brooks T.C., Sequeira A.P., Wang S., Lowery-Nordberg M., Chen A. (2006). Iron chelators reduce chromosomal breaks in ataxia-telangiectasia cells. DNA Repair.

[259] He Y., Tan D., Bai B., Wu Z., Ji S. (2017). Epigallocatechin-3-gallate attenuates acrylamide-induced apoptosis and astrogliosis in rat cerebral cortex. Toxicol. Mech. Methods.

[260] Othman A.I., Elkomy M.M., El-Missiry M.A., Dardor M. (2017). Epigallocatechin-3-gallate prevents cardiac apoptosis by modulating the intrinsic apoptotic pathway in isoproterenol-induced myocardial infarction. Eur. J. Pharmacol..

[261] Kaushal S., Ahsan A.U., Sharma V.L., Chopra M. (2019). Epigallocatechin gallate attenuates arsenic induced genotoxicity via regulation of oxidative stress in balb/C mice. Mol. Biol. Rep..

[262] Abib R.T., Quincozes-Santos A., Zanotto C., Zeidán-Chuliá F., Lunardi P.S., Gonçalves C.A., Gottfried C. (2010). Genoprotective effects of the green tea-derived polyphenol/epicatechin gallate in C6 astroglial cells. J. Med. Food.

[263] Huang C.C., Wu W.B., Fang J.Y., Chiang H.S., Chen S.K., Chen B.H., Chen Y.T., Hung C.F. (2007). (−)-Epicatechin-3-gallate, a green tea polyphenol is a potent agent against UVB-induced damage in HaCaT keratinocytes. Molecules.

[264] Yokozawa T., Rhyu D.Y., Cho E.J., Aoyagi K. (2003). Protective activity of (−)-epicatechin 3-O-gallate against peroxynitrite-mediated renal damage. Free Radic. Res..

[265] Anderson R.F., Fisher L.J., Hara Y., Harris T., Mak W.B., Melton L.D., Packer J.E. (2001). Green tea catechins partially protect DNA from (.)OH radical-induced strand breaks and base damage through fast chemical repair of DNA radicals. Carcinogenesis.

[266] Maheshwari N., Mahmood R. (2020). Protective effect of catechin on pentachlorophenol-induced cytotoxicity and genotoxicity in isolated human blood cells. Environ. Sci. Pollut. Res. Int..

[267] Unno K., Takabayashi F., Yoshida H., Choba D., Fukutomi R., Kikunaga N., Kishido T., Oku N., Hoshino M. (2007). Daily consumption of green tea catechin delays memory regression in aged mice. Biogerontology.

[268] Kishido T., Unno K., Yoshida H., Choba D., Fukutomi R., Asahina S., Iguchi K., Oku N., Hoshino M. (2007). Decline in glutathione peroxidase activity is a reason for brain senescence: Consumption of green tea catechin prevents the decline in its activity and protein oxidative damage in ageing mouse brain. Biogerontology.

[269] Delgado M.E., Haza A.I., García A., Morales P. (2009). Myricetin, quercetin, (+)-catechin and (−)-epicatechin protect against N-nitrosamines-induced DNA damage in human hepatoma cells. Toxicol. Vitr. Int. J. Publ. Assoc. BIBRA.

[270] Dauer A., Hensel A., Lhoste E., Knasmüller S., Mersch-Sundermann V. (2003). Genotoxic and antigenotoxic effects of catechin and tannins from the bark of *Hamamelis virginiana* L. in metabolically competent, human hepatoma cells (Hep G2) using single cell gel electrophoresis. Phytochemistry.

[271] Cheng Y.-T., Wu C.-H., Ho C.-Y., Yen G.-C. (2013). Catechin protects against ketoprofen-induced oxidative damage of the gastric mucosa by up-regulating Nrf2 in vitro and in vivo. J. Nutr. Biochem..

[272] Haza A.I., Morales P. (2011). Effects of (+)-catechin and (−)-epicatechin on heterocyclic amines-induced oxidative DNA damage. J. Appl. Toxicol..

[273] Charles C., Chemais M., Stévigny C., Dubois J., Nachergael A., Duez P. (2012). Measurement of the influence of flavonoids on DNA repair kinetics using the comet assay. Food Chem..

[274] Shimura T., Koyama M., Aono D., Kunugita N. (2019). Epicatechin as a promising agent to countermeasure radiation exposure by mitigating mitochondrial damage in human fibroblasts and mouse hematopoietic cells. FASEB J. Off. Publ. Fed. Am. Soc. Exp. Biol..

[275] Tvrda E., Straka P., Galbavy D., Ivanic P. (2019). Epicatechin Provides Antioxidant Protection to Bovine Spermatozoa Subjected to Induced Oxidative Stress. Molecules.

[276] Li J., Zheng J. (2019). Theaflavins prevent cartilage degeneration via AKT/FOXO3 signaling in vitro. Mol. Med. Rep..

[277] Han X., Zhang J., Xue X., Zhao Y., Lu L., Cui M., Miao W., Fan S. (2017). Theaflavin ameliorates ionizing radiation-induced hematopoietic injury via the NRF2 pathway. Free Radic. Biol. Med..

[278] Wang W., Sun Y., Liu J., Wang J., Li Y., Li H., Zhang W. (2012). Protective effect of theaflavins on homocysteine-induced injury in HUVEC cells in vitro. J. Cardiovasc. Pharmacol..

[279] Feng Q., Torii Y., Uchida K., Nakamura Y., Hara Y., Osawa T. (2002). Black tea polyphenols, theaflavins, prevent cellular DNA damage by inhibiting oxidative stress and suppressing cytochrome P450 1A1 in cell cultures. J. Agric. Food Chem..

[280] Sharma H., Kanwal R., Bhaskaran N., Gupta S. (2014). Plant flavone apigenin binds to nucleic acid bases and reduces oxidative DNA damage in prostate epithelial cells. PLoS ONE.

[281] Ahmad A., Zafar A., Ahmad M. (2019). Mitigating effects of apigenin on edifenphos-induced oxidative stress, DNA damage and apoptotic cell death in human peripheral blood lymphocytes. Food Chem. Toxicol. Int. J. Publ. Br. Ind. Biol. Res. Assoc..

[282] Wang E., Chen F., Hu X., Yuan Y. (2014). Protective effects of apigenin against furan-induced toxicity in mice. Food Funct..

[283] Wang N., Yi W.J., Tan L., Zhang J.H., Xu J., Chen Y., Qin M., Yu S., Guan J., Zhang R. (2017). Apigenin attenuates streptozotocin-induced pancreatic β cell damage by its protective effects on cellular antioxidant defense. Vitr. Cell. Dev. Biol. Anim..

[284] Ahmad A., Kumari P., Ahmad M. (2019). Apigenin attenuates edifenphos-induced toxicity by modulating ROS-mediated oxidative stress, mitochondrial dysfunction and caspase signal pathway in rat liver and kidney. Pestic. Biochem. Physiol..

[285] Alekhya Sita G.J., Gowthami M., Srikanth G., Krishna M.M., Rama Sireesha K., Sajjarao M., Nagarjuna K., Nagarjuna M., Chinnaboina G.K., Mishra A. (2019). Protective role of luteolin against bisphenol A-induced renal toxicity through suppressing oxidative stress, inflammation, and upregulating Nrf2/ARE/ HO-1 pathway. IUBMB Life.

[286] Rusak G., Piantanida I., Masić L., Kapuralin K., Durgo K., Kopjar N. (2010). Spectrophotometric analysis of flavonoid-DNA interactions and DNA damaging/protecting and cytotoxic potential of flavonoids in human peripheral blood lymphocytes. Chem. Interact..

[287] Wölfle U., Esser P.R., Simon-Haarhaus B., Martin S.F., Lademann J., Schempp C.M. (2011). UVB-induced DNA damage, generation of reactive oxygen species, and inflammation are effectively attenuated by the flavonoid luteolin in vitro and in vivo. Free. Radic. Biol. Med..

[288] Kim S., Chin Y.-W., Cho J. (2017). Protection of Cultured Cortical Neurons by Luteolin against Oxidative Damage through Inhibition of Apoptosis and Induction of Heme Oxygenase-1. Biol. Pharm. Bull..

[289] Melidou M., Riganakos K., Galaris D. (2005). Protection against nuclear DNA damage offered by flavonoids in cells exposed to hydrogen peroxide: The role of iron chelation. Free Radic. Biol. Med..

[290] Manzolli E.S., Serpeloni J.M., Grotto D., Bastos J.K., Antunes L.M., Barbosa Junior F., Barcelos G.R. (2015). Protective effects of the flavonoid chrysin against methylmercury-induced genotoxicity and alterations of antioxidant status, in vivo. Oxid. Med. Cell. Longev..

[291] Sultana S., Verma K., Khan R. (2012). Nephroprotective efficacy of chrysin against cisplatin-induced toxicity via attenuation of oxidative stress. J. Pharm. Pharmacol..

[292] Sassi A., Boubaker J., Loussaief A., Jomaa K., Ghedira K., Chekir-Ghedira L. (2020). Protective Effect of Chrysin, a Dietary Flavone against Genotoxic and Oxidative Damage Induced by Mitomycin C in Balb/C Mice. Nutr. Cancer.

[293] Liu X., Zhang R., Shi H., Li X., Li Y., Taha A., Xu C. (2018). Protective effect of curcumin against ultraviolet A irradiation-induced photoaging in human dermal fibroblasts. Mol. Med. Rep..

[294] Iqbal M., Okazaki Y., Okada S. (2009). Curcumin attenuates oxidative damage in animals treated with a renal carcinogen, ferric nitrilotriacetate (Fe-NTA): Implications for cancer prevention. Mol. Cell. Biochem..

[295] Biswas J., Sinha D., Mukherjee S., Roy S., Siddiqi M., Roy M. (2010). Curcumin protects DNA damage in a chronically arsenic-exposed population of West Bengal. Hum. Exp. Toxicol..

[296] Chan W., Wu H. (2006). Protective effects of curcumin on methylglyoxal-induced oxidative DNA damage and cell injury in human mononuclear cells. Acta Pharmacol. Sin..

[297] Ciftci G., Aksoy A., Cenesiz S., Sogut M.U., Yarim G.F., Nisbet C., Guvenc D., Ertekin A. (2015). Therapeutic role of curcumin in oxidative DNA damage caused by formaldehyde. Microsc. Res. Tech..

[298] Eke D., Çelik A. (2016). Curcumin prevents perfluorooctane sulfonate-induced genotoxicity and oxidative DNA damage in rat peripheral blood. Drug Chem. Toxicol..

[299] Sarkar B., Dhiman M., Mittal S., Mantha A.K. (2017). Curcumin revitalizes Amyloid beta (25-35)-induced and organophosphate pesticides pestered neurotoxicity in SH-SY5Y and IMR-32 cells via activation of APE1 and Nrf2. Metab. Brain Dis..

[300] Li H., Gao A., Jiang N., Liu Q., Liang B., Li R., Zhang E., Li Z., Zhu H. (2016). Protective Effect of Curcumin Against Acute Ultraviolet B Irradiation-induced Photo-damage. Photochem. Photobiol..

[301] Mladenović M., Matić S., Stanić S., Solujić S., Mihailović V., Stanković N., Katanić J. (2013). Combining molecular docking and 3-D pharmacophore generation to enclose the in vivo antigenotoxic activity of naturally occurring aromatic compounds: Myricetin, quercetin, rutin, and rosmarinic acid. Biochem. Pharmacol..

[302] Alugoju P., Periyasamy L., Dyavaiah M. (2018). Quercetin enhances stress resistance in mutant cells to different stressors. J. Food Sci. Technol..

[303] Pietsch K., Saul N., Chakrabarti S., Stürzenbaum S.R., Menzel R., Steinberg C.E.W. (2011). Hormetins, antioxidants and prooxidants: Defining quercetin-, caffeic acid- and rosmarinic acid-mediated life extension in C. elegans. Biogerontology.

[304] Yin Y., Li W., Son Y.O., Sun L., Lu J., Kim D., Wang X., Yao H., Wang L., Pratheeshkumar P. (2013). Quercitrin protects skin from UVB-induced oxidative damage. Toxicol. Appl. Pharmacol..

[305] Wilms L.C., Hollman P.C.H., Boots A.W., Kleinjans J.C.S. (2005). Protection by quercetin and quercetin-rich fruit juice against induction of oxidative DNA damage and formation of BPDE-DNA adducts in human lymphocytes. Mutat. Res..

[306] Alam M.M., Meerza D., Naseem I. (2014). Protective effect of quercetin on hyperglycemia, oxidative stress and DNA damage in alloxan induced type 2 diabetic mice. Life Sci..

[307] Barcelos G.R., Grotto D., Serpeloni J.M., Angeli J.P., Rocha B.A., de Oliveira Souza V.C., Vicentini J.T., Emanuelli T., Bastos J.K., Antunes L.M. (2011). Protective properties of quercetin against DNA damage and oxidative stress induced by methylmercury in rats. Arch. Toxicol..

[308] Cao L., Tan C., Meng F., Liu P., Reece E.A., Zhao Z. (2016). Amelioration of intracellular stress and reduction of neural tube defects in embryos of diabetic mice by phytochemical quercetin. Sci. Rep..

[309] Chaiprasongsuk A., Onkoksoong T., Pluemsamran T., Limsaengurai S., Panich U. (2016). Photoprotection by dietary phenolics against melanogenesis induced by UVA through Nrf2-dependent antioxidant responses. Redox Biol..

[310] Soberón J.R., Sgariglia M.A., Sampietro D.A., Quiroga E.N., Vattuone M.A. (2010). Free radical scavenging activities and inhibition of inflammatory enzymes of phenolics isolated from Tripodanthus acutifolius. J. Ethnopharmacol..

[311] del Carmen García-Rodríguez M., Nicolás-Méndez T., Montaño-Rodríguez A.R., Altamirano-Lozano M.A. (2014). Antigenotoxic Effects of (−)-Epigallocatechin-3-Gallate (EGCG), Quercetin, and Rutin on Chromium Trioxide-Induced Micronuclei in the Polychromatic Erythrocytes of Mouse Peripheral Blood. J. Toxicol. Environ. Health Part A.

[312] Han X., Xue X., Zhao Y., Li Y., Liu W., Zhang J., Fan S. (2017). Rutin-Enriched Extract from *Coriandrum sativum* L. Ameliorates Ionizing Radiation-Induced Hematopoietic Injury. Int. J. Mol. Sci..

[313] Umarani V., Muvvala S., Ramesh A., Lakshmi B.V.S., Sravanthi N. (2015). Rutin potentially attenuates fluoride-induced oxidative stress-mediated cardiotoxicity, blood toxicity and dyslipidemia in rats. Toxicol. Mech. Methods.

[314] Al-Rejaie S.S., Aleisa A.M., Sayed-Ahmed M.M., Al-Shabanah O.A., Abuohashish H.M., Ahmed M.M., Al-Hosaini K.A., Hafez M.M. (2013). Protective effect of rutin on the antioxidant genes expression in hypercholestrolemic male Westar rat. BMC Complement. Altern. Med..

[315] Khan R.A., Khan M.R., Sahreen S. (2012). Protective effects of rutin against potassium bromate induced nephrotoxicity in rats. BMC Complement. Altern. Med..

[316] Khan R.A., Khan M.R., Sahreen S. (2012). CCl4-induced hepatotoxicity: Protective effect of rutin on p53, CYP2E1 and the antioxidative status in rat. BMC Complement. Altern. Med..

[317] Li R., Yuan C., Dong C., Shuang S., Choi M.M.F. (2011). In vivo antioxidative effect of isoquercitrin on cadmium-induced oxidative damage to mouse liver and kidney. Naunyn Schmiedebergs Arch. Pharmacol..

[318] Li H.B., Yi X., Gao J.M., Ying X.X., Guan H.Q., Li J.C. (2008). The mechanism of hyperoside protection of ECV-304 cells against tert-butyl hydroperoxide-induced injury. Pharmacology.

[319] Piao M.J., Kang K.A., Zhang R., Ko D.O., Wang Z.H., You H.J., Kim H.S., Kim J.S., Kang S.S., Hyun J.W. (2008). Hyperoside prevents oxidative damage induced by hydrogen peroxide in lung fibroblast cells via an antioxidant effect. Biochim. Biophys. Acta.

[320] Tsai M.S., Wang Y.H., Lai Y.Y., Tsou H.K., Liou G.G., Ko J.L., Wang S.H. (2018). Kaempferol protects against propacetamol-induced acute liver injury through CYP2E1 inactivation, UGT1A1 activation, and attenuation of oxidative stress, inflammation and apoptosis in mice. Toxicol. Lett..

[321] Kumar A.D.N., Bevara G.B., Kaja L.K., Badana A.K., Malla R.R. (2016). Protective effect of 3-O-methyl quercetin and kaempferol from Semecarpus anacardium against HO induced cytotoxicity in lung and liver cells. BMC Complement. Altern. Med..

[322] Al Sabaani N. (2020). Kaempferol Protects Against Hydrogen Peroxide-Induced Retinal Pigment Epithelium Cell Inflammation and Apoptosis by Activation of SIRT1 and Inhibition of PARP1. J. Ocul. Pharmacol. Ther..

[323] Chen W., Li Y., Li J., Han Q., Ye L., Li A. (2011). Myricetin affords protection against peroxynitrite-mediated DNA damage and hydroxyl radical formation. Food Chem. Toxicol. Int. J. Publ. Br. Ind. Biol. Res. Assoc..

[324] Huang J.H., Huang C.C., Fang J.Y., Yang C., Chan C.M., Wu N.L., Kang S.W., Hung C.F. (2010). Protective effects of myricetin against ultraviolet-B-induced damage in human keratinocytes. Toxicol. Vitr. Int. J. Publ. Assoc. BIBRA.

[325] Akhtar S., Najafzadeh M., Isreb M., Newton L., Gopalan R.C., Anderson D. (2020). ROS-induced oxidative damage in lymphocytes ex vivo/in vitro from healthy individuals and MGUS patients: Protection by myricetin bulk and nanoforms. Arch. Toxicol..

[326] Lee M.H., Cha H.J., Choi E.O., Han M.H., Kim S.O., Kim G.Y., Hong S.H., Park C., Moon S.K., Jeong S.J. (2017). Antioxidant and cytoprotective effects of morin against hydrogen peroxide-induced oxidative stress are associated with the induction of Nrf-2-mediated HO-1 expression in V79-4 Chinese hamster lung fibroblasts. Int. J. Mol. Med..

[327] Veerappan I., Sankareswaran S.K., Palanisamy R. (2019). Morin Protects Human Respiratory Cells from PM Induced Genotoxicity by Mitigating ROS and Reverting Altered miRNA Expression. Int. J. Environ. Res. Public Health.

[328] Vanitha P., Senthilkumar S., Dornadula S., Anandhakumar S., Rajaguru P., Ramkumar K.M. (2017). Morin activates the Nrf2-ARE pathway and reduces oxidative stress-induced DNA damage in pancreatic beta cells. Eur. J. Pharmacol..

[329] Komirishetty P., Areti A., Sistla R., Kumar A. (2016). Morin Mitigates Chronic Constriction Injury (CCI)-Induced Peripheral Neuropathy by Inhibiting Oxidative Stress Induced PARP Over-Activation and Neuroinflammation. Neurochem. Res..

[330] Kapoor R., Kakkar P. (2012). Protective role of morin, a flavonoid, against high glucose induced oxidative stress mediated apoptosis in primary rat hepatocytes. PLoS ONE.

[331] Zhang R., Kang K.A., Kang S.S., Park J.W., Hyun J.W. (2011). Morin (2′,3,4′,5,7-pentahydroxyflavone) protected cells against γ-radiation-induced oxidative stress. Basic Clin. Pharmacol. Toxicol..

[332] Verdan A.M., Wang H.C., García C.R., Henry W.P., Brumaghim J.L. (2011). Iron binding of 3-hydroxychromone, 5-hydroxychromone, and sulfonated morin: Implications for the antioxidant activity of flavonols with competing metal binding sites. J. Inorg. Biochem..

[333] Wang T., Lin H., Tu Q., Liu J., Li X. (2016). Fisetin Protects DNA against Oxidative Damage and Its Possible Mechanism. Adv. Pharm. Bull..

[334] Piao M.J., Kim K.C., Chae S., Keum Y.S., Kim H.S., Hyun J.W. (2013). Protective Effect of Fisetin (3,7,3′,4′-Tetrahydroxyflavone) against γ-Irradiation-Induced Oxidative Stress and Cell Damage. Biomol. Ther..

[335] Kang K.A., Piao M.J., Kim K.C., Cha J.W., Zheng J., Yao C.W., Chae S., Hyun J.W. (2014). Fisetin attenuates hydrogen peroxide-induced cell damage by scavenging reactive oxygen species and activating protective functions of cellular glutathione system. Vitr. Cell. Dev. Biol. Anim..

[336] Rodius S., de Klein N., Jeanty C., Sánchez-Iranzo H., Crespo I., Ibberson M., Xenarios I., Dittmar G., Mercader N., Niclou S.P. (2020). Fisetin protects against cardiac cell death through reduction of ROS production and caspases activity. Sci. Rep..

[337] Ganaie M.A., Jan B.L., Khan T.H., Alharthy K.M., Sheikh I.A. (2019). The Protective Effect of Naringenin on Oxaliplatin-Induced Genotoxicity in Mice. Chem. Pharm. Bull..

[338] Motawi T.K., Teleb Z.A., El-Boghdady N.A., Ibrahim S.A. (2014). Effect of simvastatin and naringenin coadministration on rat liver DNA fragmentation and cytochrome P450 activity: An in vivo and in vitro study. J. Physiol. Biochem..

[339] Chtourou Y., Slima A.B., Makni M., Gdoura R., Fetoui H. (2015). Naringenin protects cardiac hypercholesterolemia-induced oxidative stress and subsequent necroptosis in rats. Pharmacol. Rep..

[340] Roy A., Das A., Das R., Haldar S., Bhattacharya S., Haldar P.K. (2014). Naringenin, a citrus flavonoid, ameliorates arsenic-induced toxicity in Swiss albino mice. J. Environ. Pathol. Toxicol. Oncol. Off. Organ Int. Soc. Environ. Toxicol. Cancer.

[341] Kapoor R., Rizvi F., Kakkar P. (2013). Naringenin prevents high glucose-induced mitochondria-mediated apoptosis involving AIF, Endo-G and caspases. Apoptosis Int. J. Program. Cell Death.

[342] Manna K., Das U., Das D., Kesh S.B., Khan A., Chakraborty A., Dey S. (2015). Naringin inhibits gamma radiation-induced oxidative DNA damage and inflammation, by modulating p53 and NF-κB signaling pathways in murine splenocytes. Free Radic. Res..

[343] Kumar V.S., Rajmane A.R., Adil M., Kandhare A.D., Ghosh P., Bodhankar S.L. (2014). Naringin ameliorates acetic acid induced colitis through modulation of endogenous oxido-nitrosative balance and DNA damage in rats. J. Biomed. Res..

[344] Jagetia G.C., Reddy T.K. (2011). Alleviation of iron induced oxidative stress by the grape fruit flavanone naringin in vitro. Chem. Interact..

[345] NilamberLal Das R., Muruhan S., Nagarajan R.P., Balupillai A. (2019). Naringin prevents ultraviolet-B radiation-induced oxidative damage and inflammation through activation of peroxisome proliferator-activated receptor γ in mouse embryonic fibroblast (NIH-3T3) cells. J. Biochem. Mol. Toxicol..

[346] Caglayan C., Temel Y., Kandemir F.M., Yildirim S., Kucukler S. (2018). Naringin protects against cyclophosphamide-induced hepatotoxicity and nephrotoxicity through modulation of oxidative stress, inflammation, apoptosis, autophagy, and DNA damage. Environ. Sci. Pollut. Res. Int..

[347] Lim Y.J., Kim J.H., Pan J.H., Kim J.K., Park T.S., Kim Y.J., Lee J.H., Kim J.H. (2018). Naringin Protects Pancreatic β-Cells against Oxidative Stress-Induced Apoptosis by Inhibiting Both Intrinsic and Extrinsic Pathways in Insulin-Deficient Diabetic Mice. Mol. Nutr. Food Res..

[348] Samie A., Sedaghat R., Baluchnejadmojarad T., Roghani M. (2018). Hesperetin, a citrus flavonoid, attenuates testicular damage in diabetic rats via inhibition of oxidative stress, inflammation, and apoptosis. Life Sci..

[349] Turk E., Kandemir F.M., Yildirim S., Caglayan C., Kucukler S., Kuzu M. (2019). Protective Effect of Hesperidin on Sodium Arsenite-Induced Nephrotoxicity and Hepatotoxicity in Rats. Biol. Trace Elem. Res..

[350] Homayouni F., Haidari F., Hedayati M., Zakerkish M., Ahmadi K. (2017). Hesperidin Supplementation Alleviates Oxidative DNA Damage and Lipid Peroxidation in Type 2 Diabetes: A Randomized Double-Blind Placebo-Controlled Clinical Trial. Phytother. Res..

[351] Sahu B.D., Kuncha M., Sindhura G.J., Sistla R. (2013). Hesperidin attenuates cisplatin-induced acute renal injury by decreasing oxidative stress, inflammation and DNA damage. Phytomed. Int. J. Phytother. Phytopharm..

[352] Trivedi P.P., Kushwaha S., Tripathi D.N., Jena G.B. (2011). Cardioprotective effects of hesperetin against doxorubicin-induced oxidative stress and DNA damage in rat. Cardiovasc. Toxicol..

[353] Kalpana K.B., Devipriya N., Srinivasan M., Vishwanathan P., Thayalan K., Menon V.P. (2011). Evaluating the radioprotective effect of hesperidin in the liver of Swiss albino mice. Eur. J. Pharmacol..

[354] Elhelaly A.E., AlBasher G., Alfarraj S., Almeer R., Bahbah E.I., Fouda M., Bungău S.G., Aleya L., Abdel-Daim M.M. (2019). Protective effects of hesperidin and diosmin against acrylamide-induced liver, kidney, and brain oxidative damage in rats. Environ. Sci. Pollut. Res. Int..

[355] Mahgoub S., Sallam A.O., Sarhan H.K.A., Ammar A.A.A., Soror S.H. (2020). Role of Diosmin in protection against the oxidative stress induced damage by gamma-radiation in Wistar albino rats. Regul. Toxicol. Pharmacol..

[356] Rehman M.U., Tahir M., Quaiyoom Khan A., Khan R., Lateef A., Hamiza O.O., Ali F., Sultana S. (2013). Diosmin protects against trichloroethylene-induced renal injury in Wistar rats: Plausible role of p53, Bax and caspases. Br. J. Nutr..

[357] Jindal R., Sinha R., Brar P. (2019). Evaluating the protective efficacy of Silybum marianum against deltamethrin induced hepatotoxicity in piscine model. Environ. Toxicol. Pharmacol..

[358] Fu H., Lin M., Katsumura Y., Yokoya A., Hata K., Muroya Y., Fujii K., Shikazono N. (2010). Protective effects of silybin and analogues against X-ray radiation-induced damage. Acta Biochim. Biophys. Sin..

[359] Muthumani M., Prabu S.M. (2012). Silibinin potentially protects arsenic-induced oxidative hepatic dysfunction in rats. Toxicol. Mech. Methods.

[360] Rajnochová Svobodová A., Gabrielová E., Michaelides L., Kosina P., Ryšavá A., Ulrichová J., Zálešák B., Vostálová J. (2018). UVA-photoprotective potential of silymarin and silybin. Arch. Dermatol. Res..

[361] Marrazzo G., Bosco P., La Delia F., Scapagnini G., Di Giacomo C., Malaguarnera M., Galvano F., Nicolosi A., Li Volti G. (2011). Neuroprotective effect of silibinin in diabetic mice. Neurosci. Lett..

[362] Sozen H., Celik O.I., Cetin E.S., Yilmaz N., Aksozek A., Topal Y., Cigerci I.H., Beydilli H. (2015). Evaluation of the protective effect of silibinin in rats with liver damage caused by itraconazole. Cell Biochem. Biophys..

[363] Vacek J., Zatloukalová M., Desmier T., Nezhodová V., Hrbáč J., Kubala M., Křen V., Ulrichová J., Trouillas P. (2013). Antioxidant, metal-binding and DNA-damaging properties of flavonolignans: A joint experimental and computational highlight based on 7-O-galloylsilybin. Chem. Interact..

[364] Essid E., Dernawi Y., Petzinger E. (2012). Apoptosis induction by OTA and TNF-α in cultured primary rat hepatocytes and prevention by silibinin. Toxins.

[365] Ghosh S., Sarkar A., Bhattacharyya S., Sil P.C. (2016). Silymarin Protects Mouse Liver and Kidney from Thioacetamide Induced Toxicity by Scavenging Reactive Oxygen Species and Activating PI3K-Akt Pathway. Front. Pharmacol..

[366] Russo A., Cardile V., Lombardo L., Vanella L., Acquaviva R. (2006). Genistin inhibits UV light-induced plasmid DNA damage and cell growth in human melanoma cells. J. Nutr. Biochem..

[367] Wei H., Ca Q., Rahn R., Zhang X., Wang Y., Lebwohl M. (1998). DNA structural integrity and base composition affect ultraviolet light-induced oxidative DNA damage. Biochemistry.

[368] Wu H.-J., Chan W.-H. (2007). Genistein protects methylglyoxal-induced oxidative DNA damage and cell injury in human mononuclear cells. Toxicol. Vitr. Int. J. Publ. Assoc. BIBRA.

[369] Terra V.A., Souza-Neto F.P., Frade M.A., Ramalho L.N., Andrade T.A., Pasta A.A., Conchon A.C., Guedes F.A., Luiz R.C., Cecchini R. (2015). Genistein prevents ultraviolet B radiation-induced nitrosative skin injury and promotes cell proliferation. J. Photochem. Photobiol. B Biol..

[370] Wang R., Tu J., Zhang Q., Zhang X., Zhu Y., Ma W., Cheng C., Brann D.W., Yang F. (2013). Genistein attenuates ischemic oxidative damage and behavioral deficits via eNOS/Nrf2/HO-1 signaling. Hippocampus.

[371] Yen G.-C., Lai H.-H. (2002). Inhibitory effects of isoflavones on nitric oxide- or peroxynitrite-mediated DNA damage in RAW 264.7 cells and phiX174 DNA. Food Chem. Toxicol. Int. J. Publ. Br. Ind. Biol. Res. Assoc..

[372] Leung H.Y., Yung L.H., Poon C.H., Shi G., Lu A.-L., Leung L.K. (2009). Genistein protects against polycyclic aromatic hydrocarbon-induced oxidative DNA damage in non-cancerous breast cells MCF-10A. Br. J. Nutr..

[373] Raschke M., Rowland I.R., Magee P.J., Pool-Zobel B.L. (2006). Genistein protects prostate cells against hydrogen peroxide-induced DNA damage and induces expression of genes involved in the defence against oxidative stress. Carcinogenesis.

[374] Erba D., Casiraghi M.C., Martinez-Conesa C., Goi G., Massaccesi L. (2012). Isoflavone supplementation reduces DNA oxidative damage and increases O-β-N-acetyl-D-glucosaminidase activity in healthy women. Nutr. Res..

[375] Toyoizumi T., Sekiguchi H., Takabayashi F., Deguchi Y., Masuda S., Kinae N. (2010). Induction effect of coadministration of soybean isoflavones and sodium nitrite on DNA damage in mouse stomach. Food Chem. Toxicol. Int. J. Publ. Br. Ind. Biol. Res. Assoc..

[376] Chen M., Samuel V.P., Wu Y., Dang M., Lin Y., Sriramaneni R., Sah S.K., Chinnaboina G.K., Zhang G. (2019). Nrf2/HO-1 Mediated Protective Activity of Genistein Against Doxorubicin-Induced Cardiac Toxicity. J. Environ. Pathol. Toxicol. Oncol..

[377] Miltonprabu S., Nazimabashir, Manoharan V. (2016). Hepatoprotective effect of grape seed proanthocyanidins on Cadmium-induced hepatic injury in rats: Possible involvement of mitochondrial dysfunction, inflammation and apoptosis. Toxicol. Rep..

[378] Bashir N., Shagirtha K., Manoharan V., Miltonprabu S. (2019). The molecular and biochemical insight view of grape seed proanthocyanidins in ameliorating cadmium-induced testes-toxicity in rat model: Implication of PI3K/Akt/Nrf-2 signaling. Biosci. Rep..

[379] Sharma S.D., Meeran S.M., Katiyar S.K. (2007). Dietary grape seed proanthocyanidins inhibit UVB-induced oxidative stress and activation of mitogen-activated protein kinases and nuclear factor-kappaB signaling in in vivo SKH-1 hairless mice. Mol. Cancer Ther..

[380] Mantena S.K., Katiyar S.K. (2006). Grape seed proanthocyanidins inhibit UV-radiation-induced oxidative stress and activation of MAPK and NF-kappaB signaling in human epidermal keratinocytes. Free Radic. Biol. Med..

[381] Niu L., Shao M., Liu Y., Hu J., Li R., Xie H., Zhou L., Shi L., Zhang R., Niu Y. (2017). Reduction of oxidative damages induced by titanium dioxide nanoparticles correlates with induction of the Nrf2 pathway by GSPE supplementation in mice. Chem. Interact..

[382] Liu B., Jiang H., Lu J., Baiyun R., Li S., Lv Y., Li D., Wu H., Zhang Z. (2018). Grape seed procyanidin extract ameliorates lead-induced liver injury via miRNA153 and AKT/GSK-3β/Fyn-mediated Nrf2 activation. J. Nutr. Biochem..

[383] Thilakarathna W.P.D.W., Rupasinghe H.P.V. (2019). Microbial metabolites of proanthocyanidins reduce chemical carcinogen-induced DNA damage in human lung epithelial and fetal hepatic cells in vitro. Food Chem. Toxicol. Int. J. Publ. Br. Ind. Biol. Res. Assoc..

[384] Suantawee T., Cheng H., Adisakwattana S. (2016). Protective effect of cyanidin against glucose- and methylglyoxal-induced protein glycation and oxidative DNA damage. Int. J. Biol. Macromol..

[385] Wang Y., Fu X.T., Li D.W., Wang K., Wang X.Z., Li Y., Sun B.L., Yang X.Y., Zheng Z.C., Cho N.C. (2016). Cyanidin suppresses amyloid beta-induced neurotoxicity by inhibiting reactive oxygen species-mediated DNA damage and apoptosis in PC12 cells. Neural Regen. Res..

[386] Li D.W., Sun J.Y., Wang K., Zhang S., Hou Y.J., Yang M.F., Fu X.Y., Zhang Z.Y., Mao L.L., Yuan H. (2015). Attenuation of Cisplatin-Induced Neurotoxicity by Cyanidin, a Natural Inhibitor of ROS-Mediated Apoptosis in PC12 Cells. Cell. Mol. Neurobiol..

[387] Khandelwal N., Abraham S.K. (2014). Intake of anthocyanidins pelargonidin and cyanidin reduces genotoxic stress in mice induced by diepoxybutane, urethane and endogenous nitrosation. Environ. Toxicol. Pharmacol..

[388] Zhang C., Guo X., Cai W., Ma Y., Zhao X. (2015). Binding characteristics and protective capacity of cyanidin-3-glucoside and its aglycon to calf thymus DNA. J. Food Sci..

[389] Hu Y., Ma Y., Wu S., Chen T., He Y., Sun J., Jiao R., Jiang X., Huang Y., Deng L. (2016). Protective Effect of Cyanidin-3-O-Glucoside against Ultraviolet B Radiation-Induced Cell Damage in Human HaCaT Keratinocytes. Front. Pharmacol..

[390] Zhang T., Jiang S., He C., Kimura Y., Yamashita Y., Ashida H. (2013). Black soybean seed coat polyphenols prevent B(a)P-induced DNA damage through modulating drug-metabolizing enzymes in HepG2 cells and ICR mice. Mutat. Res..

[391] Norris K.M., Okie W., Yakaitis C.L., Pazdro R. (2016). The anthocyanin cyanidin-3-O-β-glucoside modulates murine glutathione homeostasis in a manner dependent on genetic background. Redox Biol..

[392] Samadder A., Tarafdar D., Das R., Khuda-Bukhsh A.R., Abraham S.K. (2019). Efficacy of nanoencapsulated pelargonidin in ameliorating pesticide toxicity in fish and L6 cells: Modulation of oxidative stress and signalling cascade. Sci. Total Environ..

[393] Sharath Babu G.R., Anand T., Ilaiyaraja N., Khanum F., Gopalan N. (2017). Pelargonidin Modulates Keap1/Nrf2 Pathway Gene Expression and Ameliorates Citrinin-Induced Oxidative Stress in HepG2 Cells. Front. Pharmacol..

[394] Samadder A., Abraham S.K., Khuda-Bukhsh A.R. (2016). Nanopharmaceutical approach using pelargonidin towards enhancement of efficacy for prevention of alloxan-induced DNA damage in L6 cells via activation of PARP and p53. Environ. Toxicol. Pharmacol..

[395] Li S., Li W., Wang C., Wu R., Yin R., Kuo H.C., Wang L., Kong A.N. (2019). Pelargonidin reduces the TPA induced transformation of mouse epidermal cells -potential involvement of Nrf2 promoter demethylation. Chem. Interact..

[396] Singletary K.W., Jung K.-J., Giusti M. (2007). Anthocyanin-rich grape extract blocks breast cell DNA damage. J. Med. Food.

[397] Bankoglu E.E., Broscheit J., Arnaudov T., Roewer N., Stopper H. (2018). Protective effects of tricetinidin against oxidative stress inducers in rat kidney cells: A comparison with delphinidin and standard antioxidants. Food Chem. Toxicol. Int. J. Publ. Br. Ind. Biol. Res. Assoc..

[398] Kim H.M., Kim S.H., Kang B.S. (2018). Radioprotective effects of delphinidin on normal human lung cells against proton beam exposure. Nutr. Res. Pract..

[399] Aichinger G., Puntscher H., Beisl J., Kütt M.-L., Warth B., Marko D. (2018). Delphinidin protects colon carcinoma cells against the genotoxic effects of the mycotoxin altertoxin II. Toxicol. Lett..

[400] Prasad R., Singh T., Katiyar S.K. (2017). Honokiol inhibits ultraviolet radiation-induced immunosuppression through inhibition of ultraviolet-induced inflammation and DNA hypermethylation in mouse skin. Sci. Rep..

[401] Wang M., Li Y., Ni C., Song G. (2017). Honokiol Attenuates Oligomeric Amyloid β1-42-Induced Alzheimer’s Disease in Mice Through Attenuating Mitochondrial Apoptosis and Inhibiting the Nuclear Factor Kappa-B Signaling Pathway. Cell. Physiol. Biochem. Int. J. Exp. Cell. Physiol. Biochem. Pharmacol..

[402] Park C., Choi S.H., Jeong J.W., Han M.H., Lee H., Hong S.H., Kim G.Y., Moon S.K., Kim W.J., Choi Y.H. (2020). Honokiol ameliorates oxidative stress-induced DNA damage and apoptosis of c2c12 myoblasts by ROS generation and mitochondrial pathway. Anim. Cells Syst..

[403] Ruankham W., Suwanjang W., Wongchitrat P., Prachayasittikul V., Prachayasittikul S., Phopin K. (2019). Sesamin and sesamol attenuate H_2_O_2_-induced oxidative stress on human neuronal cells via the SIRT1-SIRT3-FOXO3a signaling pathway. Nutr. Neurosci..

[404] Le T.D., Nakahara Y., Ueda M., Okumura K., Hirai J., Sato Y., Takemoto D., Tomimori N., Ono Y., Nakai M. (2019). Sesamin suppresses aging phenotypes in adult muscular and nervous systems and intestines in a Drosophila senescence-accelerated model. Eur. Rev. Med. Pharmacol. Sci..

[405] Rousta A.M., Mirahmadi S.M.S., Shahmohammadi A., Nourabadi D., Khajevand-Khazaei M.R., Baluchnejadmojarad T., Roghani M. (2018). Protective effect of sesamin in lipopolysaccharide-induced mouse model of acute kidney injury via attenuation of oxidative stress, inflammation, and apoptosis. Immunopharmacol. Immunotoxicol..

[406] Xu Z., Liu Y., Yang D., Yuan F., Ding J., Chen H., Tian H. (2017). Sesamin protects SH-SY5Y cells against mechanical stretch injury and promoting cell survival. BMC Neurosci..

[407] Liu C.-M., Zheng G.-H., Ming Q.-L., Chao C., Sun J.-M. (2013). Sesamin protects mouse liver against nickel-induced oxidative DNA damage and apoptosis by the PI3K-Akt pathway. J. Agric. Food Chem..

[408] Bournival J., Francoeur M.-A., Renaud J., Martinoli M.-G. (2012). Quercetin and sesamin protect neuronal PC12 cells from high-glucose-induced oxidation, nitrosative stress, and apoptosis. Rejuvenation Res..

[409] Kanimozhi P., Prasad N.R. (2009). Antioxidant potential of sesamol and its role on radiation-induced DNA damage in whole-body irradiated Swiss albino mice. Environ. Toxicol. Pharmacol..

[410] Mishra K., Srivastava P.S., Chaudhury N.K. (2011). Sesamol as a potential radioprotective agent: In vitro studies. Radiat. Res..

[411] Ramachandran S., Rajendra Prasad N., Karthikeyan S. (2010). Sesamol inhibits UVB-induced ROS generation and subsequent oxidative damage in cultured human skin dermal fibroblasts. Arch. Dermatol. Res..

[412] Prasad N.R., Menon V.P., Vasudev V., Pugalendi K.V. (2005). Radioprotective effect of sesamol on gamma-radiation induced DNA damage, lipid peroxidation and antioxidants levels in cultured human lymphocytes. Toxicology.

[413] Huang Y.T., Chen Y.Y., Lai Y.H., Cheng C.C., Lin T.C., Su Y.S., Liu C.H., Lai P.C. (2016). Resveratrol alleviates the cytotoxicity induced by the radiocontrast agent, ioxitalamate, by reducing the production of reactive oxygen species in HK-2 human renal proximal tubule epithelial cells in vitro. Int. J. Mol. Med..

[414] Neyra Recky J.R., Gaspar Tosato M., Serrano M.P., Thomas A.H., Dántola M.L., Lorente C. (2019). Evidence of the effectiveness of Resveratrol in the prevention of guanine one-electron oxidation: Possible benefits in cancer prevention. Phys. Chem. Chem. Phys..

[415] Leonard S.S., Xia C., Jiang B.H., Stinefelt B., Klandorf H., Harris G.K., Shi X. (2003). Resveratrol scavenges reactive oxygen species and effects radical-induced cellular responses. Biochem. Biophys. Res. Commun..

[416] Sengottuvelan M., Deeptha K., Nalini N. (2009). Resveratrol ameliorates DNA damage, prooxidant and antioxidant imbalance in 1,2-dimethylhydrazine induced rat colon carcinogenesis. Chem. Interact..

[417] Kang H.J., Hong Y.B., Kim H.J., Wang A., Bae I. (2012). Bioactive food components prevent carcinogenic stress via Nrf2 activation in BRCA1 deficient breast epithelial cells. Toxicol. Lett..

[418] Katen A.L., Stanger S.J., Anderson A.L., Nixon B., Roman S.D. (2016). Chronic acrylamide exposure in male mice induces DNA damage to spermatozoa; Potential for amelioration by resveratrol. Reprod. Toxicol..

[419] Zargar S., Alonazi M., Rizwana H., Wani T.A. (2019). Resveratrol Reverses Thioacetamide-Induced Renal Assault with respect to Oxidative Stress, Renal Function, DNA Damage, and Cytokine Release in Wistar Rats. Oxid. Med. Cell. Longev..

[420] Jin J., Li Y., Zhang X., Chen T., Wang Y., Wang Z. (2016). Evaluation of Both Free Radical Scavenging Capacity and Antioxidative Damage Effect of Polydatin. Adv. Exp. Med. Biol..

[421] Ince S., Avdatek F., Demirel H.H., Arslan-Acaroz D., Goksel E., Kucukkurt I. (2016). Ameliorative effect of polydatin on oxidative stress-mediated testicular damage by chronic arsenic exposure in rats. Andrologia.

[422] Ince S., Arslan Acaroz D., Neuwirth O., Demirel H.H., Denk B., Kucukkurt I., Turkmen R. (2014). Protective effect of polydatin, a natural precursor of resveratrol, against cisplatin-induced toxicity in rats. Food Chem. Toxicol. Int. J. Publ. Br. Ind. Biol. Res. Assoc..

[423] Lai Y., Zhou C., Huang P., Dong Z., Mo C., Xie L., Lin H., Zhou Z., Deng G., Liu Y. (2018). Polydatin alleviated alcoholic liver injury in zebrafish larvae through ameliorating lipid metabolism and oxidative stress. J. Pharmacol. Sci..

[424] Arslan-Acaroz D., Zemheri F., Demirel H.H., Kucukkurt I., Ince S., Eryavuz A. (2018). In vivo assessment of polydatin, a natural polyphenol compound, on arsenic-induced free radical overproduction, gene expression, and genotoxicity. Environ. Sci. Pollut. Res. Int..

[425] Balupillai A., Nagarajan R.P., Ramasamy K., Govindasamy K., Muthusamy G. (2018). Caffeic acid prevents UVB radiation induced photocarcinogenesis through regulation of PTEN signaling in human dermal fibroblasts and mouse skin. Toxicol. Appl. Pharmacol..

[426] Adjimani J.P., Asare P. (2015). Antioxidant and free radical scavenging activity of iron chelators. Toxicol. Rep..

[427] Li Y., Chen L.J., Jiang F., Yang Y., Wang X.X., Zhang Z., Li Z., Li L. (2015). Caffeic acid improves cell viability and protects against DNA damage: Involvement of reactive oxygen species and extracellular signal-regulated kinase. Braz. J. Med. Biol. Res..

[428] Coelho V.R., Vieira C.G., de Souza L.P., Moysés F., Basso C., Papke D.K., Pires T.R., Siqueira I.R., Picada J.N., Pereira P. (2015). Antiepileptogenic, antioxidant and genotoxic evaluation of rosmarinic acid and its metabolite caffeic acid in mice. Life Sci..

[429] Wang T., Chen L., Wu W., Long Y., Wang R. (2008). Potential cytoprotection: Antioxidant defence by caffeic acid phenethyl ester against free radical-induced damage of lipids, DNA, and proteins. Can. J. Physiol. Pharmacol..

[430] Sestili P., Diamantini G., Bedini A., Cerioni L., Tommasini I., Tarzia G., Cantoni O. (2002). Plant-derived phenolic compounds prevent the DNA single-strand breakage and cytotoxicity induced by tert-butylhydroperoxide via an iron-chelating mechanism. Biochem. J..

[431] Kitsati N., Fokas D., Ouzouni M.-D., Mantzaris M.D., Barbouti A., Galaris D. (2012). Lipophilic caffeic acid derivatives protect cells against H2O2-Induced DNA damage by chelating intracellular labile iron. J. Agric. Food Chem..

[432] Rehman M.U., Sultana S. (2011). Attenuation of oxidative stress, inflammation and early markers of tumor promotion by caffeic acid in Fe-NTA exposed kidneys of Wistar rats. Mol. Cell. Biochem..

[433] Sevgi K., Tepe B., Sarikurkcu C. (2015). Antioxidant and DNA damage protection potentials of selected phenolic acids. Food Chem. Toxicol. Int. J. Publ. Br. Ind. Biol. Res. Assoc..

[434] Cho Y.H., Bahuguna A., Kim H.H., Kim D.I., Kim H.J., Yu J.M., Jung H.G., Jang J.Y., Kwak J.H., Park G.H. (2017). Potential effect of compounds isolated from Coffea arabica against UV-B induced skin damage by protecting fibroblast cells. J. Photochem. Photobiol. B Biol..

[435] Ramos A.A., Marques F., Fernandes-Ferreira M., Pereira-Wilson C. (2013). Water extracts of tree Hypericum sps. protect DNA from oxidative and alkylating damage and enhance DNA repair in colon cells. Food Chem. Toxicol. Int. J. Publ. Br. Ind. Biol. Res. Assoc..

[436] Chen L., Li Y., Yin W., Shan W., Dai J., Yang Y., Li L. (2016). Combination of chlorogenic acid and salvianolic acid B protects against polychlorinated biphenyls-induced oxidative stress through Nrf2. Environ. Toxicol. Pharmacol..

[437] Fernando P.M., Piao M.J., Kang K.A., Ryu Y.S., Hewage S.R., Chae S.W., Hyun J.W. (2016). Rosmarinic Acid Attenuates Cell Damage against UVB Radiation-Induced Oxidative Stress via Enhancing Antioxidant Effects in Human HaCaT Cells. Biomol. Ther..

[438] Ding Y., Zhang Z., Yue Z., Ding L., Zhou Y., Huang Z., Huang H. (2019). Rosmarinic Acid Ameliorates H_2_O_2_-Induced Oxidative Stress in L02 Cells Through MAPK and Nrf2 Pathways. Rejuvenation Res..

[439] Ghaffari H., Venkataramana M., Jalali Ghassam B., Chandra Nayaka S., Nataraju A., Geetha N.P., Prakash H.S. (2014). Rosmarinic acid mediated neuroprotective effects against H_2_O_2_-induced neuronal cell damage in N2A cells. Life Sci..

[440] Eskandari H., Ehsanpour A.A., Al-Mansour N., Bardania H., Sutherland D., Mohammad-Beigi H. (2020). Rosmarinic acid inhibits programmed cell death in *Solanum tuberosum* L. calli under high salinity. Plant Physiol. Biochem..

[441] Taner G., Özkan Vardar D., Aydin S., Aytaç Z., Başaran A., Başaran N. (2017). Use of in vitro assays to assess the potential cytotoxic, genotoxic and antigenotoxic effects of vanillic and cinnamic acid. Drug Chem. Toxicol..

[442] Anlar H.G., Bacanlı M., Çal T., Aydın S., Arı N., Ündeğer Bucurgat Ü., Başaran A.A., Başaran A.N. (2018). Effects of cinnamic acid on complications of diabetes. Turk. J. Med. Sci..

[443] Sunitha M.C., Dhanyakrishnan R., PrakashKumar B., Nevin K.G. (2018). *p*-Coumaric acid mediated protection of H9c2 cells from Doxorubicin-induced cardiotoxicity: Involvement of augmented Nrf2 and autophagy. Biomed. Pharmacother..

[444] Prasanna N., Krishnan D.N., Rasool M. (2013). Sodium arsenite-induced cardiotoxicity in rats: Protective role of *p*-coumaric acid, a common dietary polyphenol. Toxicol. Mech. Methods.

[445] Lodovici M., Raimondi L., Guglielmi F., Gemignani S., Dolara P. (2003). Protection against ultraviolet B-induced oxidative DNA damage in rabbit corneal-derived cells (SIRC) by 4-coumaric acid. Toxicology.

[446] Shanthakumar J., Karthikeyan A., Bandugula V.R., Rajendra Prasad N. (2012). Ferulic acid, a dietary phenolic acid, modulates radiation effects in Swiss albino mice. Eur. J. Pharmacol..

[447] Das U., Manna K., Khan A., Sinha M., Biswas S., Sengupta A., Chakraborty A., Dey S. (2017). Ferulic acid (FA) abrogates γ-radiation induced oxidative stress and DNA damage by up-regulating nuclear translocation of Nrf2 and activation of NHEJ pathway. Free Radic. Res..

[448] Das U., Biswas S., Sengupta A., Manna K., Chakraborty A., Dey S. (2016). Ferulic acid (FA) abrogates ionizing radiation-induced oxidative damage in murine spleen. Int. J. Radiat. Biol..

[449] Ghosh S., Chowdhury S., Sarkar P., Sil P.C. (2018). Ameliorative role of ferulic acid against diabetes associated oxidative stress induced spleen damage. Food Chem. Toxicol. Int. J. Publ. Br. Ind. Biol. Res. Assoc..

[450] Kelainy E.G., Ibrahim Laila I.M., Ibrahim S.R. (2019). The effect of ferulic acid against lead-induced oxidative stress and DNA damage in kidney and testes of rats. Environ. Sci. Pollut. Res. Int..

[451] Bao Y., Chen Q., Xie Y., Tao Z., Jin K., Chen S., Bai Y., Yang J., Shan S. (2019). Ferulic acid attenuates oxidative DNA damage and inflammatory responses in microglia induced by benzo(a)pyrene. Int. Immunopharmacol..

[452] Aslan A., Gok O., Beyaz S., Arslan E., Erman O., Agca C.A. (2020). The preventive effect of ellagic acid on brain damage in rats via regulating of Nrf-2, NF-kB and apoptotic pathway. J. Food Biochem..

[453] Mottola F., Scudiero N., Iovine C., Santonastaso M., Rocco L. (2020). Protective activity of ellagic acid in counteract oxidative stress damage in zebrafish embryonic development. Ecotoxicol. Environ. Saf..

[454] Hseu Y.C., Chou C.W., Senthil Kumar K.J., Fu K.T., Wang H.M., Hsu L.S., Kuo Y.H., Wu C.R., Chen S.C., Yang H.L. (2012). Ellagic acid protects human keratinocyte (HaCaT) cells against UVA-induced oxidative stress and apoptosis through the upregulation of the HO-1 and Nrf-2 antioxidant genes. Food Chem. Toxicol. Int. J. Publ. Br. Ind. Biol. Res. Assoc..

[455] Mishra S., Vinayak M. (2014). Ellagic acid inhibits PKC signaling by improving antioxidant defense system in murine T cell lymphoma. Mol. Biol. Rep..

[456] Aslan A., Gok O., Erman O., Kuloglu T. (2018). Ellagic acid impedes carbontetrachloride-induced liver damage in rats through suppression of NF-kB, Bcl-2 and regulating Nrf-2 and caspase pathway. Biomed. Pharmacother..

[457] Kavitha K., Thiyagarajan P., Rathna Nandhini J., Mishra R., Nagini S. (2013). Chemopreventive effects of diverse dietary phytochemicals against DMBA-induced hamster buccal pouch carcinogenesis via the induction of Nrf2-mediated cytoprotective antioxidant, detoxification, and DNA repair enzymes. Biochimie.

[458] Ferk F., Chakraborty A., Jäger W., Kundi M., Bichler J., Mišík M., Wagner K.H., Grasl-Kraupp B., Sagmeister S., Haidinger G. (2011). Potent protection of gallic acid against DNA oxidation: Results of human and animal experiments. Mutat. Res..

[459] Nair G.G., Nair C.K.K. (2013). Radioprotective effects of gallic acid in mice. BioMed Res. Int..

[460] Heo S.J., Ko S.C., Kang S.M., Cha S.H., Lee S.H., Kang D.H., Jung W.K., Affan A., Oh C., Jeon Y.J. (2010). Inhibitory effect of diphlorethohydroxycarmalol on melanogenesis and its protective effect against UV-B radiation-induced cell damage. Food Chem. Toxicol. Int. J. Publ. Br. Ind. Biol. Res. Assoc..

[461] Piao M.J., Kang K.A., Kim K.C., Chae S., Kim G.O., Shin T., Kim H.S., Hyun J.W. (2013). Diphlorethohydroxycarmalol attenuated cell damage against UVB radiation via enhancing antioxidant effects and absorbing UVB ray in human HaCaT keratinocytes. Environ. Toxicol. Pharmacol..

[462] Park C., Lee H., Hong S.H., Kim J.H., Park S.K., Jeong J.W., Kim G.Y., Hyun J.W., Yun S.J., Kim B.W. (2019). Protective effect of diphlorethohydroxycarmalol against oxidative stress-induced DNA damage and apoptosis in retinal pigment epithelial cells. Cutan. Ocul. Toxicol..

[463] Wu L.T., Chu C.C., Chung J.G., Chen C.H., Hsu L.S., Liu J.K., Chen S.C. (2004). Effects of tannic acid and its related compounds on food mutagens or hydrogen peroxide-induced DNA strands breaks in human lymphocytes. Mutat. Res..

[464] Silva R.M., Pereira L.D., Véras J.H., do Vale C.R., Chen-Chen L., da Costa Santos S. (2016). Protective effect and induction of DNA repair by Myrciaria cauliflora seed extract and pedunculagin on cyclophosphamide-induced genotoxicity. Mutat. Res..

[465] Labieniec M., Gabryelak T. (2005). Measurement of DNA damage and protein oxidation after the incubation of B14 Chinese hamster cells with chosen polyphenols. Toxicol. Lett..

[466] Yang B., Liu P. (2014). Composition and biological activities of hydrolyzable tannins of fruits of *Phyllanthus emblica*. J. Agric. Food Chem..

[467] Carvalho D.O., Oliveira R., Johansson B., Guido L.F. (2016). Dose-Dependent Protective and Inductive Effects of Xanthohumol on Oxidative DNA Damage in *Saccharomyces cerevisiae*. Food Technol. Biotechnol..

[468] Ferk F., Mišík M., Nersesyan A., Pichler C., Jäger W., Szekeres T., Marculescu R., Poulsen H.E., Henriksen T., Bono R. (2016). Impact of xanthohumol (a prenylated flavonoid from hops) on DNA stability and other health-related biochemical parameters: Results of human intervention trials. Mol. Nutr. Food Res..

[469] Dietz B.M., Kang Y.H., Liu G., Eggler A.L., Yao P., Chadwick L.R., Pauli G.F., Farnsworth N.R., Mesecar A.D., van Breemen R.B. (2005). Xanthohumol isolated from Humulus lupulus Inhibits menadione-induced DNA damage through induction of quinone reductase. Chem. Res. Toxicol..

[470] Pichler C., Ferk F., Al-Serori H., Huber W., Jäger W., Waldherr M., Mišík M., Kundi M., Nersesyan A., Herbacek I. (2017). Xanthohumol Prevents DNA Damage by Dietary Carcinogens: Results of a Human Intervention Trial. Cancer Prev. Res..

[471] Jamnongkan W., Thanee M., Yongvanit P., Loilome W., Thanan R., Kimawaha P., Boonmars T., Silakit R., Namwat N., Techasen A. (2018). Antifibrotic effect of xanthohumol in combination with praziquantel is associated with altered redox status and reduced iron accumulation during liver fluke-associated cholangiocarcinogenesis. PeerJ.

[472] Li Y., Li Z., Hou H., Zhuang Y., Sun L. (2018). Metal Chelating, Inhibitory DNA Damage, and Anti-Inflammatory Activities of Phenolics from Rambutan (*Nephelium lappaceum*) Peel and the Quantifications of Geraniin and Corilagin. Molecules.

[473] Koul A., Abraham S.K. (2018). Efficacy of crocin and safranal as protective agents against genotoxic stress induced by gamma radiation, urethane and procarbazine in mice. Hum. Exp. Toxicol..

[474] Masutani H., Otsuki R., Yamaguchi Y., Takenaka M., Kanoh N., Takatera K., Kunimoto Y., Yodoi J. (2009). Fragrant unsaturated aldehydes elicit activation of the Keap1/Nrf2 system leading to the upregulation of thioredoxin expression and protection against oxidative stress. Antioxid. Redox Signal..

[475] Sadeghnia H.R., Kamkar M., Assadpour E., Boroushaki M.T., Ghorbani A. (2013). Protective Effect of Safranal, a Constituent of Crocus sativus, on Quinolinic Acid-induced Oxidative Damage in Rat Hippocampus. Iran. J. Basic Med. Sci..

[476] Baluchnejadmojarad T., Mohamadi-Zarch S.M., Roghani M. (2019). Safranal, an active ingredient of saffron, attenuates cognitive deficits in amyloid beta-induced rat model of Alzheimer’s disease: Underlying mechanisms. Metab. Brain Dis..

[477] Bacanlı M., Başaran A.A., Başaran N. (2015). The antioxidant and antigenotoxic properties of citrus phenolics limonene and naringin. Food Chem. Toxicol. Int. J. Publ. Br. Ind. Biol. Res. Assoc..

[478] Bacanlı M., Anlar H.G., Aydın S., Çal T., Arı N., Ündeğer Bucurgat Ü., Başaran A.A., Başaran N. (2017). d-limonene ameliorates diabetes and its complications in streptozotocin-induced diabetic rats. Food Chem. Toxicol. Int. J. Publ. Br. Ind. Biol. Res. Assoc..

[479] Verma N., Yadav A., Bal S., Gupta R., Aggarwal N. (2019). In Vitro Studies on Ameliorative Effects of Limonene on Cadmium-Induced Genotoxicity in Cultured Human Peripheral Blood Lymphocytes. Appl. Biochem. Biotechnol..

[480] Thapa D., Richardson A.J., Zweifel B., Wallace R.J., Gratz S.W. (2019). Genoprotective Effects of Essential Oil Compounds against Oxidative and Methylated DNA Damage in Human Colon Cancer Cells. J. Food Sci..

[481] Zerrouki M., Benkaci-Ali F. (2018). DFT study of the mechanisms of nonenzymatic DNA repair by phytophenolic antioxidants. J. Mol. Model..

[482] Calò R., Visone C.M., Marabini L. (2015). Thymol and *Thymus vulgaris* L. activity against UVA- and UVB-induced damage in NCTC 2544 cell line. Mutat. Res. Genet. Toxicol. Environ. Mutagen..

[483] Horvathova E., Navarova J., Galova E., Sevcovicova A., Chodakova L., Snahnicanova Z., Melusova M., Kozics K., Slamenova D. (2014). Assessment of antioxidative, chelating, and DNA-protective effects of selected essential oil components (eugenol, carvacrol, thymol, borneol, eucalyptol) of plants and intact Rosmarinus officinalis oil. J. Agric. Food Chem..

[484] Archana P.R., Nageshwar Rao B., Satish Rao B.S. (2011). Modulation of gamma ray-induced genotoxic effect by thymol, a monoterpene phenol derivative of cymene. Integr. Cancer Ther..

[485] Archana P.R., Nageshwar Rao B., Ballal M., Satish Rao B.S. (2009). Thymol, a naturally occurring monocyclic dietary phenolic compound protects Chinese hamster lung fibroblasts from radiation-induced cytotoxicity. Mutat. Res..

[486] Aristatile B., Al-Numair K.S., Al-Assaf A.H., Veeramani C., Pugalendi K.V. (2015). Protective Effect of Carvacrol on Oxidative Stress and Cellular DNA Damage Induced by UVB Irradiation in Human Peripheral Lymphocytes. J. Biochem. Mol. Toxicol..

[487] Elhady M.A., Khalaf A.A.A., Kamel M.M., Noshy P.A. (2019). Carvacrol ameliorates behavioral disturbances and DNA damage in the brain of rats exposed to propiconazole. Neurotoxicology.

[488] Kılıç Y., Geyikoglu F., Çolak S., Turkez H., Bakır M., Hsseinigouzdagani M. (2016). Carvacrol modulates oxidative stress and decreases cell injury in pancreas of rats with acute pancreatitis. Cytotechnology.

[489] Banik S., Akter M., Corpus Bondad S.E., Saito T., Hosokawa T., Kurasaki M. (2019). Carvacrol inhibits cadmium toxicity through combating against caspase dependent/independent apoptosis in PC12cells. Food Chem. Toxicol..

[490] Hasan S.K., Sultana S. (2015). Geraniol attenuates 2-acetylaminofluorene induced oxidative stress, inflammation and apoptosis in the liver of wistar rats. Toxicol. Mech. Methods.

[491] Jahangir T., Sultana S. (2008). Benzo(a)pyrene-induced genotoxicity: Attenuation by farnesol in a mouse model. J. Enzym. Inhib. Med. Chem..

[492] Horváth B., Mukhopadhyay P., Kechrid M., Patel V., Tanchian G., Wink D.A., Gertsch J., Pacher P. (2012). β-Caryophyllene ameliorates cisplatin-induced nephrotoxicity in a cannabinoid 2 receptor-dependent manner. Free Radic. Biol. Med..

[493] Di Giacomo S., Abete L., Cocchiola R., Mazzanti G., Eufemi M., Di Sotto A. (2018). Caryophyllane sesquiterpenes inhibit DNA-damage by tobacco smoke in bacterial and mammalian cells. Food Chem. Toxicol. Int. J. Publ. Br. Ind. Biol. Res. Assoc..

[494] Al-Taee H., Azimullah S., Meeran M., Alaraj Almheiri M.K., Al Jasmi R.A., Tariq S., Ab Khan M., Adeghate E., Ojha S. (2019). β-caryophyllene, a dietary phytocannabinoid attenuates oxidative stress, inflammation, apoptosis and prevents structural alterations of the myocardium against doxorubicin-induced acute cardiotoxicity in rats: An in vitro and in vivo study. Eur. J. Pharmacol..

[495] Chavez-Hurtado P., Gonzalez-Castaneda R.E., Beas-Zarate C., Flores-Soto M.E., Viveros-Paredes J.M. (2020). β-Caryophyllene Reduces DNA Oxidation and the Overexpression of Glial Fibrillary Acidic Protein in the Prefrontal Cortex and Hippocampus of d-Galactose-Induced Aged BALB/c Mice. J. Med. Food.

[496] Horváthová E., Kozics K., Srančíková A., Hunáková L., Gálová E., Ševčovičová A., Slameňová D. (2012). Borneol administration protects primary rat hepatocytes against exogenous oxidative DNA damage. Mutagenesis.

[497] Wang H., Sim M.K., Loke W.K., Chinnathambi A., Alharbi S.A., Tang F.R., Sethi G. (2017). Potential Protective Effects of Ursolic Acid against Gamma Irradiation-Induced Damage Are Mediated through the Modulation of Diverse Inflammatory Mediators. Front. Pharmacol..

[498] Ramachandran S., Prasad N.R. (2008). Effect of ursolic acid, a triterpenoid antioxidant, on ultraviolet-B radiation-induced cytotoxicity, lipid peroxidation and DNA damage in human lymphocytes. Chem. Interact..

[499] Radhiga T., Rajamanickam C., Sundaresan A., Ezhumalai M., Pugalendi K.V. (2012). Effect of ursolic acid treatment on apoptosis and DNA damage in isoproterenol-induced myocardial infarction. Biochimie.

[500] Ma J.-Q., Ding J., Xiao Z.-H., Liu C.-M. (2014). Ursolic acid ameliorates carbon tetrachloride-induced oxidative DNA damage and inflammation in mouse kidney by inhibiting the STAT3 and NF-κB activities. Int. Immunopharmacol..

[501] Yang Y., Yin R., Wu R., Ramirez C.N., Sargsyan D., Li S., Wang L., Cheng D., Wang C., Hudlikar R. (2019). DNA methylome and transcriptome alterations and cancer prevention by triterpenoid ursolic acid in UVB-induced skin tumor in mice. Mol. Carcinog..

[502] Sarkar C., Pal S., Das N., Dinda B. (2014). Ameliorative effects of oleanolic acid on fluoride induced metabolic and oxidative dysfunctions in rat brain: Experimental and biochemical studies. Food Chem. Toxicol. Int. J. Publ. Br. Ind. Biol. Res. Assoc..

[503] Allouche Y., Warleta F., Campos M., Sánchez-Quesada C., Uceda M., Beltrán G., Gaforio J.J. (2011). Antioxidant, antiproliferative, and pro-apoptotic capacities of pentacyclic triterpenes found in the skin of olives on MCF-7 human breast cancer cells and their effects on DNA damage. J. Agric. Food Chem..

[504] Srivastava A.K., Mishra S., Ali W., Shukla Y. (2016). Protective effects of lupeol against mancozeb-induced genotoxicity in cultured human lymphocytes. Phytomed. Int. J. Phytother. Phytopharm..

[505] Nigam N., Prasad S., Shukla Y. (2007). Preventive effects of lupeol on DMBA induced DNA alkylation damage in mouse skin. Food Chem. Toxicol. Int. J. Publ. Br. Ind. Biol. Res. Assoc..

[506] Kumari A., Kakkar P. (2012). Lupeol protects against acetaminophen-induced oxidative stress and cell death in rat primary hepatocytes. Food Chem. Toxicol. Int. J. Publ. Br. Ind. Biol. Res. Assoc..

[507] Kumari A., Kakkar P. (2012). Lupeol prevents acetaminophen-induced in vivo hepatotoxicity by altering the Bax/Bcl-2 and oxidative stress-mediated mitochondrial signaling cascade. Life Sci..

[508] Lü J.-M., Weakley S.M., Yang Z., Hu M., Yao Q., Chen C. (2012). Ginsenoside Rb1 directly scavenges hydroxyl radical and hypochlorous acid. Curr. Pharm. Des..

[509] Shuangyan W., Ruowu S., Hongli N., Bei Z., Yong S. (2012). Protective effects of Rg_2_ on hypoxia-induced neuronal damage in hippocampal neurons. Artif. Cells Blood Substit. Biotechnol..

[510] Seo B.-Y., Choi M.-J., Kim J.-S., Park E. (2019). Comparative Analysis of Ginsenoside Profiles: Antioxidant, Antiproliferative, and Antigenotoxic Activities of Ginseng Extracts of Fine and Main Roots. Prev. Nutr. Food Sci..

[511] Li J., Cai D., Yao X., Zhang Y., Chen L., Jing P., Wang L., Wang Y. (2016). Protective Effect of Ginsenoside Rg1 on Hematopoietic Stem/Progenitor Cells through Attenuating Oxidative Stress and the Wnt/β-Catenin Signaling Pathway in a Mouse Model of d-Galactose-induced Aging. Int. J. Mol. Sci..

[512] Jiang G.-Z., Li J.-C. (2014). Protective effects of ginsenoside Rg1 against colistin sulfate-induced neurotoxicity in PC12 cells. Cell. Mol. Neurobiol..

[513] Poon P.Y., Kwok H.H., Yue P.Y., Yang M.S., Mak N.K., Wong C.K., Wong R.N. (2012). Cytoprotective effect of 20S-Rg3 on benzo[a]pyrene-induced DNA damage. Drug Metab. Dispos. Biol. Fate Chem..

[514] Quan Y., Yang Y., Wang H., Shu B., Gong Q.-H., Qian M. (2015). Gypenosides attenuate cholesterol-induced DNA damage by inhibiting the production of reactive oxygen species in human umbilical vein endothelial cells. Mol. Med. Rep..

[515] Zhang G.L., Deng J.P., Wang B.H., Zhao Z.W., Li J., Gao L., Liu B.L., Xong J.R., Guo X.D., Yan Z.Q. (2011). Gypenosides improve cognitive impairment induced by chronic cerebral hypoperfusion in rats by suppressing oxidative stress and astrocytic activation. Behav. Pharmacol..

[516] Zhang G., Zhao Z., Gao L., Deng J., Wang B., Xu D., Liu B., Qu Y., Yu J., Li J. (2011). Gypenoside attenuates white matter lesions induced by chronic cerebral hypoperfusion in rats. Pharmacol. Biochem. Behav..

[517] Kwok H.H., Ng W.Y., Yang M.S.M., Mak N.K., Wong R.N.S., Yue P.Y.K. (2010). The ginsenoside protopanaxatriol protects endothelial cells from hydrogen peroxide-induced cell injury and cell death by modulating intracellular redox status. Free Radic. Biol. Med..

[518] Veratti E., Rossi T., Giudice S., Benassi L., Bertazzoni G., Morini D., Azzoni P., Bruni E., Giannetti A., Magnoni C. (2011). 18β-glycyrrhetinic acid and glabridin prevent oxidative DNA fragmentation in UVB-irradiated human keratinocyte cultures. Anticancer Res..

[519] Lefaki M., Papaevgeniou N., Tur J.A., Vorgias C.E., Sykiotis G.P., Chondrogianni N. (2020). The dietary triterpenoid 18α-Glycyrrhetinic acid protects from MMC-induced genotoxicity through the ERK/Nrf2 pathway. Redox Biol..

[520] Gandhi N.M., Maurya D.K., Salvi V., Kapoor S., Mukherjee T., Nair C.K.K. (2004). Radioprotection of DNA by glycyrrhizic acid through scavenging free radicals. J. Radiat. Res..

[521] Umar S.A., Tanveer M.A., Nazir L.A., Divya G., Vishwakarma R.A., Tasduq S.A. (2019). Glycyrrhizic Acid Prevents Oxidative Stress Mediated DNA Damage Response through Modulation of Autophagy in Ultraviolet-B-Irradiated Human Primary Dermal Fibroblasts. Cell. Physiol. Biochem. Int. J. Exp. Cell. Physiol. Biochem. Pharmacol..

[522] Arjumand W., Sultana S. (2011). Glycyrrhizic acid: A phytochemical with a protective role against cisplatin-induced genotoxicity and nephrotoxicity. Life Sci..

[523] Santocono M., Zurria M., Berrettini M., Fedeli D., Falcioni G. (2006). Influence of astaxanthin, zeaxanthin and lutein on DNA damage and repair in UVA-irradiated cells. J. Photochem. Photobiol. B Biol..

[524] Dong L.-Y., Jin J., Lu G., Kang X.-L. (2013). Astaxanthin attenuates the apoptosis of retinal ganglion cells in db/db mice by inhibition of oxidative stress. Drugs.

[525] Lyons N.M., O’Brien N.M. (2002). Modulatory effects of an algal extract containing astaxanthin on UVA-irradiated cells in culture. J. Dermatol. Sci..

[526] Park J.S., Mathison B.D., Hayek M.G., Zhang J., Reinhart G.A., Chew B.P. (2013). Astaxanthin modulates age-associated mitochondrial dysfunction in healthy dogs. J. Anim. Sci..

[527] Nakajima Y., Inokuchi Y., Shimazawa M., Otsubo K., Ishibashi T., Hara H. (2008). Astaxanthin, a dietary carotenoid, protects retinal cells against oxidative stress in-vitro and in mice in-vivo. J. Pharm. Pharmacol..

[528] Turkez H., Geyikoglu F., Yousef M.I., Togar B., Gürbüz H., Celik K., Akbaba G.B., Polat Z. (2014). Hepatoprotective potential of astaxanthin against 2,3,7,8-tetrachlorodibenzo-p-dioxin in cultured rat hepatocytes. Toxicol. Ind. Health.

[529] Zheng J., Piao M.J., Keum Y.S., Kim H.S., Hyun J.W. (2013). Fucoxanthin Protects Cultured Human Keratinocytes against Oxidative Stress by Blocking Free Radicals and Inhibiting Apoptosis. Biomol. Ther..

[530] Heo S.-J., Jeon Y.-J. (2009). Protective effect of fucoxanthin isolated from Sargassum siliquastrum on UV-B induced cell damage. J. Photochem. Photobiol. B Biol..

[531] Liu C.L., Liang A.L., Hu M.L. (2011). Protective effects of fucoxanthin against ferric nitrilotriacetate-induced oxidative stress in murine hepatic BNL CL.2 cells. Toxicol. Vitr. Int. J. Publ. Assoc. BIBRA.

[532] Pangestuti R., Vo T.-S., Ngo D.-H., Kim S.-K. (2013). Fucoxanthin ameliorates inflammation and oxidative reponses in microglia. J. Agric. Food Chem..

[533] Firdous A.P., Sindhu E.R., Ramnath V., Kuttan R. (2013). Amelioration of radiation-induced damages in mice by carotenoid meso-zeaxanthin. Int. J. Radiat. Biol..

[534] Kowluru R.A., Menon B., Gierhart D.L. (2008). Beneficial effect of zeaxanthin on retinal metabolic abnormalities in diabetic rats. Investig. Ophthalmol. Vis. Sci..

[535] Santocono M., Zurria M., Berrettini M., Fedeli D., Falcioni G. (2007). Lutein, zeaxanthin and astaxanthin protect against DNA damage in SK-N-SH human neuroblastoma cells induced by reactive nitrogen species. J. Photochem. Photobiol. B Biol..

[536] Serpeloni J.M., Cólus I.M., de Oliveira F.S., Aissa A.F., Mercadante A.Z., Bianchi M.L., Antunes L.M. (2014). Diet carotenoid lutein modulates the expression of genes related to oxygen transporters and decreases DNA damage and oxidative stress in mice. Food Chem. Toxicol. Int. J. Publ. Br. Ind. Biol. Res. Assoc..

[537] Serpeloni J.M., Barcelos G.R.M., Friedmann Angeli J.P., Mercadante A.Z., Lourdes Pires Bianchi M., Antunes L.M.G. (2012). Dietary carotenoid lutein protects against DNA damage and alterations of the redox status induced by cisplatin in human derived HepG2 cells. Toxicol. Vitr. Int. J. Publ. Assoc. BIBRA.

[538] Serpeloni J.M., Grotto D., Mercadante A.Z., de Lourdes Pires Bianchi M., Antunes L.M.G. (2010). Lutein improves antioxidant defense in vivo and protects against DNA damage and chromosome instability induced by cisplatin. Arch. Toxicol..

[539] Lim S., Hwang S., Yu J.H., Lim J.W., Kim H. (2017). Lycopene inhibits regulator of calcineurin 1-mediated apoptosis by reducing oxidative stress and down-regulating Nucling in neuronal cells. Mol. Nutr. Food Res..

[540] Tokaç M., Aydin S., Taner G., Özkardeş A.B., Yavuz Taşlipinar M., Doğan M., Dündar H.Z., Kiliç M., Başaran A.A., Başaran A.N. (2015). Hepatoprotective and antioxidant effects of lycopene in acute cholestasis. Turk. J. Med. Sci..

[541] Banji D., Banji O.J.F., Reddy M., Annamalai A.R. (2013). Impact of zinc, selenium and lycopene on capsaicin induced mutagenicity and oxidative damage in mice. J. Trace Elem. Med. Biol. Organ Soc. Miner. Trace Elem..

[542] Kim J.Y., Paik J.K., Kim O.Y., Park H.W., Lee J.H., Jang Y., Lee J.H. (2011). Effects of lycopene supplementation on oxidative stress and markers of endothelial function in healthy men. Atherosclerosis.

[543] Campos K., de Oliveira Ramos C., Martins T.L., Costa G.P., Talvani A., Garcia C., Oliveira L., Cangussú S.D., Costa D.C., Bezerra F.S. (2019). Lycopene mitigates pulmonary emphysema induced by cigarette smoke in a murine model. J. Nutr. Biochem..

[544] Srinivasan M., Sudheer A.R., Pillai K.R., Kumar P.R., Sudhakaran P.R., Menon V.P. (2007). Lycopene as a natural protector against gamma-radiation induced DNA damage, lipid peroxidation and antioxidant status in primary culture of isolated rat hepatocytes in vitro. Biochim. Biophys. Acta.

[545] Abdel-Rahman H.G., Abdelrazek H.M.A., Zeidan D.W., Mohamed R.M., Abdelazim A.M. (2018). Lycopene: Hepatoprotective and Antioxidant Effects toward Bisphenol A-Induced Toxicity in Female Wistar Rats. Oxid. Med. Cell. Longev..

[546] Abass M.A., Elkhateeb S.A., Abd El-Baset S.A., Kattaia A.A., Mohamed E.M., Atteia H.H. (2016). Lycopene ameliorates atrazine-induced oxidative damage in adrenal cortex of male rats by activation of the Nrf2/HO-1 pathway. Environ. Sci. Pollut. Res. Int..

[547] Jang S.H., Lim J.W., Morio T., Kim H. (2012). Lycopene inhibits Helicobacter pylori-induced ATM/ATR-dependent DNA damage response in gastric epithelial AGS cells. Free Radic. Biol. Med..

[548] Rojo de la Vega M., Zhang D.D., Wondrak G.T. (2018). Topical Bixin Confers NRF2-Dependent Protection against Photodamage and Hair Graying in Mouse Skin. Front. Pharmacol..

[549] Tao S., Rojo de la Vega M., Quijada H., Wondrak G.T., Wang T., Garcia J.G., Zhang D.D. (2016). Bixin protects mice against ventilation-induced lung injury in an NRF2-dependent manner. Sci. Rep..

[550] Barcelos G.R., Grotto D., Serpeloni J.M., Aissa A.F., Antunes L.M., Knasmüller S., Barbosa F. (2012). Bixin and norbixin protect against DNA-damage and alterations of redox status induced by methylmercury exposure in vivo. Environ. Mol. Mutagen..

[551] Ben Salem I., Boussabbeh M., Kantaoui H., Bacha H., Abid-Essefi S. (2016). Crocin, the main active saffron constituent, mitigates dichlorvos-induced oxidative stress and apoptosis in HCT-116 cells. Biomed. Pharmacother..

[552] Ghiasian M., Khamisabadi F., Kheiripour N., Karami M., Haddadi R., Ghaleiha A., Taghvaei B., Oliaie S.S., Salehi M., Samadi P. (2019). Effects of crocin in reducing DNA damage, inflammation, and oxidative stress in multiple sclerosis patients: A double-blind, randomized, and placebo-controlled trial. J. Biochem. Mol. Toxicol..

[553] Xiong S., Patrushev N., Forouzandeh F., Hilenski L., Alexander R.W. (2015). PGC-1α Modulates Telomere Function and DNA Damage in Protecting against Aging-Related Chronic Diseases. Cell Rep..

[554] Suh J.H., Shigeno E.T., Morrow J.D., Cox B., Rocha A.E., Frei B., Hagen T.M. (2001). Oxidative stress in the aging rat heart is reversed by dietary supplementation with (R)-(alpha)-lipoic acid. FASEB J. Off. Publ. Fed. Am. Soc. Exp. Biol..

[555] Rageh M.M., El-Gebaly R.H. (2019). Antioxidant activities of α-lipoic acid free and nano-capsule inhibit the growth of Ehrlich carcinoma. Mol. Biol. Rep..

[556] Shukla S., Sharma Y., Shrivastava S. (2016). Reversal of Lead-Induced Acute Toxicity by Lipoic Acid with Nutritional Supplements in Male Wistar Rats. J. Environ. Pathol. Toxicol. Oncol. Off. Organ Int. Soc. Environ. Toxicol. Cancer.

[557] Zanichelli F., Capasso S., Di Bernardo G., Cipollaro M., Pagnotta E., Cartenì M., Casale F., Iori R., Giordano A., Galderisi U. (2012). Low concentrations of isothiocyanates protect mesenchymal stem cells from oxidative injuries, while high concentrations exacerbate DNA damage. Apoptosis Int. J. Program. Cell Death.

[558] Khaleel S.A., Raslan N.A., Alzokaky A.A., Ewees M.G., Ashour A.A., Abdel-Hamied H.E., Abd-Allah A.R. (2019). Contrast media (meglumine diatrizoate) aggravates renal inflammation, oxidative DNA damage and apoptosis in diabetic rats which is restored by sulforaphane through Nrf2/HO-1 reactivation. Chem. Interact..

[559] Thangapandiyan S., Ramesh M., Hema T., Miltonprabu S., Uddin M.S., Nandhini V., Bavithra Jothi G. (2019). Sulforaphane Potentially Ameliorates Arsenic Induced Hepatotoxicity in Albino Wistar Rats: Implication of PI3K/Akt/Nrf2 Signaling Pathway. Cell. Physiol. Biochem. Int. J. Exp. Cell. Physiol. Biochem. Pharmacol..

[560] Liu P., Wang W., Tang J., Bowater R.P., Bao Y. (2019). Antioxidant effects of sulforaphane in human HepG2 cells and immortalised hepatocytes. Food Chem. Toxicol. Int. J. Publ. Br. Ind. Biol. Res. Assoc..

[561] Thangapandiyan S., Ramesh M., Miltonprabu S., Hema T., Jothi G.B., Nandhini V. (2019). Sulforaphane potentially attenuates arsenic-induced nephrotoxicity via the PI3K/Akt/Nrf2 pathway in albino Wistar rats. Environ. Sci. Pollut. Res. Int..

[562] Hariton F., Xue M., Rabbani N., Fowler M., Thornalley P.J. (2018). Sulforaphane Delays Fibroblast Senescence by Curbing Cellular Glucose Uptake, Increased Glycolysis, and Oxidative Damage. Oxid. Med. Cell. Longev..

[563] Piberger A.L., Keil C., Platz S., Rohn S., Hartwig A. (2015). Sulforaphane inhibits damage-induced poly (ADP-ribosyl)ation via direct interaction of its cellular metabolites with PARP-1. Mol. Nutr. Food Res..

[564] Shang G., Tang X., Gao P., Guo F., Liu H., Zhao Z., Chen Q., Jiang T., Zhang N., Li H. (2015). Sulforaphane attenuation of experimental diabetic nephropathy involves GSK-3 beta/Fyn/Nrf2 signaling pathway. J. Nutr. Biochem..

[565] Talalay P., Fahey J.W., Healy Z.R., Wehage S.L., Benedict A.L., Min C., Dinkova-Kostova A.T. (2007). Sulforaphane mobilizes cellular defenses that protect skin against damage by UV radiation. Proc. Natl. Acad. Sci. USA.

[566] Anwar-Mohamed A., El-Kadi A.O.S. (2009). Down-regulation of the detoxifying enzyme NAD(P)H:quinone oxidoreductase 1 by vanadium in Hepa 1c1c7 cells. Toxicol. Appl. Pharmacol..

[567] Salah-Abbès J.B., Abbès S., Ouanes Z., Abdel-Wahhab M.A., Bacha H., Oueslati R. (2009). Isothiocyanate from the Tunisian radish (*Raphanus sativus*) prevents genotoxicity of Zearalenone in vivo and in vitro. Mutat. Res..

[568] Ha H.C., Sirisoma N.S., Kuppusamy P., Zweier J.L., Woster P.M., Casero R.A. (1998). The natural polyamine spermine functions directly as a free radical scavenger. Proc. Natl. Acad. Sci. USA.

[569] Ha H.C., Yager J.D., Woster P.A., Casero R.A. (1998). Structural specificity of polyamines and polyamine analogues in the protection of DNA from strand breaks induced by reactive oxygen species. Biochem. Biophys. Res. Commun..

[570] Yokozawa T., Ishida A., Kashiwada Y., Cho E.J., Kim H.Y., Ikeshiro Y. (2004). Coptidis Rhizoma: Protective effects against peroxynitrite-induced oxidative damage and elucidation of its active components. J. Pharm. Pharmacol..

[571] Li Z., Geng Y.-N., Jiang J.-D., Kong W.-J. (2014). Antioxidant and anti-inflammatory activities of berberine in the treatment of diabetes mellitus. Evid. Based Complement. Altern. Med. eCAM.

[572] Sadraie S., Kiasalari Z., Razavian M., Azimi S., Sedighnejad L., Afshin-Majd S., Baluchnejadmojarad T., Roghani M. (2019). Berberine ameliorates lipopolysaccharide-induced learning and memory deficit in the rat: Insights into underlying molecular mechanisms. Metab. Brain Dis..

[573] Hassani-Bafrani H., Najaran H., Razi M., Rashtbari H. (2019). Berberine ameliorates experimental varicocele-induced damages at testis and sperm levels; evidences for oxidative stress and inflammation. Andrologia.

[574] Zhao Z., Wei Q., Hua W., Liu Y., Liu X., Zhu Y. (2018). Hepatoprotective effects of berberine on acetaminophen-induced hepatotoxicity in mice. Biomed. Pharmacother..

[575] Sadeghnia H.R., Kolangikhah M., Asadpour E., Forouzanfar F., Hosseinzadeh H. (2017). Berberine protects against glutamate-induced oxidative stress and apoptosis in PC12 and N2a cells. Iran. J. Basic Med. Sci..

[576] Choi Y.H. (2016). Berberine Hydrochloride Protects C2C12 Myoblast Cells against Oxidative Stress-Induced Damage via Induction of Nrf-2-Mediated HO-1 Expression. Drug Dev. Res..

[577] Lu L., Jiang M., Zhu C., He J., Fan S. (2019). Amelioration of whole abdominal irradiation-induced intestinal injury in mice with 3,3′-Diindolylmethane (DIM). Free Radic. Biol. Med..

[578] Moiseeva E.P., Almeida G.M., Jones G.D.D., Manson M.M. (2007). Extended treatment with physiologic concentrations of dietary phytochemicals results in altered gene expression, reduced growth, and apoptosis of cancer cells. Mol. Cancer Ther..

[579] Hajra S., Basu A., Roy S.S., Patra A.R., Bhattacharya S. (2017). Attenuation of doxorubicin-induced cardiotoxicity and genotoxicity by an indole-based natural compound 3,3′-diindolylmethane (DIM) through activation of Nrf2/ARE signaling pathways and inhibiting apoptosis. Free Radic. Res..

[580] Lu L., Dong J., Li D., Zhang J., Fan S. (2016). 3,3′-diindolylmethane mitigates total body irradiation-induced hematopoietic injury in mice. Free Radic. Biol. Med..

[581] Scipioni M., Kay G., Megson I., Lin P.K.T. (2018). Novel vanillin derivatives: Synthesis, anti-oxidant, DNA and cellular protection properties. Eur. J. Med. Chem..

[582] Sefi M., Elwej A., Chaâbane M., Bejaoui S., Marrekchi R., Jamoussi K., Gouiaa N., Boudawara-Sellemi T., El Cafsi M., Zeghal N. (2019). Beneficial role of vanillin, a polyphenolic flavoring agent, on maneb-induced oxidative stress, DNA damage, and liver histological changes in Swiss albino mice. Hum. Exp. Toxicol..

[583] Ben Saad H., Driss D., Ben Amara I., Boudawara O., Boudawara T., Ellouz Chaabouni S., Mounir Zeghal K., Hakim A. (2016). Altered hepatic mRNA expression of immune response-associated DNA damage in mice liver induced by potassium bromate: Protective role of vanillin. Environ. Toxicol..

[584] Ben Saad H., Ben Amara I., Krayem N., Boudawara T., Kallel C., Zeghal K.M., Hakim A. (2015). Ameliorative effects of vanillin on potassium bromate induces bone and blood disorders in vivo. Cell. Mol. Biol..

[585] Makni M., Chtourou Y., Garoui E.M., Boudawara T., Fetoui H. (2012). Carbon tetrachloride-induced nephrotoxicity and DNA damage in rats: Protective role of vanillin. Hum. Exp. Toxicol..

[586] Fernando I., Dias M., Madusanka D., Han E.J., Kim M.J., Jeon Y.J., Lee K., Cheong S.H., Han Y.S., Park S.R. (2020). Human Keratinocyte UVB-Protective Effects of a Low Molecular Weight Fucoidan from Sargassum horneri Purified by Step Gradient Ethanol Precipitation. Antioxidants.

[587] Aleissa M.S., Alkahtani S., Abd Eldaim M.A., Ahmed A.M., Bungău S.G., Almutairi B., Bin-Jumah M., AlKahtane A.A., Alyousif M.S., Abdel-Daim M.M. (2020). Fucoidan Ameliorates Oxidative Stress, Inflammation, DNA Damage, and Hepatorenal Injuries in Diabetic Rats Intoxicated with Aflatoxin B1. Oxid. Med. Cell. Longev..

[588] Fernando I., Sanjeewa K., Lee H.G., Kim H.S., Vaas A., De Silva H., Nanayakkara C.M., Abeytunga D., Lee W.W., Lee D.S. (2020). Characterization and cytoprotective properties of Sargassum natans fucoidan against urban aerosol-induced keratinocyte damage. Int. J. Biol. Macromol..

[589] Zhang L.-L., Zhang L.-F., Xu J.-G., Hu Q.-P. (2017). Comparison study on antioxidant, DNA damage protective and antibacterial activities of eugenol and isoeugenol against several foodborne pathogens. Food Nutr. Res..

[590] Nam H., Kim M.-M. (2013). Eugenol with antioxidant activity inhibits MMP-9 related to metastasis in human fibrosarcoma cells. Food Chem. Toxicol. Int. J. Publ. Br. Ind. Biol. Res. Assoc..

[591] Kaur G., Athar M., Alam M.S. (2010). Eugenol precludes cutaneous chemical carcinogenesis in mouse by preventing oxidative stress and inflammation and by inducing apoptosis. Mol. Carcinog..

[592] Yogalakshmi B., Viswanathan P., Anuradha C.V. (2010). Investigation of antioxidant, anti-inflammatory and DNA-protective properties of eugenol in thioacetamide-induced liver injury in rats. Toxicology.

[593] Kar Mahapatra S., Chakraborty S.P., Majumdar S., Bag B.G., Roy S. (2009). Eugenol protects nicotine-induced superoxide mediated oxidative damage in murine peritoneal macrophages in vitro. Eur. J. Pharmacol..

[594] El-Ghor A.A., Noshy M.M., Galal A., Mohamed H.R.H. (2014). Normalization of nano-sized TiO2-induced clastogenicity, genotoxicity and mutagenicity by chlorophyllin administration in mice brain, liver, and bone marrow cells. Toxicol. Sci. Off. J. Soc. Toxicol..

[595] John K., Divi R.L., Keshava C., Orozco C.C., Schockley M.E., Richardson D.L., Poirier M.C., Nath J., Weston A. (2010). CYP1A1 and CYP1B1 gene expression and DNA adduct formation in normal human mammary epithelial cells exposed to benzo[a]pyrene in the absence or presence of chlorophyllin. Cancer Lett..

[596] Mauriz J.L., Molpeceres V., García-Mediavilla M.V., González P., Barrio J.P., González-Gallego J. (2007). Melatonin prevents oxidative stress and changes in antioxidant enzyme expression and activity in the liver of aging rats. J. Pineal Res..

[597] Kireev R.A., Tresguerres A.C., Castillo C., Salazar V., Ariznavarreta C., Vara E., Tresguerres J.A. (2007). Effect of exogenous administration of melatonin and growth hormone on pro-antioxidant functions of the liver in aging male rats. J. Pineal Res..

[598] Ortiz-Franco M., Planells E., Quintero B., Acuña-Castroviejo D., Rusanova I., Escames G., Molina-López J. (2017). Effect of Melatonin Supplementation on Antioxidant Status and DNA Damage in High Intensity Trained Athletes. Int. J. Sports Med..

[599] Tripathi D.N., Jena G.B. (2010). Effect of melatonin on the expression of Nrf2 and NF-kappaB during cyclophosphamide-induced urinary bladder injury in rat. J. Pineal Res..

[600] Shokrzadeh M., Ghassemi-Barghi N. (2018). Melatonin Loading Chitosan-Tripolyphosphate Nanoparticles: Application in Attenuating Etoposide-Induced Genotoxicity in HepG2 Cells. Pharmacology.

[601] Janjetovic Z., Jarrett S.G., Lee E.F., Duprey C., Reiter R.J., Slominski A.T. (2017). Melatonin and its metabolites protect human melanocytes against UVB-induced damage: Involvement of NRF2-mediated pathways. Sci. Rep..

[602] Li R., Luo X., Li L., Peng Q., Yang Y., Zhao L., Ma M., Hou Z. (2016). The Protective Effects of Melatonin against Oxidative Stress and Inflammation Induced by Acute Cadmium Exposure in Mice Testis. Biol. Trace Elem. Res..

[603] Fischer T.W., Kleszczyński K., Hardkop L.H., Kruse N., Zillikens D. (2013). Melatonin enhances antioxidative enzyme gene expression (CAT, GPx, SOD), prevents their UVR-induced depletion, and protects against the formation of DNA damage (8-hydroxy-2′-deoxyguanosine) in ex vivo human skin. J. Pineal Res..

[604] Wang J., Wang X., He Y., Jia L., Yang C.S., Reiter R.J., Zhang J. (2019). Antioxidant and Pro-Oxidant Activities of Melatonin in the Presence of Copper and Polyphenols In Vitro and In Vivo. Cells.

[605] Yazğan B., Yazğan Y., Övey İ.S., Nazıroğlu M. (2016). Raloxifene and Tamoxifen Reduce PARP Activity, Cytokine and Oxidative Stress Levels in the Brain and Blood of Ovariectomized Rats. J. Mol. Neurosci. MN.

[606] Turacli I.D., Candar T., Yuksel E.B., Kalay S., Oguz A.K., Demirtas S. (2018). Potential effects of metformin in DNA BER system based on oxidative status in type 2 diabetes. Biochimie.

[607] Maayah Z.H., Ghebeh H., Alhaider A.A., El-Kadi A.O., Soshilov A.A., Denison M.S., Ansari M.A., Korashy H.M. (2015). Metformin inhibits 7,12-dimethylbenz[a]anthracene-induced breast carcinogenesis and adduct formation in human breast cells by inhibiting the cytochrome P4501A1/aryl hydrocarbon receptor signaling pathway. Toxicol. Appl. Pharmacol..

[608] Nna V.U., Bakar A.B.A., Ahmad A., Mohamed M. (2019). Down-regulation of steroidogenesis-related genes and its accompanying fertility decline in streptozotocin-induced diabetic male rats: Ameliorative effect of metformin. Andrology.

[609] Park S.-K., Shin O.S. (2017). Metformin alleviates ageing cellular phenotypes in Hutchinson-Gilford progeria syndrome dermal fibroblasts. Exp. Dermatol..

[610] Xu G., Wu H., Zhang J., Li D., Wang Y., Wang Y., Zhang H., Lu L., Li C., Huang S. (2015). Metformin ameliorates ionizing irradiation-induced long-term hematopoietic stem cell injury in mice. Free. Radic. Biol. Med..

[611] Asensio-López M.C., Lax A., Pascual-Figal D.A., Valdés M., Sánchez-Más J. (2011). Metformin protects against doxorubicin-induced cardiotoxicity: Involvement of the adiponectin cardiac system. Free. Radic. Biol. Med..

[612] Qin D., Ren R., Jia C., Lu Y., Yang Q., Chen L., Wu X., Zhu J., Guo Y., Yang P. (2018). Rapamycin Protects Skin Fibroblasts from Ultraviolet B-Induced Photoaging by Suppressing the Production of Reactive Oxygen Species. Cell. Physiol. Biochem. Int. J. Exp. Cell. Physiol. Biochem. Pharmacol..

[613] Awad E., Othman E.M., Stopper H. (2017). Effects of Resveratrol, Lovastatin and the mTOR-Inhibitor RAD-001 on Insulin-Induced Genomic Damage In Vitro. Molecules.

[614] Hsu C.S., Li Y. (2002). Aspirin potently inhibits oxidative DNA strand breaks: Implications for cancer chemoprevention. Biochem. Biophys. Res. Commun..

[615] de S Moreira D., Figueiró P.W., Siebert C., Prezzi C.A., Rohden F., Guma F., Manfredini V., Wyse A. (2018). Chronic Mild Hyperhomocysteinemia Alters Inflammatory and Oxidative/Nitrative Status and Causes Protein/DNA Damage, as well as Ultrastructural Changes in Cerebral Cortex: Is Acetylsalicylic Acid Neuroprotective?. Neurotox. Res..

[616] Korkmaz-Icöz S., Atmanli A., Radovits T., Li S., Hegedüs P., Ruppert M., Brlecic P., Yoshikawa Y., Yasui H., Karck M. (2016). Administration of zinc complex of acetylsalicylic acid after the onset of myocardial injury protects the heart by upregulation of antioxidant enzymes. J. Physiol. Sci. JPS.

[617] Miller L., Shapiro A.M., Cheng J., Wells P.G. (2013). The free radical spin trapping agent phenylbutylnitrone reduces fetal brain DNA oxidation and postnatal cognitive deficits caused by in utero exposure to a non-structurally teratogenic dose of ethanol: A role for oxidative stress. Free. Radic. Biol. Med..

[618] Skolimowski J.J., Cieślińska B., Zak M., Osiecka R., Błaszczyk A. (2010). Modulation of ethoxyquin genotoxicity by free radical scavengers and DNA damage repair in human lymphocytes. Toxicol. Lett..

[619] Hirano H., Tabuchi Y., Kondo T., Zhao Q.L., Ogawa R., Cui Z.G., Feril L.B., Kanayama S. (2005). Analysis of gene expression in apoptosis of human lymphoma U937 cells induced by heat shock and the effects of alpha-phenyl N-tert-butylnitrone (PBN) and its derivatives. Apoptosis Int. J. Program. Cell Death.

[620] Błasiak J., Arabski M., Pertyński T., Małecka-Panas E., Woźniak K., Drzewoski J. (2002). DNA damage in human colonic mucosa cells evoked by nickel and protective action of quercetin—Involvement of free radicals?. Cell. Biol. Toxicol..

[621] Atamna H., Paler-Martínez A., Ames B.N. (2000). N-t-butyl hydroxylamine, a hydrolysis product of alpha-phenyl-N-t-butyl nitrone, is more potent in delaying senescence in human lung fibroblasts. J. Biol. Chem..

[622] Szeto Y.T., Benzie I.F., Collins A.R., Choi S.W., Cheng C.Y., Yow C.M., Tse M.M. (2005). A buccal cell model comet assay: Development and evaluation for human biomonitoring and nutritional studies. Mutat. Res..

[623] Martin L.J., Chen K., Liu Z. (2005). Adult motor neuron apoptosis is mediated by nitric oxide and Fas death receptor linked by DNA damage and p53 activation. J. Neurosci. Off. J. Soc. Neurosci..

[624] Laurent C., Pouget J.-P., Voisin P. (2005). Modulation of DNA damage by pentoxifylline and alpha-tocopherol in skin fibroblasts exposed to Gamma rays. Radiat. Res..

[625] Liao G., Li R., Chen X., Zhang W., Du S., Yuan Y. (2016). Sodium valproate prevents radiation-induced injury in hippocampal neurons via activation of the Nrf2/HO-1 pathway. Neuroscience.

[626] Tokarz P., Kaarniranta K., Blasiak J. (2016). Inhibition of DNA methyltransferase or histone deacetylase protects retinal pigment epithelial cells from DNA damage induced by oxidative stress by the stimulation of antioxidant enzymes. Eur. J. Pharmacol..

[627] Othman M.F.B., Mitry N.R., Lewington V.J., Blower P.J., Terry S.Y.A. (2017). Re-assessing gallium-67 as a therapeutic radionuclide. Nucl. Med. Biol..

[628] Čabarkapa A., Borozan S., Živković L., Stojanović S., Milanović-Čabarkapa M., Bajić V., Spremo-Potparević B. (2015). CaNa2EDTA chelation attenuates cell damage in workers exposed to lead—A pilot study. Chem. Interact..

[629] Čabarkapa A., Dekanski D., Živković L., Milanović-Čabarkapa M., Bajić V., Topalović D., Giampieri F., Gasparrini M., Battino M., Spremo-Potparević B. (2017). Unexpected effect of dry olive leaf extract on the level of DNA damage in lymphocytes of lead intoxicated workers, before and after CaNaEDTA chelation therapy. Food Chem. Toxicol. Int. J. Publ. Br. Ind. Biol. Res. Assoc.

[630] Ward W.M., Hoffman J.D., Loo G. (2015). Genotoxic effect of ethacrynic acid and impact of antioxidants. Toxicol. Appl. Pharmacol..

[631] Benadiba J., Rosilio C., Nebout M., Heimeroth V., Neffati Z., Popa A., Mary D., Griessinger E., Imbert V., Sirvent N. (2017). Iron chelation: An adjuvant therapy to target metabolism, growth and survival of murine PTEN-deficient T lymphoma and human T lymphoblastic leukemia/lymphoma. Leuk. Lymphoma.

[632] Kipp A.P. (2019). Selenium in colorectal and differentiated thyroid cancer. Hormones.

[633] Wimalawansa S.J. (2019). Vitamin D Deficiency: Effects on Oxidative Stress, Epigenetics, Gene Regulation, and Aging. Biology.

[634] Chen L., Yang R., Qiao W., Yuan X., Wang S., Goltzman D., Miao D. (2018). 1,25-Dihydroxy vitamin D prevents tumorigenesis by inhibiting oxidative stress and inducing tumor cellular senescence in mice. Int. J. Cancer.

[635] Bukhari S.A., Naqvi S.A.R., Nagra S.A., Anjum F., Javed S., Farooq M. (2015). Assessing of oxidative stress related parameters in diabetes mellitus type 2: Cause excessive damaging to DNA and enhanced homocysteine in diabetic patients. Pak. J. Pharm. Sci..

[636] Wong C.P., Magnusson K.R., Ho E. (2013). Increased inflammatory response in aged mice is associated with age-related zinc deficiency and zinc transporter dysregulation. J. Nutr. Biochem..

[637] Georgousopoulou E.N., Panagiotakos D.B., Mellor D.D., Naumovski N. (2017). Tocotrienols, health and ageing: A systematic review. Maturitas.

[638] Kiokias S., Proestos C., Oreopoulou V. (2018). Effect of Natural Food Antioxidants against LDL and DNA Oxidative Changes. Antioxidants.

[639] Zhai T., Li S., Hu W., Li D., Leng S. (2018). Potential Micronutrients and Phytochemicals against the Pathogenesis of Chronic Obstructive Pulmonary Disease and Lung Cancer. Nutrients.

[640] Senoner T., Dichtl W. (2019). Oxidative Stress in Cardiovascular Diseases: Still a Therapeutic Target?. Nutrients.

[641] Forni C., Facchiano F., Bartoli M., Pieretti S., Facchiano A., D’Arcangelo D., Norelli S., Valle G., Nisini R., Beninati S. (2019). Beneficial Role of Phytochemicals on Oxidative Stress and Age-Related Diseases. BioMed Res. Int..

[642] Griffiths K., Aggarwal B.B., Singh R.B., Buttar H.S., Wilson D., de Meester F. (2016). Food Antioxidants and Their Anti-Inflammatory Properties: A Potential Role in Cardiovascular Diseases and Cancer Prevention. Diseases.

[643] George V.C., Dellaire G., Rupasinghe H.P.V. (2017). Plant flavonoids in cancer chemoprevention: Role in genome stability. J. Nutr. Biochem..

[644] Azqueta A., Collins A. (2016). Polyphenols and DNA Damage: A Mixed Blessing. Nutrients.

[645] Pérez-Hernández J., Zaldívar-Machorro V.J., Villanueva-Porras D., Vega-Ávila E., Chavarría A. (2016). A Potential Alternative against Neurodegenerative Diseases: Phytodrugs. Oxidative Med. Cell. Longev..

[646] Qu G., Chen J., Guo X. (2019). The beneficial and deleterious role of dietary polyphenols on chronic degenerative diseases by regulating gene expression. Biosci. Trends.

[647] Galano A., Tan D.-X., Reiter R.J. (2018). Melatonin: A Versatile Protector against Oxidative DNA Damage. Molecules.

[648] Majidinia M., Sadeghpour A., Mehrzadi S., Reiter R.J., Khatami N., Yousefi B. (2017). Melatonin: A pleiotropic molecule that modulates DNA damage response and repair pathways. J. Pineal Res..

[649] Farhood B., Goradel N.H., Mortezaee K., Khanlarkhani N., Najafi M., Sahebkar A. (2019). Melatonin and cancer: From the promotion of genomic stability to use in cancer treatment. J. Cell. Physiol..

[650] Mok J.X., Ooi J.H., Ng K.Y., Koh R.Y., Chye S.M. (2019). A New Prospective on the Role of Melatonin in Diabetes and Its Complications. Horm. Mol. Biol. Clin. Investig..

[651] Baltatu O.C., Senar S., Campos L.A., Cipolla-Neto J. (2019). Cardioprotective Melatonin: Translating From Proof-of-Concept Studies to Therapeutic Use. Int. J. Mol. Sci..

[652] Imenshahidi M., Karimi G., Hosseinzadeh H. (2020). Effects of Melatonin on Cardiovascular Risk Factors and Metabolic Syndrome: A Comprehensive Review. Naunyn Schmiedebergs Arch. Pharmacol..

[653] Kassm S.A., Naja W., Hoertel N., Limosin F. (2019). Pharmacological Management of Delusions Associated With Dementia. Geriatr. Psychol. Neuropsychiatr. Vieil..

[654] Cardinali D.P. (2019). Melatonin: Clinical Perspectives in Neurodegeneration. Front. Endocrinol. (Lausanne).

[655] Pomatto L.C.D., Davies K.J.A. (2018). Adaptive homeostasis and the free radical theory of ageing. Free. Radic. Biol. Med..

[656] Bacanlı M., Aydın S., Başaran A.A., Başaran N. (2017). Are all phytochemicals useful in the preventing of DNA damage?. Food Chem. Toxicol. Int. J. Publ. Br. Ind. Biol. Res. Assoc..

[657] Sjakste N., Djelic N., Dzintare M., Zivkovic L. (2020). DNA-BINDING and DNA-protecting activities of small natural organic molecules and food extracts. Chem. Biol. Interact.

[658] Selman C., McLaren J.S., Meyer C., Duncan J.S., Redman P., Collins A.R., Duthie G.G., Speakman J.R. (2006). Life-long vitamin C supplementation in combination with cold exposure does not affect oxidative damage or lifespan in mice, but decreases expression of antioxidant protection genes. Mech. Ageing Dev..

[659] Miura K., Green A.C. (2015). Dietary Antioxidants and Melanoma: Evidence from Cohort and Intervention Studies. Nutr. Cancer.

[660] Mocchegiani E., Costarelli L., Giacconi R., Malavolta M., Basso A., Piacenza F., Ostan R., Cevenini E., Gonos E.S., Franceschi C. (2014). Vitamin E-gene interactions in aging and inflammatory age-related diseases: Implications for treatment. A systematic review. Ageing Res. Rev..

[661] Perron N.R., García C.R., Pinzón J.R., Chaur M.N., Brumaghim L.J. (2011). Antioxidant and prooxidant effects of polyphenol compounds on copper-mediated DNA damage. J. Inorg. Biochem..

[662] Romero A., Ramos E., de los Ríos C., Egea J., del Pino J., Reiter R.J. (2014). A review of metal-catalyzed molecular damage: Protection by melatonin. J. Pineal Res..

[663] Shaito A., Posadino A.M., Younes N., Hasan H., Halabi S., Alhababi D., Al-Mohannadi A., Abdel-Rahman W.M., Eid A.H., Nasrallah G.K. (2020). Potential Adverse Effects of Resveratrol: A Literature Review. Int. J. Mol. Sci..

[664] Das A., Majumder D., Saha C. (2017). Correlation of binding efficacies of DNA to flavonoids and their induced cellular damage. J. Photochem. Photobiol. B. Biol..

[665] Salehi B., Mishra A.P., Nigam M., Sener B., Kilic M., Sharifi-Rad M., Fokou P., Martins N., Sharifi-Rad J. (2018). Resveratrol: A Double-Edged Sword in Health Benefits. Biomedicines.

[666] Baur J.A., Sinclair D.A. (2006). Therapeutic potential of resveratrol: The in vivo evidence. Nat. Rev. Drug Discov..

[667] Jan A.T., Azam M., Siddiqui K., Ali A., Choi I., Haq Q.M.R. (2015). Heavy Metals and Human Health: Mechanistic Insight into Toxicity and Counter Defense System of Antioxidants. Int. J. Mol. Sci..

[668] Chen F., Tang Q., Ma H., Bian K., Seeram N.P., Li D. (2019). Hydrolyzable Tannins Are Iron Chelators That Inhibit DNA Repair Enzyme ALKBH2. Chem. Res. Toxicol..

[669] Sarwar T., Zafaryab M., Husain M.A., Ishqi H.M., Rehman S.U., Rizvi M.M., Tabish M. (2015). Redox cycling of endogenous copper by ferulic acid leads to cellular DNA breakage and consequent cell death: A putative cancer chemotherapy mechanism. Toxicol. Appl. Pharmacol..

[670] Mazidi M., Kengne A.-P., Banach M. (2017). Mineral and vitamin consumption and telomere length among adults in the United States. Pol. Arch. Intern. Med..

[671] Paul L., Cattaneo M., D’Angelo A., Sampietro F., Fermo I., Razzari C., Fontana G., Eugene N., Jacques P.F., Selhub J. (2009). Telomere length in peripheral blood mononuclear cells is associated with folate status in men. J. Nutr..

[672] Tucker L.A. (2019). Serum and Dietary Folate and Vitamin B12 Levels Account for Differences in Cellular Aging: Evidence Based on Telomere Findings in 5581 U.S. Adults. Oxidative Med. Cell. Longev..

[673] Milić M., Rozgaj R., Kašuba V., Oreščanin V., Balija M., Jukić I. (2010). Correlation between folate and vitamin B₁₂ and markers of DNA stability in healthy men: Preliminary results. Acta Biochim. Pol..

[674] Shirazi P.T., Leifert W.R., Fenech M.F., François M. (2018). Folate modulates guanine-quadruplex frequency and DNA damage in Werner syndrome. Mutat. Res. Genet. Toxicol. Environ. Mutagen..

[675] Erusalimsky J.D. (2020). Oxidative stress, telomeres and cellular senescence: What non-drug interventions might break the link?. Free. Radic. Biol. Med..

[676] Pineda-Pampliega J., Herrera-Duenas A., Mulder E., Aguirre J.I., Hofle U., Verhulst S. (2020). Antioxidant supplementation slows telomere shortening in free-living white stork chicks. Proc. Biol. Sci..

[677] Wai K.M., Umezaki M., Umemura M., Mar O., Watanabe C. (2020). Protective role of selenium in the shortening of telomere length in newborns induced by in utero heavy metal exposure. Environ. Res..

[678] Shu Y., Wu M., Yang S., Wang Y., Li H. (2020). Association of dietary selenium intake with telomere length in middle-aged and older adults. Clin. Nutr..

[679] Farahzadi R., Fathi E., Mesbah-Namin S.A., Zarghami N. (2017). Zinc sulfate contributes to promote telomere length extension via increasing telomerase gene expression, telomerase activity and change in the TERT gene promoter CpG island methylation status of human adipose-derived mesenchymal stem cells. PLoS ONE.

[680] Bagherpour B., Gharagozloo M., Moayedi B. (2009). The influence of iron loading and iron chelation on the proliferation and telomerase activity of human peripheral blood mononuclear cells. Iran. J. Immunol. IJI.

[681] Martin H., Uring-Lambert B., Adrian M., Lahlou A., Bonet A., Demougeot C., Devaux S., Laurant P., Richert L., Berthelot A. (2008). Effects of long-term dietary intake of magnesium on oxidative stress, apoptosis and ageing in rat liver. Magnes. Res..

[682] Killilea D.W., Ames N.B. (2008). Magnesium deficiency accelerates cellular senescence in cultured human fibroblasts. Proc. Natl. Acad. Sci. USA.

[683] Shah N.C., Shah G.J., Li Z., Jiang X.-C., Altura B.T., Altura B.M. (2014). Short-term magnesium deficiency downregulates telomerase, upregulates neutral sphingomyelinase and induces oxidative DNA damage in cardiovascular tissues: Relevance to atherogenesis, cardiovascular diseases and aging. Int. J. Clin. Exp. Med..

[684] Amano H., Chaudhury A., Rodriguez-Aguayo C., Lu L., Akhanov V., Catic A., Popov Y.V., Verdin E., Johnson H., Stossi F. (2019). Telomere Dysfunction Induces Sirtuin Repression that Drives Telomere-Dependent Disease. Cell Metab..

[685] Praveen G., Shalini T., Sivaprasad M., Reddy G.B. (2020). Relative Telomere Length and Mitochondrial DNA Copy Number Variation With Age: Association With Plasma Folate and Vitamin B12. Mitochondrion.

[686] Lee J.-Y., Shin C., Baik I. (2017). Longitudinal associations between micronutrient consumption and leukocyte telomere length. J. Hum. Nutr. Diet. Off. J. Br. Diet. Assoc..

[687] Zhang X., Wang Y., Zhao R., Hu X., Zhang B., Lv X., Guo Z., Zhang Z., Yuan J., Chu X. (2019). Folic Acid Supplementation Suppresses Sleep Deprivation-Induced Telomere Dysfunction and Senescence-Associated Secretory Phenotype (SASP). Oxid. Med. Cell. Longev..

[688] Sen A., Marsche G., Freudenberger P., Schallert M., Toeglhofer A.M., Nagl C., Schmidt R., Launer L.J., Schmidt H. (2014). Association between higher plasma lutein, zeaxanthin, and vitamin C concentrations and longer telomere length: Results of the Austrian Stroke Prevention Study. J. Am. Geriatr. Soc..

[689] Kim Y.Y., Ku S.Y., Huh Y., Liu H.C., Kim S.H., Choi Y.M., Moon S.Y. (2013). Anti-aging effects of vitamin C on human pluripotent stem cell-derived cardiomyocytes. Age.

[690] Vetter V.M., Spira D., Banszerus V.L., Demuth I. (2020). Epigenetic Clock and Leukocyte Telomere Length are Associated with Vitamin D Status, but not with Functional Assessments and Frailty in the Berlin Aging Study II. J. Gerontol Biol. Sci. Med. Sci..

[691] Farhangi M.A., Najafi M. (2020). The association between dietary quality indices and serum telomerase activity in patient candidates for CABG. Eat Weight Disord..

[692] Corina A., Rangel-Zúñiga O.A., Jiménez-Lucena R., Alcalá-Díaz J.F., Quintana-Navarro G., Yubero-Serrano E.M., López-Moreno J., Delgado-Lista J., Tinahones F., Ordovás J.M. (2019). Low Intake of Vitamin E Accelerates Cellular Aging in Patients With Established Cardiovascular Disease: The Cordioprev Study. J. Gerontol. Ser. A Biol. Sci. Med. Sci..

[693] Velichkovska M., Surnar B., Nair M., Dhar S., Toborek M. (2019). Targeted Mitochondrial COQ Delivery Attenuates Antiretroviral-Drug-Induced Senescence of Neural Progenitor Cells. Mol. Pharm..

[694] Aminizadeh N., Tiraihi T., Mesbah-Namin S.A., Taheri T. (2016). Stimulation of cell proliferation by glutathione monoethyl ester in aged bone marrow stromal cells is associated with the assistance of TERT gene expression and telomerase activity. Vitr. Cell. Dev. Biol. Anim..

[695] Shao L., Li Q.-H., Tan Z. (2004). L-carnosine reduces telomere damage and shortening rate in cultured normal fibroblasts. Biochem. Biophys. Res. Commun..

[696] Farahzadi R., Mesbah-Namin S.A., Zarghami N., Fathi E. (2016). L-carnitine Effectively Induces hTERT Gene Expression of Human Adipose Tissue-derived Mesenchymal Stem Cells Obtained from the Aged Subjects. Int. J. Stem. Cells.

[697] Farahzadi R., Fathi E., Mesbah-Namin S.A., Zarghami N. (2018). Anti-aging protective effect of L-carnitine as clinical agent in regenerative medicine through increasing telomerase activity and change in the hTERT promoter CpG island methylation status of adipose tissue-derived mesenchymal stem cells. Tissue Cell.

[698] Yang W., Zhang G., Jiang F., Zeng Y., Zou P., An H., Chen Q., Ling X., Han F., Liu W. (2019). BPDE and B[a]P induce mitochondrial compromise by ROS-mediated suppression of the SIRT1/TERT/PGC-1α pathway in spermatogenic cells both in vitro and in vivo. Toxicol. Appl. Pharmacol..

[699] Ludlow A.T., Spangenburg E.E., Chin E.R., Cheng W.-H., Roth S.M. (2014). Telomeres shorten in response to oxidative stress in mouse skeletal muscle fibers. J. Gerontol. Ser. A Biol. Sci. Med. Sci..

[700] Voghel G., Thorin-Trescases N., Farhat N., Mamarbachi A.M., Villeneuve L., Fortier A., Perrault L.P., Carrier M., Thorin E. (2008). Chronic treatment with N-acetyl-cystein delays cellular senescence in endothelial cells isolated from a subgroup of atherosclerotic patients. Mech. Ageing Dev..

[701] Liu J., Liu M., Ye X., Liu K., Huang J., Wang L., Ji G., Liu N., Tang X., Baltz J.M. (2012). Delay in oocyte aging in mice by the antioxidant N-acetyl-L-cysteine (NAC). Hum. Reprod..

[702] Xu J., Li H., Yang K., Guo S., Wang J., Feng C., Chen H. (2019). Hyper-osmolarity environment-induced oxidative stress injury promotes nucleus pulposus cell senescence in vitro. Biosci. Rep..

[703] Sheng R., Gu Z., Xie M., Zhou W., Guo C. (2010). Epigallocatechin gallate protects H9c2 cardiomyoblasts against hydrogen dioxides- induced apoptosis and telomere attrition. Eur. J. Pharmacol..

[704] Sheng R., Gu Z.-L., Xie M.-L. (2013). Epigallocatechin gallate, the major component of polyphenols in green tea, inhibits telomere attrition mediated cardiomyocyte apoptosis in cardiac hypertrophy. Int. J. Cardiol..

[705] Maida H., Sanin H., Anja H., Naida L.K., Borivoj G., Ramic J., Lejla P. (2019). Bioflavonoids protect cells against halogenated boroxine-induced genotoxic damage by upregulation of hTERT expression. Z. Naturforsch. C J. Biosci..

[706] Tawani A., Kumar A. (2015). Structural Insight into the interaction of Flavonoids with Human Telomeric Sequence. Sci. Rep..

[707] Pattanayak R., Basak P., Sen S., Bhattacharyya M. (2016). Interaction of KRAS G-quadruplex with natural polyphenols: A spectroscopic analysis with molecular modeling. Int. J. Biol. Macromol..

[708] Pirmoradi S., Fathi E., Farahzadi R., Pilehvar-Soltanahmadi Y., Zarghami N. (2018). Curcumin Affects Adipose Tissue-Derived Mesenchymal Stem Cell Aging Through TERT Gene Expression. Drug Res..

[709] Xiao Z., Zhang A., Lin J., Zheng Z., Shi X., Di W., Qi W., Zhu Y., Zhou G., Fang Y. (2014). Telomerase: A target for therapeutic effects of curcumin and a curcumin derivative in Aβ1-42 insult in vitro. PLoS ONE.

[710] Jahan-Abad A.J., Morteza-Zadeh P., Negah S.S., Gorji A. (2017). Curcumin attenuates harmful effects of arsenic on neural stem/progenitor cells. Avicenna J. Phytomed..

[711] Selim A.M., Nooh M.M., El-Sawalhi M.M., Ismail N.A. (2020). Amelioration of age-related alterations in rat liver: Effects of curcumin C3 complex, Astragalus membranaceus and blueberry. Exp. Gerontol..

[712] Vinnarasi S., Radhika R., Vijayakumar S., Shankar R. (2019). Structural insights into the anti-cancer activity of quercetin on G-tetrad, mixed G-tetrad, and G-quadruplex DNA using quantum chemical and molecular dynamics simulations. J. Biomol. Struct. Dyn..

[713] Sengupta B., Pahari B., Blackmon L., Sengupta P.K. (2013). Prospect of bioflavonoid fisetin as a quadruplex DNA ligand: A biophysical approach. PLoS ONE.

[714] Parzonko A., Naruszewicz M. (2010). Silymarin inhibits endothelial progenitor cells’ senescence and protects against the antiproliferative activity of rapamycin: Preliminary study. J. Cardiovasc. Pharmacol..

[715] Jin Y., Li H., Liu P. (2010). Label-free electrochemical selection of G-quadruplex-binding ligands based on structure switching. Biosens. Bioelectron..

[716] Thomas P., Wang Y.J., Zhong J.H., Kosaraju S., O’Callaghan N.J., Zhou X.F., Fenech M. (2009). Grape seed polyphenols and curcumin reduce genomic instability events in a transgenic mouse model for Alzheimer’s disease. Mutat. Res..

[717] Liu M., Yin Y., Ye X., Zeng M., Zhao Q., Keefe D.L., Liu L. (2013). Resveratrol protects against age-associated infertility in mice. Hum. Reprod..

[718] Navarro S., Reddy R., Lee J., Warburton D., Driscoll B. (2017). Inhaled resveratrol treatments slow ageing-related degenerative changes in mouse lung. Thorax.

[719] Sodagam L., Lewinska A., Kwasniewicz E., Kokhanovska S., Wnuk M., Siems K., Rattan S. (2019). Phytochemicals Rosmarinic Acid, Ampelopsin, and Amorfrutin-A Can Modulate Age-Related Phenotype of Serially Passaged Human Skin Fibroblasts. Front. Genet..

[720] Tsoukalas D., Fragkiadaki P., Docea A.O., Alegakis A.K., Sarandi E., Thanasoula M., Spandidos D.A., Tsatsakis A., Razgonova M.P., Calina D. (2019). Discovery of potent telomerase activators: Unfolding new therapeutic and anti-aging perspectives. Mol. Med. Rep..

[721] Shi A.-W., Gu N., Liu X.-M., Wang X., Peng Y.-Z. (2011). Ginsenoside Rg1 enhances endothelial progenitor cell angiogenic potency and prevents senescence in vitro. J. Int. Med. Res..

[722] Zhou Y., Liu J., Cai S., Liu D., Jiang R., Wang Y. (2015). Protective effects of ginsenoside Rg1 on aging Sca-1+ hematopoietic cells. Mol. Med. Rep..

[723] Zhu J., Mu X., Zeng J., Xu C., Liu J., Zhang M., Li C., Chen J., Li T., Wang Y. (2014). Ginsenoside Rg1 prevents cognitive impairment and hippocampus senescence in a rat model of D-galactose-induced aging. PLoS ONE.

[724] Yung L.Y., Lam W.S., Ho M.K., Hu Y., Ip F.C., Pang H., Chin A.C., Harley C.B., Ip N.Y., Wong Y.H. (2012). Astragaloside IV and cycloastragenol stimulate the phosphorylation of extracellular signal-regulated protein kinase in multiple cell types. Planta Med..

[725] Liu J., Gao D., Dan J., Liu D., Peng L., Zhou R., Luo Y. (2019). The protective effect of cycloastragenol on aging mouse circadian rhythmic disorder induced by d-galactose. J. Cell. Biochem..

[726] Ip F.C., Ng Y.P., An H.J., Dai Y., Pang H.H., Hu Y.Q., Chin A.C., Harley C.B., Wong Y.H., Ip N.Y. (2014). Cycloastragenol is a potent telomerase activator in neuronal cells: Implications for depression management. Neurosignals.

[727] Mendelsohn A.R., Larrick J.W. (2015). Telomerase Reverse Transcriptase and Peroxisome Proliferator-Activated Receptor γ Co-Activator-1α Cooperate to Protect Cells from DNA Damage and Mitochondrial Dysfunction in Vascular Senescence. Rejuvenation Res..

[728] Rastmanesh R. (2011). Potential of melatonin to treat or prevent age-related macular degeneration through stimulation of telomerase activity. Med. Hypotheses.

[729] Akbulut K.G., Gonul B., Akbulut H. (2009). The role of melatonin on gastric mucosal cell proliferation and telomerase activity in ageing. J. Pineal Res..

[730] Yang L., Liu X., Song L., Su G., Di A., Bai C., Wei Z., Li G. (2019). Inhibiting repressive epigenetic modification promotes telomere rejuvenation in somatic cell reprogramming. FASEB J..

[731] Endo M., Kimura K., Kuwayama T., Monji Y., Iwata H. (2014). Effect of estradiol during culture of bovine oocyte-granulosa cell complexes on the mitochondrial DNA copies of oocytes and telomere length of granulosa cells. Zygote.

[732] Kokubun T., Saitoh S.-I., Miura S., Ishida T., Takeishi Y. (2019). Telomerase Plays a Pivotal Role in Collateral Growth Under Ischemia by Suppressing Age-Induced Oxidative Stress, Expression of p53, and Pro-Apoptotic Proteins. Int. Heart J..

[733] de Jesus B.B., Schneeberger K., Vera E., Tejera A., Harley C.B., Blasco M.A. (2011). The telomerase activator TA-65 elongates short telomeres and increases health span of adult/old mice without increasing cancer incidence. Aging Cell.

[734] Oh Y.S., Jeong S.-G., Cho G.-W. (2015). Anti-senescence effects of DNA methyltransferase inhibitor RG108 in human bone marrow mesenchymal stromal cells. Biotechnol. Appl. Biochem..

[735] Bikkul M.U., Clements C.S., Godwin L.S., Goldberg M.W., Kill I.R., Bridger J.M. (2018). Farnesyltransferase inhibitor and rapamycin correct aberrant genome organisation and decrease DNA damage respectively, in Hutchinson-Gilford progeria syndrome fibroblasts. Biogerontology.

[736] de Kreutzenberg S.V., Ceolotto G., Cattelan A., Pagnin E., Mazzucato M., Garagnani P., Borelli V., Bacalini M.G., Franceschi C., Fadini G.P. (2015). Metformin improves putative longevity effectors in peripheral mononuclear cells from subjects with prediabetes. A randomized controlled trial. Nutr. Metab. Cardiovasc. Dis..

[737] Chebel A., Catallo R., Mabon C., Bachy E., Wenner T., Salles G., Pouteil-Noble C., Ffrench M. (2016). Rapamycin safeguards lymphocytes from DNA damage accumulation in vivo. Eur. J. Cell Biol..

[738] Sodagam L., Lewinska A., Wnuk M., Rattan S.I.S. (2017). Chronic exposure to rapamycin and episodic serum starvation modulate ageing of human fibroblasts in vitro. Biogerontology.

[739] Pospelova T.V., Bykova T.V., Zubova S.G., Katolikova N.V., Yartzeva N.M., Pospelov V.A. (2013). Rapamycin induces pluripotent genes associated with avoidance of replicative senescence. Cell Cycle.

[740] Ferrara-Romeo I., Martinez P., Saraswati S., Whittemore K., Graña-Castro O., Thelma Poluha L., Serrano R., Hernandez-Encinas E., Blanco-Aparicio C., Maria Flores J. (2020). The mTOR Pathway Is Necessary for Survival of Mice With Short Telomeres. Nat. Commun..

[741] Chen C., Akiyama K., Yamaza T., You Y.O., Xu X., Li B., Zhao Y., Shi S. (2014). Telomerase governs immunomodulatory properties of mesenchymal stem cells by regulating FAS ligand expression. EMBO Mol. Med..

[742] Bär C., Huber N., Beier F., Blasco M.A. (2015). Therapeutic effect of androgen therapy in a mouse model of aplastic anemia produced by short telomeres. Haematologica.

[743] Townsley D.M., Dumitriu B., Liu D., Biancotto A., Weinstein B., Chen C., Hardy N., Mihalek A.D., Lingala S., Kim Y.J. (2016). Danazol Treatment for Telomere Diseases. N. Engl. J. Med..

[744] Ramunas J., Yakubov E., Brady J.J., Corbel S.Y., Holbrook C., Brandt M., Stein J., Santiago J.G., Cooke J.P., Blau H.M. (2015). Transient delivery of modified mRNA encoding TERT rapidly extends telomeres in human cells. FASEB J. Off. Publ. Fed. Am. Soc. Exp. Biol..

[745] Miwa S., Czapiewski R., Wan T., Bell A., Hill K.N., von Zglinicki T., Saretzki G. (2016). Decreased mTOR signalling reduces mitochondrial ROS in brain via accumulation of the telomerase protein TERT within mitochondria. Aging.

[746] Gensous N., Franceschi C., Santoro A., Milazzo M., Garagnani P., Bacalini M.G. (2019). The Impact of Caloric Restriction on the Epigenetic Signatures of Aging. Int. J. Mol. Sci..

[747] Ghosh S., Sinha J.K., Raghunath M. (2016). Epigenomic maintenance through dietary intervention can facilitate DNA repair process to slow down the progress of premature aging. IUBMB Life.

[748] Evans L.W., Stratton M.S., Ferguson B.S. (2020). Dietary natural products as epigenetic modifiers in aging-associated inflammation and disease. Nat. Prod. Rep..

[749] Pasyukova E.G., Vaiserman A.M. (2017). HDAC inhibitors: A new promising drug class in anti-aging research. Mech. Ageing Dev..

[750] Chang L.-C., Yu Y.-L. (2016). Dietary components as epigenetic-regulating agents against cancer. BioMedicine.

[751] Speckmann B., Schulz S., Hiller F., Hesse D., Schumacher F., Kleuser B., Geisel J., Obeid R., Grune T., Kipp A.P. (2017). Selenium increases hepatic DNA methylation and modulates one-carbon metabolism in the liver of mice. J. Nutr. Biochem..

[752] Zhang Q., Zheng S., Wang S., Jiang Z., Xu S. (2019). The Effects of Low Selenium on DNA Methylation in the Tissues of Chickens. Boil. Trace Elem. Res..

[753] Khalkar P., Ali H.A., Codó P., Argelich N.D., Martikainen A., Arzenani M.K., Lehmann S., Walfridsson J., Ungerstedt J., Fernandes A.P. (2018). Selenite and methylseleninic acid epigenetically affects distinct gene sets in myeloid leukemia: A genome wide epigenetic analysis. Free. Radic. Biol. Med..

[754] Yang X., Lv Y., Huang K., Luo Y., Xu W. (2016). Zinc inhibits aflatoxin B1-induced cytotoxicity and genotoxicity in human hepatocytes (HepG2 cells). Food Chem. Toxicol. Int. J. Publ. Br. Ind. Biol. Res. Assoc..

[755] Zhu Y., Liao X., Lu L., Li W., Zhang L., Ji C., Lin X., Liu H.C., Odle J., Luo X. (2017). Maternal dietary zinc supplementation enhances the epigenetic-activated antioxidant ability of chick embryos from maternal normal and high temperatures. Oncotarget.

[756] Khadivi F., Razavi S., Hashemi F. (2020). Protective effects of zinc on rat sperm chromatin integrity involvement: DNA methylation, DNA fragmentation, ubiquitination and protamination after bleomycin etoposide and cis-platin treatment. Theriogenology.

[757] Gallagher P.S., Larkin M., Thillainadesan G., Dhakshnamoorthy J., Balachandran V., Xiao H., Wellman C., Chatterjee R., Wheeler D., Grewal S. (2018). Iron homeostasis regulates facultative heterochromatin assembly in adaptive genome control. Nat. Struct. Mol. Biol..

[758] Lio C.J., Yue X., Lopez-Moyado I.F., Tahiliani M., Aravind L., Rao A. (2020). TET methylcytosine oxidases: New insights from a decade of research. J. Biosci..

[759] Nishikawa J., Ohyama T. (2013). Selective association between nucleosomes with identical DNA sequences. Nucleic Acids Res..

[760] Mishima Y., Jayasinghe C.D., Lu K., Otani J., Shirakawa M., Kawakami T., Kimura H., Hojo H., Carlton P., Tajima S. (2015). Nucleosome compaction facilitates HP1γ binding to methylated H3K9. Nucleic Acids Res..

[761] Parraguez M., Gajardo G. (2017). Variation of the interphase heterochromatin in Artemia (Crustacea, Anostraca) of the Americas is related to changes in nuclear size and ionic composition of hipersaline habitats. Braz. J. Biol. Rev. Brasleira Biol..

[762] Guo Z., Zhang Z., Wang Q., Zhang J., Wang L., Zhang Q., Li H., Wu S. (2018). Manganese chloride induces histone acetylation changes in neuronal cells: Its role in manganese-induced damage. Neurotoxicology.

[763] Passador J., Toffoli L.V., Fernandes K.B., Neves-Souza R.D., Pelosi G.G., Gomes M.V. (2018). Dietary Ingestion of Calories and Micronutrients Modulates the DNA Methylation Profile of Leukocytes from Older Individuals. J. Nutr. Health Aging.

[764] Arreguín A., Ribot J., Mušinović H., von Lintig J., Palou A., Bonet M.L. (2018). Dietary vitamin A impacts DNA methylation patterns of adipogenesis-related genes in suckling rats. Arch. Biochem. Biophys..

[765] Yan H.C., Li L., Liu J.C., Wang Y.F., Liu X.L., Ge W., Dyce P.W., Li L., Sun X.F., Shen W. (2019). RA promotes proliferation of primordial germ cell-like cells differentiated from porcine skin-derived stem cells. J. Cell. Physiol..

[766] Kumar S., Duester G. (2014). Retinoic acid controls body axis extension by directly repressing Fgf8 transcription. Development.

[767] Smith J.A., Ndoye A.M.N., Geary K., Lisanti M.P., Igoucheva O., Daniel R. (2010). A role for the Werner syndrome protein in epigenetic inactivation of the pluripotency factor Oct4. Aging Cell.

[768] Adhikary S., Sanyal S., Basu M., Sengupta I., Sen S., Srivastava D.K., Roy S., Das C. (2016). Selective Recognition of H3.1K36 Dimethylation/H4K16 Acetylation Facilitates the Regulation of All-trans-retinoic Acid (ATRA)-responsive Genes by Putative Chromatin Reader ZMYND8. J. Biol. Chem..

[769] Campuzano-García A.E., Torres-Alvarez B., Hernández-Blanco D., Fuentes-Ahumada C., Cortés-García J.D., Castanedo-Cázares J.P. (2019). DNA Methyltransferases in Malar Melasma and Their Modification by Sunscreen in Combination with 4% Niacinamide, 0.05% Retinoic Acid, or Placebo. BioMed Res. Int..

[770] Wu C.-Y., Feng X., Wei L.-N. (2014). Coordinated repressive chromatin-remodeling of Oct4 and Nanog genes in RA-induced differentiation of embryonic stem cells involves RIP140. Nucleic Acids Res..

[771] Chuang Y.S., Huang W.H., Park S.W., Persaud S.D., Hung C.H., Ho P.C., Wei L.N. (2011). Promyelocytic leukemia protein in retinoic acid-induced chromatin remodeling of Oct4 gene promoter. Stem Cells.

[772] Pellegrini C., Columbaro M., Capanni C., D’Apice M.R., Cavallo C., Murdocca M., Lattanzi G., Squarzoni S. (2015). All-trans retinoic acid and rapamycin normalize Hutchinson Gilford progeria fibroblast phenotype. Oncotarget.

[773] Lo Cicero A., Jaskowiak A.L., Egesipe A.L., Tournois J., Brinon B., Pitrez P.R., Ferreira L., de Sandre-Giovannoli A., Levy N., Nissan X. (2016). A High Throughput Phenotypic Screening reveals compounds that counteract premature osteogenic differentiation of HGPS iPS-derived mesenchymal stem cells. Sci. Rep..

[774] Zuo Q., Jin J., Jin K., Sun C., Song J., Zhang Y., Chen G., Li B. (2018). Distinct roles of retinoic acid and BMP4 pathways in the formation of chicken primordial germ cells and spermatogonial stem cells. Food Funct..

[775] Hou Y., Lautrup S., Cordonnier S., Wang Y., Croteau D.L., Zavala E., Zhang Y., Moritoh K., O’Connell J.F., Baptiste B.A. (2018). NAD supplementation normalizes key Alzheimer’s features and DNA damage responses in a new AD mouse model with introduced DNA repair deficiency. Proc. Natl. Acad. Sci. USA.

[776] Choy J.S., Qadri B., Henry L., Shroff K., Bifarin O., Basrai M.A. (2015). A Genome-Wide Screen with Nicotinamide to Identify Sirtuin-Dependent Pathways in Saccharomyces cerevisiae. G3.

[777] Libri V., Yandim C., Athanasopoulos S., Loyse N., Natisvili T., Law P.P., Chan P.K., Mohammad T., Mauri M., Tam K.T. (2014). Epigenetic and neurological effects and safety of high-dose nicotinamide in patients with Friedreich’s ataxia: An exploratory, open-label, dose-escalation study. Lancet.

[778] Chan P.K., Torres R., Yandim C., Law P.P., Khadayate S., Mauri M., Grosan C., Chapman-Rothe N., Giunti P., Pook M. (2013). Heterochromatinization induced by GAA-repeat hyperexpansion in Friedreich’s ataxia can be reduced upon HDAC inhibition by vitamin B3. Hum. Mol. Genet..

[779] Kiss T., Giles C.B., Tarantini S., Yabluchanskiy A., Balasubramanian P., Gautam T., Csipo T., Nyúl-Tóth Á., Lipecz A., Szabo C. (2019). Nicotinamide mononucleotide (NMN) supplementation promotes anti-aging miRNA expression profile in the aorta of aged mice, predicting epigenetic rejuvenation and anti-atherogenic effects. Geroscience.

[780] Sae-Lee C., Corsi S., Barrow T.M., Kuhnle G., Bollati V., Mathers J.C., Byun H.M. (2018). Dietary Intervention Modifies DNA Methylation Age Assessed by the Epigenetic Clock. Mol. Nutr. Food Res..

[781] Pirouzpanah S., Taleban F.-A., Mehdipour P., Atri M. (2015). Association of folate and other one-carbon related nutrients with hypermethylation status and expression of RARB, BRCA1, and RASSF1A genes in breast cancer patients. J. Mol. Med..

[782] Starczak M., Zarakowska E., Modrzejewska M., Dziaman T., Szpila A., Linowiecka K., Guz J., Szpotan J., Gawronski M., Labejszo A. (2018). In vivo evidence of ascorbate involvement in the generation of epigenetic DNA modifications in leukocytes from patients with colorectal carcinoma, benign adenoma and inflammatory bowel disease. J. Transl. Med..

[783] Tang Y., Luo M., Pan K., Ahmad T., Zhou T., Miao Z., Zhou H., Sun H., Xu X., Namaka M. (2019). DNA hydroxymethylation changes in response to spinal cord damage in a multiple sclerosis mouse model. Epigenomics.

[784] Qu Y.N., Zhang L., Wang T., Zhang H.Y., Yang Z.J., Yuan F.F., Wang Y., Li S.W., Jiang X.X., Xie X.H. (2020). Vitamin C Treatment Rescues Prelamin A-Induced Premature Senescence of Subchondral Bone Mesenchymal Stem Cells. Stem Cells Int..

[785] Gillberg L., Ørskov A.D., Nasif A., Ohtani H., Madaj Z., Hansen J.W., Rapin N., Mogensen J.B., Liu M., Dufva I.H. (2019). Oral vitamin C supplementation to patients with myeloid cancer on azacitidine treatment: Normalization of plasma vitamin C induces epigenetic changes. Clin. Epigenet..

[786] Chen L., Dong Y., Bhagatwala J., Raed A., Huang Y., Zhu H. (2019). Effects of Vitamin D3 Supplementation on Epigenetic Aging in Overweight and Obese African Americans With Suboptimal Vitamin D Status: A Randomized Clinical Trial. J. Gerontol. Ser. A Biol. Sci. Med. Sci..

[787] Castellano-Castillo D., Morcillo S., Crujeiras A.B., Sánchez-Alcoholado L., Clemente-Postigo M., Torres E., Tinahones F.J., Macias-Gonzalez M. (2019). Association between serum 25-hydroxyvitamin D and global DNA methylation in visceral adipose tissue from colorectal cancer patients. BMC Cancer.

[788] Mostafa A., Jalilvand S., Shoja Z., Nejati A., Shahmahmoodi S., Sahraian M.A., Marashi S.M. (2017). Multiple sclerosis-associated retrovirus, Epstein-Barr virus, and vitamin D status in patients with relapsing remitting multiple sclerosis. J. Med. Virol..

[789] Kreienkamp R., Croke M., Neumann M.A., Bedia-Diaz G., Graziano S., Dusso A., Dorsett D., Carlberg C., Gonzalo S. (2016). Vitamin D receptor signaling improves Hutchinson-Gilford progeria syndrome cellular phenotypes. Oncotarget.

[790] Lai G.R., Lee Y.F., Yan S.J., Ting H.J. (2018). Active vitamin D induces gene-specific hypomethylation in prostate cancer cells developing vitamin D resistance. Am. J. Physiol. Cell Physiol..

[791] Zappe K., Pointner A., Switzeny O.J., Magnet U., Tomeva E., Heller J., Mare G., Wagner K.H., Knasmueller S., Haslberger A.G. (2018). Counteraction of Oxidative Stress by Vitamin E Affects Epigenetic Regulation by Increasing Global Methylation and Gene Expression of and Dose Dependently in Caco-2 Cells. Oxidative Med. Cell. Longev..

[792] Remely M., Ferk F., Sterneder S., Setayesh T., Kepcija T., Roth S., Noorizadeh R., Greunz M., Rebhan I., Wagner K.H. (2017). Vitamin E Modifies High-Fat Diet-Induced Increase of DNA Strand Breaks, and Changes in Expression and DNA Methylation of Dnmt1 and MLH1 in C57BL/6J Male Mice. Nutrients.

[793] Knock E., Deng L., Wu Q., Lawrance A.K., Wang X., Rozen R. (2008). Strain differences in mice highlight the role of DNA damage in neoplasia induced by low dietary folate. J. Nutr..

[794] Kharbanda K.K., Rogers D.D., Mailliard M.E., Siford G.L., Barak A.J., Beckenhauer H.C., Sorrell M.F., Tuma D.J. (2005). Role of elevated S-adenosylhomocysteine in rat hepatocyte apoptosis: Protection by betaine. Biochem. Pharmacol..

[795] Chatterjee N., Yang J., Yoon D., Kim S., Joo S.-W., Choi J. (2017). Differential crosstalk between global DNA methylation and metabolomics associated with cell type specific stress response by pristine and functionalized MWCNT. Biomaterials.

[796] Takumi S., Okamura K., Yanagisawa H., Sano T., Kobayashi Y., Nohara K. (2015). The effect of a methyl-deficient diet on the global DNA methylation and the DNA methylation regulatory pathways. J. Appl. Toxicol. JAT.

[797] Huang D., Zhang Y., Qi Y., Chen C., Ji W. (2008). Global DNA hypomethylation, rather than reactive oxygen species (ROS), a potential facilitator of cadmium-stimulated K562 cell proliferation. Toxicol. Lett..

[798] Muteliefu G., Shimizu H., Enomoto A., Nishijima F., Takahashi M., Niwa T. (2012). Indoxyl sulfate promotes vascular smooth muscle cell senescence with upregulation of p53, p21, and prelamin A through oxidative stress. Am. J. Physiol. Cell Physiol..

[799] Rabaça A., Ferreira C., Bernardino R., Alves M., Oliveira P., Viana P., Barros A., Sousa M., Sá R. (2020). Use of antioxidant could ameliorate the negative impact of etoposide on human sperm DNA during chemotherapy. Reprod. Biomed. Online.

[800] Oyama J.I., Shiraki A., Nishikido T., Maeda T., Komoda H., Shimizu T., Makino N., Node K. (2017). EGCG, a green tea catechin, attenuates the progression of heart failure induced by the heart/muscle-specific deletion of MnSOD in mice. J. Cardiol..

[801] Niu Y., Na L., Feng R., Gong L., Zhao Y., Li Q., Li Y., Sun C. (2013). The phytochemical, EGCG, extends lifespan by reducing liver and kidney function damage and improving age-associated inflammation and oxidative stress in healthy rats. Aging Cell.

[802] Remely M., Ferk F., Sterneder S., Setayesh T., Roth S., Kepcija T., Noorizadeh R., Rebhan I., Greunz M., Beckmann J. (2017). EGCG Prevents High Fat Diet-Induced Changes in Gut Microbiota, Decreases of DNA Strand Breaks, and Changes in Expression and DNA Methylation of and in C57BL/6J Male Mice. Oxidative Med. Cell. Longev..

[803] Xie C.-R., You C.-G., Zhang N., Sheng H.-S., Zheng X.-S. (2018). Epigallocatechin Gallate Preferentially Inhibits O6-Methylguanine DNA-Methyltransferase Expression in Glioblastoma Cells Rather than in Nontumor *Glial Cells*. Nutr. Cancer.

[804] Ciesielski O., Biesiekierska M., Balcerczyk A. (2020). Epigallocatechin-3-gallate (EGCG) Alters Histone Acetylation and Methylation and Impacts Chromatin Architecture Profile in Human Endothelial Cells. Molecules.

[805] Kim A., Yun J.-M. (2017). Combination Treatments with Luteolin and Fisetin Enhance Anti-Inflammatory Effects in High Glucose-Treated THP-1 Cells Through Histone Acetyltransferase/Histone Deacetylase Regulation. J. Med. Food.

[806] Babangida S., Ibrahim S., Muhammad A., Arthur D.E., Uzairu A., Garba A. (2018). The role of molecular modelling strategies in validating the effects of chrysin on sodium arsenite-induced chromosomal and DNA damage. Hum. Exp. Toxicol..

[807] Doğan H.O., Alçiğir M.E. (2019). Assessment of epigenetic changes and oxidative DNA damage in rat pups exposed to polychlorinated biphenyls and the protective effect of curcumin in the prenatal period. J. Basic Clin. Physiol. Pharmacol..

[808] Bunker S.K., Dutta A., Pradhan J., Dandapat J., Chainy G.B.N. (2019). Curcumin restores hepatic epigenetic changes in propylthiouracil(PTU)Induced hypothyroid male rats: A study on DNMTs, MBDs, GADD45a, C/EBP-β and PCNA. Food Chem. Toxicol. Int. J. Publ. Br. Ind. Biol. Res. Assoc..

[809] Wang S.H., Lin P.Y., Chiu Y.C., Huang J.S., Kuo Y.T., Wu J.C., Chen C.C. (2015). Curcumin-Mediated HDAC Inhibition Suppresses the DNA Damage Response and Contributes to Increased DNA Damage Sensitivity. PLoS ONE.

[810] Tillhon M., Cazzalini O., Nardo T., Necchi D., Sommatis S., Stivala L.A., Scovassi A.I., Prosperi E. (2012). p300/CBP acetyl transferases interact with and acetylate the nucleotide excision repair factor XPG. DNA Repair.

[811] Link A., Balaguer F., Shen Y., Lozano J.J., Leung H.C., Boland C.R., Goel A. (2013). Curcumin modulates DNA methylation in colorectal cancer cells. PLoS ONE.

[812] Sebastià N., Montoro A., Hervás D., Pantelias G., Hatzi V.I., Soriano J.M., Villaescusa J.I., Terzoudi G.I. (2014). Curcumin and trans-resveratrol exert cell cycle-dependent radioprotective or radiosensitizing effects as elucidated by the PCC and G2-assay. Mutat. Res..

[813] Grabowska W., Mosieniak G., Achtabowska N., Czochara R., Litwinienko G., Bojko A., Sikora E., Bielak-Zmijewska A. (2019). Curcumin induces multiple signaling pathways leading to vascular smooth muscle cell senescence. Biogerontology.

[814] Al-Yousef N., Shinwari Z., Al-Shahrani B., Al-Showimi M., Al-Moghrabi N. (2020). Curcumin induces reexpression of BRCA1 and suppression of gamma synuclein by modulating DNA promoter methylation in breast cancer cell lines. Oncol. Rep..

[815] Okawara M., Katsuki H., Kurimoto E., Shibata H., Kume T., Akaike A. (2007). Resveratrol protects dopaminergic neurons in midbrain slice culture from multiple insults. Biochem. Pharmacol..

[816] Bishayee K., Khuda-Bukhsh A.R., Huh S.-O. (2015). PLGA-Loaded Gold-Nanoparticles Precipitated with Quercetin Downregulate HDAC-Akt Activities Controlling Proliferation and Activate p53-ROS Crosstalk to Induce Apoptosis in Hepatocarcinoma *Cells*. Mol. Cells.

[817] Geng L., Liu Z., Zhang W., Li W., Wu Z., Wang W., Ren R., Su Y., Wang P., Sun L. (2019). Chemical screen identifies a geroprotective role of quercetin in premature aging. Protein Cell.

[818] Lee K.S., Cha H.J., Lee G.T., Lee K.K., Hong J.T., Ahn K.J., An I.S., An S., Bae S. (2014). Troxerutin induces protective effects against ultraviolet B radiation through the alteration of microRNA expression in human HaCaT keratinocyte cells. Int. J. Mol. Med..

[819] Cha H.J., Lee K.S., Lee G.T., Lee K.K., Hong J.T., Lee S.N., Jang H.H., Lee J.H., Park I.C., Kim Y.R. (2014). Altered miRNA expression profiles are involved in the protective effects of troxerutin against ultraviolet B radiation in normal human dermal fibroblasts. Int. J. Mol. Med..

[820] Nasri Nasrabadi P., Zareian S., Nayeri Z., Salmanipour R., Parsafar S., Gharib E., Asadzadeh Aghdaei H., Zali M.R. (2019). A detailed image of rutin underlying intracellular signaling pathways in human SW480 colorectal cancer cells based on miRNAs-lncRNAs-mRNAs-TFs interactions. J. Cell. Physiol..

[821] Krakstad C., Herfindal L., Gjertsen B.T., Bøe R., Vintermyr O.K., Fladmark K.E., Døskeland S.O. (2006). CaM-kinaseII-dependent commitment to microcystin-induced apoptosis is coupled to cell budding, but not to shrinkage or chromatin hypercondensation. Cell Death Differ..

[822] Kanno S.-I., Shouji A., Asou K., Ishikawa M. (2003). Effects of naringin on hydrogen peroxide-induced cytotoxicity and apoptosis in P388 cells. J. Pharmacol. Sci..

[823] Li L.-H., Wu L.-J., Tashiro S.-I., Onodera S., Uchiumi F., Ikejima T. (2007). Activation of the SIRT1 pathway and modulation of the cell cycle were involved in silymarin’s protection against UV-induced A375-S2 cell apoptosis. J. Asian Nat. Prod. Res..

[824] Dutta B., Park J.E., Qing I.T.Y., Kon O.L., Sze S.K. (2018). Soy-Derived Phytochemical Genistein Modifies Chromatome Topology to Restrict Cancer Cell Proliferation. Proteomics.

[825] Lyn-Cook L., Word B., George N., Lyn-Cook B., Hammons G. (2014). Effect of cigarette smoke condensate on gene promoter methylation in human lung cells. Tob. Induc. Dis..

[826] Karsli-Ceppioglu S., Ngollo M., Judes G., Penault-LLorca F., Bignon Y.J., Guy L., Bernard-Gallon D. (2015). The Role of Soy Phytoestrogens on Genetic and Epigenetic Mechanisms of Prostate Cancer. Enzymes.

[827] Dagdemir A., Durif J., Ngollo M., Bignon Y.-J., Bernard-Gallon D. (2013). Histone lysine trimethylation or acetylation can be modulated by phytoestrogen, estrogen or anti-HDAC in breast cancer cell lines. Epigenomics.

[828] Gao Y., Tollefsbol T.O. (2018). Combinational Proanthocyanidins and Resveratrol Synergistically Inhibit Human Breast Cancer Cells and Impact Epigenetic−Mediating Machinery. Int. J. Mol. Sci..

[829] Liu B., Zhang H., Tan X., Yang D., Lv Z., Jiang H., Lu J., Baiyun R., Zhang Z. (2017). GSPE reduces lead-induced oxidative stress by activating the Nrf2 pathway and suppressing miR153 and GSK-3β in rat kidney. Oncotarget.

[830] Rahnasto-Rilla M., Tyni J., Huovinen M., Jarho E., Kulikowicz T., Ravichandran S., A Bohr V., Ferrucci L., Lahtela-Kakkonen M., Moaddel R. (2018). Natural polyphenols as sirtuin 6 modulators. Sci. Rep..

[831] Li X., Yao Z., Yang D., Jiang X., Sun J., Tian L., Hu J., Wu B., Bai W. (2020). Cyanidin-3-O-glucoside restores spermatogenic dysfunction in cadmium-exposed pubertal mice via histone ubiquitination and mitigating oxidative damage. J. Hazard. Mater..

[832] Latorre E., Birar V.C., Sheerin A.N., Jeynes J., Hooper A., Dawe H.R., Melzer D., Cox L.S., Faragher R., Ostler E.L. (2017). Small molecule modulation of splicing factor expression is associated with rescue from cellular senescence. BMC Cell Biol..

[833] Liu B., Ghosh S., Yang X., Zheng H., Liu X., Wang Z., Jin G., Zheng B., Kennedy B.K., Suh Y. (2012). Resveratrol rescues SIRT1-dependent adult stem cell decline and alleviates progeroid features in laminopathy-based progeria. Cell Metab..

[834] Giovannelli L., Pitozzi V., Jacomelli M., Mulinacci N., Laurenzana A., Dolara P., Mocali A. (2011). Protective effects of resveratrol against senescence-associated changes in cultured human fibroblasts. J. Gerontol. Ser. A Biol. Sci. Med. Sci..

[835] Zhang L., Tu R., Wang Y., Hu Y., Li X., Cheng X., Yin Y., Li W., Huang H. (2017). Early-Life Exposure to Lead Induces Cognitive Impairment in Elder Mice Targeting SIRT1 Phosphorylation and Oxidative Alterations. Front. Physiol..

[836] Ghosh S., Liu B., Zhou Z. (2013). Resveratrol activates SIRT1 in a Lamin A-dependent manner. Cell Cycle.

[837] Keuser B., Khobta A., Gallé K., Anderhub S., Schulz I., Pauly K., Epe B. (2013). Influences of histone deacetylase inhibitors and resveratrol on DNA repair and chromatin compaction. Mutagenesis.

[838] Maugeri A., Barchitta M., Mazzone M.G., Giuliano F., Basile G., Agodi A. (2018). Resveratrol Modulates SIRT1 and DNMT Functions and Restores LINE-1 Methylation Levels in ARPE-19 Cells under Oxidative Stress and Inflammation. Int. J. Mol. Sci..

[839] Chen A.C.H., Peng Q., Fong S.W., Yeung W.S.B., Lee Y.L. (2020). Sirt1 is regulated by miR-135a and involved in DNA damage repair during mouse cellular reprogramming. Aging.

[840] Liu J., Chen S., Biswas S., Nagrani N., Chu Y., Chakrabarti S., Feng B. (2020). Glucose-induced oxidative stress and accelerated aging in endothelial cells are mediated by the depletion of mitochondrial SIRTs. Physiol. Rep..

[841] Mohammed E.T., Hashem K.S., Abdelazem A.Z., Foda F. (2020). Prospective Protective Effect of Ellagic Acid as a SIRT1 Activator in Iron Oxide Nanoparticle-Induced Renal Damage in Rats. Boil. Trace Elem. Res..

[842] Shanmugam P.S.T., Nair R.P., DeBenedetti A., Caldito G., Abreo F., Sunavala-Dossabhoy G. (2016). DNA damage response and repair data with pharmacological modulators of Tousled. Data Brief.

[843] Shanmugam P.S.T., Nair R.P., DeBenedetti A., Caldito G., Abreo F., Sunavala-Dossabhoy G. (2016). Tousled kinase activator, gallic acid, promotes homologous recombinational repair and suppresses radiation cytotoxicity in salivary gland cells. Free. Radic. Biol. Med..

[844] Wang C., Shu L., Zhang C., Li W., Wu R., Guo Y., Yang Y., Kong A.N. (2018). Histone Methyltransferase Setd7 Regulates Nrf2 Signaling Pathway by Phenethyl Isothiocyanate and Ursolic Acid in Human Prostate Cancer Cells. Mol. Nutr. Food Res..

[845] Chan L.Y., Kwok H.H., Chan R.W., Peiris M.J., Mak N.K., Wong R.N., Chan M.C., Yue P.Y. (2011). Dual functions of ginsenosides in protecting human endothelial cells against influenza H9N2-induced inflammation and apoptosis. J. Ethnopharmacol..

[846] Fan C., Ma Q., Xu M., Qiao Y., Zhang Y., Li P., Bi Y., Tang M. (2019). Ginsenoside Rb1 Attenuates High Glucose-Induced Oxidative Injury via the NAD-PARP-SIRT Axis in Rat Retinal Capillary Endothelial Cells. Int. J. Mol. Sci..

[847] Valdecantos M.P., Pérez-Matute P., González-Muniesa P., Prieto-Hontoria P.L., Moreno-Aliaga M.J., Martínez J.A. (2012). Lipoic acid improves mitochondrial function in nonalcoholic steatosis through the stimulation of sirtuin 1 and sirtuin 3. Obesity.

[848] Scheibye-Knudsen M., Mitchell S.J., Fang E.F., Iyama T., Ward T., Wang J., Dunn C.A., Singh N., Veith S., Hasan-Olive M.M. (2014). A high-fat diet and NAD(+) activate Sirt1 to rescue premature aging in cockayne syndrome. Cell Metab..

[849] Park J.S., Kim Y.J. (2020). Anti-Aging Effect of the Ketone Metabolite beta-Hydroxybutyrate in Drosophila Intestinal Stem Cells. Int. J. Mol. Sci..

[850] Gabriel D., Roedl D., Gordon L.B., Djabali K. (2015). Sulforaphane enhances progerin clearance in Hutchinson-Gilford progeria fibroblasts. Aging Cell.

[851] Katoch O., Kumar A., Adhikari J.S., Dwarakanath B.S., Agrawala P.K. (2013). Sulforaphane mitigates genotoxicity induced by radiation and anticancer drugs in human lymphocytes. Mutat. Res..

[852] Gabriel D., Shafry D.D., Gordon L.B., Djabali K. (2017). Intermittent treatment with farnesyltransferase inhibitor and sulforaphane improves cellular homeostasis in Hutchinson-Gilford progeria fibroblasts. Oncotarget.

[853] Dos Santos P., Machado A., De Grandis R.A., Ribeiro D.L., Tuttis K., Morselli M., Aissa A.F., Pellegrini M., Antunes L. (2020). Transcriptome and DNA methylation changes modulated by sulforaphane induce cell cycle arrest, apoptosis, DNA damage, and suppression of proliferation in human liver cancer cells. Food Chem. Toxicol..

[854] Li S., Yang Y., Sargsyan D., Wu R., Yin R., Kuo H.D., Yang I., Wang L., Cheng D., Ramirez C.N. (2020). Epigenome, Transcriptome, and Protection by Sulforaphane at Different Stages of UVB-Induced Skin Carcinogenesis. Cancer Prev. Res..

[855] Ao Y., Zhang J., Liu Z., Qian M., Li Y., Wu Z., Sun P., Wu J., Bei W., Wen J. (2019). Lamin A buffers CK2 kinase activity to modulate aging in a progeria mouse model. Sci. Adv..

[856] Minguzzi M., Guidotti S., Platano D., D’Adamo S., Cetrullo S., Assirelli E., Santi S., Mariani E., Trisolino G., Filardo G. (2019). Polyamine supplementation reduces DNA damage in adipose stem cells cultured in 3-D. Sci. Rep..

[857] Nayvelt I., Hyvönen M.T., Alhonen L., Pandya I., Thomas T., Khomutov A.R., Vepsäläinen J., Patel R., Keinänen T.A., Thomas T.J. (2010). DNA condensation by chiral alpha-methylated polyamine analogues and protection of cellular DNA from oxidative damage. Biomacromolecules.

[858] Tamura H., Kawamoto M., Sato S., Tamura I., Maekawa R., Taketani T., Aasada H., Takaki E., Nakai A., Reiter R.J. (2017). Long-term melatonin treatment delays ovarian aging. J. Pineal Res..

[859] Sarabia L., Maurer I., Bustos-Obregón E. (2009). Melatonin prevents damage elicited by the organophosphorous pesticide diazinon on mouse sperm DNA. Ecotoxicol. Environ. Saf..

[860] Fang Y., Zhang J., Li Y., Guo X., Li J., Zhong R., Zhang X. (2019). Melatonin-induced demethylation of antioxidant genes increases antioxidant capacity through RORα in cumulus cells of prepubertal lambs. Free. Radic. Biol. Med..

[861] Cai B., Ma W., Bi C., Yang F., Zhang L., Han Z., Huang Q., Ding F., Li Y., Yan G. (2016). Long noncoding RNA H19 mediates melatonin inhibition of premature senescence of c-kit(+) cardiac progenitor cells by promoting miR-675. J. Pineal Res..

[862] Mori F., Ferraiuolo M., Santoro R., Sacconi A., Goeman F., Pallocca M., Pulito C., Korita E., Fanciulli M., Muti P. (2016). Multitargeting activity of miR-24 inhibits long-term melatonin anticancer effects. Oncotarget.

[863] Gonçalves B.F., de Campos S.G.P., Góes R.M., Scarano W.R., Taboga S.R., Vilamaior P.S.L. (2017). Dual action of high estradiol doses on MNU-induced prostate neoplasms in a rodent model with high serum testosterone: Protective effect and emergence of unstable epithelial microenvironment. Prostate.

[864] Scuto A., Kirschbaum M., Buettner R., Kujawski M., Cermak J.M., Atadja P., Jove R. (2013). SIRT1 activation enhances HDAC inhibition-mediated upregulation of GADD45G by repressing the binding of NF-κB/STAT3 complex to its promoter in malignant lymphoid cells. Cell Death Dis..

[865] Meschini R., Morucci E., Berni A., Lopez-Martinez W., Palitti F. (2015). Role of chromatin structure modulation by the histone deacetylase inhibitor trichostatin A on the radio-sensitivity of ataxia telangiectasia. Mutat. Res..

[866] Egidi A., Filippi S., Manganello F., Lopez-Martinez W., Meschini R. (2018). Modulation of chromatin conformation by the histone deacetylase inhibitor trichostatin A promotes the removal of radiation-induced lesions in ataxia telangiectasia cell lines. Mutat. Res. Genet. Toxicol. Environ. Mutat. Res. Toxicol. Environ. Mutagen..

[867] Ceruti J.M., Ogara M.F., Menéndez C., Palmero I., Cánepa E.T. (2013). Inhibitor of growth 1 (ING1) acts at early steps of multiple DNA repair pathways. Mol. Cell. Biochem..

[868] Zhang A.L., Chen L., Ma L., Ding X.J., Tang S.F., Zhang A.H., Li J. (2020). Role of H3K18ac-regulated nucleotide excision repair-related genes in arsenic-induced DNA damage and repair of HaCaT cells. Hum. Exp. Toxicol..

[869] Pugh J.L., Sukhina A.S., Seed T.M., Manley N.R., Sempowski G.D., van den Brink M.R., Smithey M.J., Nikolich-Žugich J. (2014). Histone deacetylation critically determines T cell subset radiosensitivity. J. Immunol..

[870] Souliotis V.L., Vougas K., Gorgoulis V.G., Sfikakis P.P. (2016). Defective DNA repair and chromatin organization in patients with quiescent systemic lupus erythematosus. Arthritis Res. Ther..

[871] Haldar S., Dru C., Mishra R., Tripathi M., Duong F., Angara B., Fernandez A., Arditi M., Bhowmick N.A. (2016). Histone deacetylase inhibitors mediate DNA damage repair in ameliorating hemorrhagic cystitis. Sci. Rep..

[872] Tian X.L., Lu X., Feng J.B., Cai T.J., Li S., Tian M., Liu Q.J. (2018). Alterations in histone acetylation following exposure to Co γ-rays and their relationship with chromosome damage in human lymphoblastoid cells. Radiat. Environ. Biophys..

[873] Marchion D.C., Bicaku E., Daud A.I., Sullivan D.M., Munster P.N. (2005). Valproic acid alters chromatin structure by regulation of chromatin modulation proteins. Cancer Res..

[874] Sha K., Winn L.M. (2010). Characterization of valproic acid-initiated homologous recombination. Birth Defects Res. Part B Dev. Reprod. Toxicol..

[875] Oh Y.S., Kim S.H., Cho G.-W. (2016). Functional Restoration of Amyotrophic Lateral Sclerosis Patient-Derived Mesenchymal Stromal Cells through Inhibition of DNA Methyltransferase. Cell. Mol. Neurobiol..

[876] Han X., Tai H., Wang X., Wang Z., Zhou J., Wei X., Ding Y., Gong H., Mo C., Zhang J. (2016). AMPK activation protects cells from oxidative stress-induced senescence via autophagic flux restoration and intracellular NAD(+) elevation. Aging Cell.

[877] Karnewar S., Neeli P.K., Panuganti D., Kotagiri S., Mallappa S., Jain N., Jerald M.K., Kotamraju S. (2018). Metformin regulates mitochondrial biogenesis and senescence through AMPK mediated H3K79 methylation: Relevance in age-associated vascular dysfunction. Biochim. Biophys. Acta (BBA) Mol. Basis Dis..

[878] Finley J. (2018). Cellular stress and AMPK activation as a common mechanism of action linking the effects of metformin and diverse compounds that alleviate accelerated aging defects in Hutchinson-Gilford progeria syndrome. Med. Hypotheses.

[879] Egesipe A.L., Blondel S., Lo Cicero A., Jaskowiak A.L., Navarro C., Sandre-Giovannoli A., Levy N., Peschanski M., Nissan X. (2016). Metformin decreases progerin expression and alleviates pathological defects of Hutchinson-Gilford progeria syndrome cells. NPJ Aging Mech. Dis..

[880] Izzotti A., Balansky R., D’Agostini F., Longobardi M., Cartiglia C., Micale R.T., La Maestra S., Camoirano A., Ganchev G., Iltcheva M. (2014). Modulation by metformin of molecular and histopathological alterations in the lung of cigarette smoke-exposed mice. Cancer Med..

[881] Cenni V., Capanni C., Mattioli E., Columbaro M., Wehnert M., Ortolani M., Fini M., Novelli G., Bertacchini J., Maraldi N.M. (2014). Rapamycin treatment of Mandibuloacral dysplasia cells rescues localization of chromatin-associated proteins and cell cycle dynamics. Aging.

[882] Horvath S., Lu A.T., Cohen H., Raj K. (2019). Rapamycin retards epigenetic ageing of keratinocytes independently of its effects on replicative senescence, proliferation and differentiation. Aging.

[883] Nwanaji-Enwerem J.C., Colicino E., Specht A.J., Gao X., Wang C., Vokonas P., Weisskopf M.G., Boyer E.W., Baccarelli A.A., Schwartz J. (2020). Individual species and cumulative mixture relationships of 24-hour urine metal concentrations with DNA methylation age variables in older men. Environ. Res..

[884] Gadecka A., Bielak-Zmijewska A. (2019). Slowing Down Ageing: The Role of Nutrients and Microbiota in Modulation of the Epigenome. Nutrients.

[885] Caterina R.D.E., Martinez J.A., Kohlmeier M. (2020). Principles of Nutrigenetics and Nutrigenomics.

[886] Morgan A.E., Davies T.J., Auley M.T.M. (2018). The role of DNA methylation in ageing and cancer. Proc. Nutr. Soc..

[887] Xie W., Baylin S.B., Easwaran H. (2019). DNA methylation in senescence, aging and cancer. Oncoscience.

[888] Michalak E.M., Burr M.L., Bannister A.J., Dawson M.A. (2019). The roles of DNA, RNA and histone methylation in ageing and cancer. Nat. Rev. Mol. Cell Boil..

[889] Gudas L.J. (2013). Retinoids induce stem cell differentiation via epigenetic changes. Semin. Cell Dev. Biol..

[890] Nur S.M., Rath S., Ahmad V., Ahmad A., Ateeq B., Khan M.I. (2020). Nutritive vitamins as epidrugs. Crit. Rev. Food Sci. Nutr..

[891] Soda K. (2019). Spermine and gene methylation: A mechanism of lifespan extension induced by polyamine-rich diet. Amino Acids.

[892] Bridgeman S.C., Ellison G.C., Melton P.E., Newsholme P., Mamotte C.D.S. (2018). Epigenetic effects of metformin: From molecular mechanisms to clinical implications. Diabetes. Obes. Metab..

[893] Agathocleous M., Meacham C.E., Burgess R.J., Piskounova E., Zhao Z., Crane G.M., Cowin B.L., Bruner E., Murphy M.M., Chen W. (2017). Ascorbate regulates haematopoietic stem cell function and leukaemogenesis. Nature.

[894] Mazzone R., Zwergel C., Mai A., Valente S. (2017). Epi-drugs in combination with immunotherapy: A new avenue to improve anticancer efficacy. Clin. Epigenet..

[895] Myasoedova V.A., Sukhorukov V., Grechko A.V., Zhang D., Romanenko E., Orekhov V., Orekhov A.N. (2019). Inhibitors of DNA Methylation and Histone Deacetylation as Epigenetically Active Drugs for Anticancer Therapy. Curr. Pharm. Des..

[896] Ponnusamy L., Mahalingaiah P.K.S., Chang Y.-W., Singh K.P. (2019). Role of cellular reprogramming and epigenetic dysregulation in acquired chemoresistance in breast cancer. Cancer Drug Resist..

[897] Molina-Serrano D., Kyriakou D., Kirmizis A. (2019). Histone Modifications as an Intersection between Diet and Longevity. Front. Genet..

[898] Lee S.-H., Lee J.-H., Lee H.-Y., Min K.-J. (2019). Sirtuin signaling in cellular senescence and aging. BMB Rep..

[899] Gomes A.P., Price N.L., Ling A.J., Moslehi J.J., Montgomery M.K., Rajman L., White J.P., Teodoro J.S., Wrann C.D., Hubbard B.P. (2013). Declining NAD(+) induces a pseudohypoxic state disrupting nuclear-mitochondrial communication during aging. Cell.

[900] Braidy N., Berg J., Clement J., Khorshidi F., Poljak A., Jayasena T., Grant R., Sachdev P. (2019). Role of Nicotinamide Adenine Dinucleotide and Related Precursors as Therapeutic Targets for Age-Related Degenerative Diseases: Rationale, Biochemistry, Pharmacokinetics, and Outcomes. Antioxid. Redox Signal..

[901] Connell N.J., Houtkooper R.H., Schrauwen P. (2019). NAD metabolism as a target for metabolic health: Have we found the silver bullet?. Diabetologia.

[902] Fang E.F., Lautrup S., Hou Y., Demarest T.G., Croteau D.L., Mattson M.P., Bohr V.A. (2017). NAD in Aging: Molecular Mechanisms and Translational Implications. Trends Mol. Med..

[903] Mills K.F. (2016). Long-Term Administration of Nicotinamide Mononucleotide Mitigates Age-Associated Physiological Decline in Mice. Cell Metab..

[904] de Picciotto N.E. (2016). Nicotinamide Mononucleotide Supplementation Reverses Vascular Dysfunction and Oxidative Stress With Aging in Mice. Aging Cell.

[905] Cantó C. (2012). The NAD(+) Precursor Nicotinamide Riboside Enhances Oxidative Metabolism and Protects Against High-Fat Diet-Induced Obesity. Cell Metab..

[906] Fania L., Mazzanti C., Campione E., Candi E., Abeni D., Dellambra E. (2019). Role of Nicotinamide in Genomic Stability and Skin Cancer Chemoprevention. Int. J. Mol. Sci..

[907] Dai H., Sinclair D.A., Ellis J.L., Steegborn C. (2018). Sirtuin activators and inhibitors: Promises, achievements, and challenges. Pharmacol. Ther..

[908] Bradley E.W., Carpio L.R., van Wijnen A.J., McGee-Lawrence M.E., Westendorf J.J. (2015). Histone Deacetylases in Bone Development and Skeletal Disorders. Physiol. Rev..

[909] Vitiello M., Zullo A., Servillo L., Mancini F.P., Borriello A., Giovane A., Della Ragione F., D’Onofrio N., Balestrieri M.L. (2017). Multiple pathways of SIRT6 at the crossroads in the control of longevity, cancer, and cardiovascular diseases. Ageing Res. Rev..

[910] Lerrer B., Gertler A.A., Cohen H.Y. (2016). The complex role of SIRT6 in carcinogenesis. Carcinogenesis.

[911] Peleg S., Sananbenesi F., Zovoilis A., Burkhardt S., Bahari-Javan S., Agis-Balboa R.C., Cota P., Wittnam J.L., Gogol-Doering A., Opitz L. (2010). Altered histone acetylation is associated with age-dependent memory impairment in mice. Science.

[912] Krishnan V., Chow M.Z., Wang Z., Zhang L., Liu B., Liu X., Zhou Z. (2011). Histone H4 lysine 16 hypoacetylation is associated with defective DNA repair and premature senescence in Zmpste24-deficient mice. Proc. Natl. Acad. Sci. USA.

[913] Singh P., Thakur M.K. (2018). Histone Deacetylase 2 Inhibition Attenuates Downregulation of Hippocampal Plasticity Gene Expression during Aging. Mol. Neurobiol..

[914] Narayanan B.A., Narayanan N.K., Re G.G., Nixon D.W. (2003). Differential expression of genes induced by resveratrol in LNCaP cells: P53-mediated molecular targets. Int. J. Cancer.

[915] Edwards C., Canfield J., Copes N., Rehan M., Lipps D., Bradshaw P.C. (2014). D-beta-hydroxybutyrate extends lifespan in C. elegans. Aging.

[916] Delgado-Morales R., Agís-Balboa R.C., Esteller M., Berdasco M. (2017). Epigenetic mechanisms during ageing and neurogenesis as novel therapeutic avenues in human brain disorders. Clin. Epigenet..

[917] Carotenuto F., Albertini M.C., Coletti D., Vilmercati A., Campanella L., Darzynkiewicz Z., Teodori L. (2016). How Diet Intervention via Modulation of DNA Damage Response through MicroRNAs Have an Effect on Cancer Prevention and Aging, an in Silico Study. Int. J. Mol. Sci..

[918] Majidinia M., Mir S.M., Mirza-Aghazadeh-Attari M., Asghari R., Kafil H.S., Safa A., Mahmoodpoor A., Yousefi B. (2020). MicroRNAs, DNA Damage Response and Ageing. Biogerontology.

[919] Ghasemi S. (2019). Cancer’s Epigenetic Drugs: Where Are They in the Cancer Medicines?. Pharm. J..

[920] van Rooij E., Kauppinen S. (2014). Development of microRNA therapeutics is coming of age. EMBO Mol. Med..

[921] Noren Hooten N., Martin-Montalvo A., Dluzen D.F., Zhang Y., Bernier M., Zonderman A.B., Becker K.G., Gorospe M., de Cabo R., Evans M.K. (2016). Metformin-mediated increase in DICER1 regulates microRNA expression and cellular senescence. Aging Cell.

[922] Pinto S., Sato V.N., De-Souza E.A., Ferraz R.C., Camara H., Pinca A., Mazzotti D.R., Lovci M.T., Tonon G., Lopes-Ramos C.M. (2018). Enoxacin extends lifespan of C. elegans by inhibiting miR-34-5p and promoting mitohormesis. Redox Biol..

[923] Gioia U., Francia S., Cabrini M., Brambillasca S., Michelini F., Jones-Weinert C.W., d’Adda di Fagagna F. (2019). Pharmacological boost of DNA damage response and repair by enhanced biogenesis of DNA damage response RNAs. Sci. Rep..

[924] Piekarowicz K., Machowska M., Dzianisava V., Rzepecki R. (2019). Hutchinson-Gilford Progeria Syndrome-Current Status and Prospects for Gene Therapy Treatment. Cells.

[925] Saxena S., Kumar S. (2020). Pharmacotherapy to gene editing: Potential therapeutic approaches for Hutchinson-Gilford progeria syndrome. Geroscience.

[926] Fenech M. (2017). Vitamins Associated with Brain Aging, Mild Cognitive Impairment, and Alzheimer Disease: Biomarkers, Epidemiological and Experimental Evidence, Plausible Mechanisms, and Knowledge Gaps. Adv. Nutr..

[927] Samavarchi Tehrani S., Mahmoodzadeh Hosseini H., Yousefi T., Abolghasemi M., Qujeq D., Maniati M., Amani J. (2018). The crosstalk between trace elements with DNA damage response, repair, and oxidative stress in cancer. J. Cell. Biochem..

[928] Sharif R., Thomas P., Zalewski P., Fenech M. (2012). The role of zinc in genomic stability. Mutat. Res..

[929] Sfera A., Bullock K., Price A., Inderias L., Osorio C. (2018). Ferrosenescence: The iron age of neurodegeneration?. Mech. Ageing Dev..

[930] Fenech M. (2010). Folate, DNA damage and the aging brain. Mech. Ageing Dev..

[931] Simon K.W., Ma H., Dombkowski A.A., Cabelof D.C. (2012). Aging alters folate homeostasis and DNA damage response in colon. Mech. Ageing Dev..

[932] Ventrella-Lucente L.F., Unnikrishnan A., Pilling A.B., Patel H.V., Kushwaha D., Dombkowski A.A., Schmelz E.M., Cabelof D.C., Heydari A.R. (2010). Folate deficiency provides protection against colon carcinogenesis in DNA polymerase beta haploinsufficient mice. J. Biol. Chem..

[933] Graziano S., Johnston R., Deng O., Zhang J., Gonzalo S. (2016). Vitamin D/vitamin D receptor axis regulates DNA repair during oncogene-induced senescence. Oncogene.

[934] Drapkina O.M., Shepel R.N., Fomin V.V., Svistu A.A. (2018). Place of vitamin D in the prevention of premature aging and the development of age-associated diseases. Ter. Arkhiv.

[935] Gruz-Gibelli E., Chessel N., Allioux C., Marin P., Piotton F., Leuba G., Herrmann F.R., Savioz A. (2016). The Vitamin A Derivative All-Trans Retinoic Acid Repairs Amyloid-β-Induced Double-Strand Breaks in Neural Cells and in the Murine Neocortex. Neural Plast..

[936] Zuchegna C., Aceto F., Bertoni A., Romano A., Perillo B., Laccetti P., Gottesman M.E., Avvedimento E.V., Porcellini A. (2014). Mechanism of retinoic acid-induced transcription: Histone code, DNA oxidation and formation of chromatin loops. Nucleic Acids Res..

[937] Demetriou S.K., Ona-Vu K., Teichert A.E., Cleaver J.E., Bikle D.D., Oh D.H. (2012). Vitamin D receptor mediates DNA repair and is UV inducible in intact epidermis but not in cultured keratinocytes. J. Investig. Dermatol..

[938] Setayesh T., Nersesyan A., Mišík M., Ferk F., Langie S., Andrade V.M., Haslberger A., Knasmüller S. (2018). Impact of obesity and overweight on DNA stability: Few facts and many hypotheses. Mutat. Res..

[939] Dziaman T., Huzarski T., Gackowski D., Rozalski R., Siomek A., Szpila A., Guz J., Lubinski J., Wasowicz W., Roszkowski K. (2009). Selenium supplementation reduced oxidative DNA damage in adnexectomized BRCA1 mutations carriers. Cancer Epidemiol. Biomark. Prev. Publ. Am. Assoc. Cancer Res. Cosponsored Am. Soc. Prev. Oncol..

[940] Qi Y., Schoene N.W., Lartey F.M., Cheng W.-H. (2010). Selenium compounds activate ATM-dependent DNA damage response via the mismatch repair protein hMLH1 in colorectal cancer cells. J. Biol. Chem..

[941] Fischer J.L., Lancia J.K., Mathur A., Smith M.L. (2006). Selenium protection from DNA damage involves a Ref1/p53/Brca1 protein complex. Anticancer Res..

[942] Sarveswaran S., Liroff J., Zhou Z., Nikitin A.Y., Ghosh J. (2010). Selenite triggers rapid transcriptional activation of p53, and p53-mediated apoptosis in prostate cancer cells: Implication for the treatment of early-stage prostate cancer. Int. J. Oncol..

[943] Favrot C., Beal D., Blouin E., Leccia M.T., Roussel A.M., Rachidi W. (2018). Age-Dependent Protective Effect of Selenium against UVA Irradiation in Primary Human Keratinocytes and the Associated DNA Repair Signature. Oxidative Med. Cell. Longev..

[944] Song Y., Leonard S.W., Traber M.G., Ho E. (2009). Zinc deficiency affects DNA damage, oxidative stress, antioxidant defenses, and DNA repair in rats. J. Nutr..

[945] Cooper K.L., King B.S., Sandoval M.M., Liu K.J., Hudson L.G. (2013). Reduction of arsenite-enhanced ultraviolet radiation-induced DNA damage by supplemental zinc. Toxicol. Appl. Pharmacol..

[946] Ho E., Ames B.N. (2002). Low intracellular zinc induces oxidative DNA damage, disrupts p53, NFkappa B, and AP1 DNA binding, and affects DNA repair in a rat glioma cell line. Proc. Natl. Acad. Sci. USA.

[947] Sun X., Zhou X., Du L., Liu W., Liu Y., Hudson L.G., Liu K.J. (2014). Arsenite binding-induced zinc loss from PARP-1 is equivalent to zinc deficiency in reducing PARP-1 activity, leading to inhibition of DNA repair. Toxicol. Appl. Pharmacol..

[948] Ding X., Zhou X., Cooper K.L., Huestis J., Hudson L.G., Liu K.J. (2017). Differential sensitivities of cellular XPA and PARP-1 to arsenite inhibition and zinc rescue. Toxicol. Appl. Pharmacol..

[949] Deepa A., Naveena K., Anindya R. (2018). DNA repair activity of Fe(II)/2OG-dependent dioxygenases affected by low iron level in Saccharomyces cerevisiae. FEMS Yeast Res..

[950] Musallam K.M., Taher A.T. (2018). Iron deficiency beyond erythropoiesis: Should we be concerned?. Curr. Med. Res. Opin..

[951] Saletta F., Rahmanto Y.S., Siafakas A.R., Richardson D.R. (2011). Cellular iron depletion and the mechanisms involved in the iron-dependent regulation of the growth arrest and DNA damage family of genes. J. Biol. Chem..

[952] Foley M.C., Couto L., Rauf S., Boyke A. (2019). Insights into DNA polymerase δ’s mechanism for accurate DNA replication. J. Mol. Model..

[953] Exell J.C., Thompson M.J., Finger L.D., Shaw S.J., Debreczeni J., Ward T.A., McWhirter C., Siöberg C.L., Martinez Molina D., Abbott W.M. (2016). Cellularly active N-hydroxyurea FEN1 inhibitors block substrate entry to the active site. Nat. Chem. Biol..

[954] Jamsen J.A., Beard W.A., Pedersen L.C., Shock D.D., Moon A.F., Krahn J.M., Bebenek K., Kunkel T.A., Wilson S.H. (2017). Time-lapse crystallography snapshots of a double-strand break repair polymerase in action. Nat. Commun..

[955] Ruas F.A.D., Barboza N.R., Castro-Borges W., Guerra-Sá R. (2019). Manganese alters expression of proteins involved in the oxidative stress of Meyerozyma guilliermondii. J. Proteom..

[956] Hutfilz C.R., Wang N.E., Hoff C.A., Lee J.A., Hackert B.J., Courcelle J., Courcelle C.T. (2019). Manganese Is Required for the Rapid Recovery of DNA Synthesis following Oxidative Challenge in *Escherichia coli*. J. Bacteriol..

[957] Park S.-H., Lim J.S., Jang K.L. (2011). All-trans retinoic acid induces cellular senescence via upregulation of p16, p21, and p27. Cancer Lett..

[958] di Masi A., Cilli D., Berardinelli F., Talarico A., Pallavicini I., Pennisi R., Leone S., Antoccia A., Noguera N.I., Lo-Coco F. (2016). PML nuclear body disruption impairs DNA double-strand break sensing and repair in APL. Cell Death Dis..

[959] Weidele K., Beneke S., Bürkle A. (2017). The NAD precursor nicotinic acid improves genomic integrity in human peripheral blood mononuclear cells after X-irradiation. DNA Repair.

[960] Benavente C.A., Schnell S.A., Jacobson E.L. (2012). Effects of niacin restriction on sirtuin and PARP responses to photodamage in human skin. PLoS ONE.

[961] Batra V., Kislay B. (2013). Mitigation of gamma-radiation induced abasic sites in genomic DNA by dietary nicotinamide supplementation: Metabolic up-regulation of NAD(+) biosynthesis. Mutat. Res..

[962] Peters K.M., Borradaile N.M. (2019). Microarray data and pathway analyses for human microvascular endothelial cells supplemented with low dose vitamin D or niacin during lipotoxicity. Data Brief.

[963] Malesu R., Martin A.J., Lyons J.G., Scolyer R.A., Chen A.C., McKenzie C.A., Madore J., Halliday G.M., Damian D.L. (2020). Nicotinamide for skin cancer chemoprevention: Effects of nicotinamide on melanoma in vitro and in vivo. Photochem. Photobiol. Sci..

[964] Wilk A., Hayat F., Cunningham R., Li J., Garavaglia S., Zamani L., Ferraris D.M., Sykora P., Andrews J., Clark J. (2020). Extracellular NAD(+) enhances PARP-dependent DNA repair capacity independently of CD73 activity. Sci. Rep..

[965] Shimoi K., Akaiwa E., Mori N., Sano M., Nakamura Y., Tomita I. (1992). Bio-antimutagenic activities of vitamin B6 in E. coli and mouse peripheral blood cells. Mutat. Res..

[966] Wang P., Chen Y., Wang L., Wu Y., Wang L., Wu Y., Gong Z. (2019). The intervention mechanism of folic acid for benzo(a)pyrene toxic effects in vitro and in vivo. Eur. J. Cancer Prev. Off. J. Eur. Cancer Prev. Organ..

[967] Sanchez H., Hossain M.B., Lera L., Hirsch S., Albala C., Uauy R., Broberg K., Ronco A.M. (2017). High levels of circulating folate concentrations are associated with DNA methylation of tumor suppressor and repair genes, and in elderly Chileans. Clin. Epigenet..

[968] Price R.J., Lillycrop K.A., Burdge G.C. (2015). Folic acid supplementation in vitro induces cell type-specific changes in BRCA1 and BRCA 2 mRNA expression, but does not alter DNA methylation of their promoters or DNA repair. Nutr. Res..

[969] Li H., Wang X., Zhao H., Wang F., Bao Y., Guo J., Chang S., Wu L., Cheng H., Chen S. (2019). Low folate concentration impacts mismatch repair deficiency in neural tube defects. Epigenomics.

[970] Rubis B., Luczak M.W., Krawic C., Zhitkovich A. (2019). Vitamin C increases DNA breaks and suppresses DNA damage-independent activation of ATM by bleomycin. Free Radic. Biol. Med..

[971] Rybchyn M.S., De Silva W., Sequeira V.B., McCarthy B.Y., Dilley A.V., Dixon K.M., Halliday G.M., Mason R.S. (2018). Enhanced Repair of UV-Induced DNA Damage by 1,25-Dihydroxyvitamin D in Skin Is Linked to Pathways that Control Cellular Energy. J. Investig. Dermatol..

[972] Zhu H., Guo D., Li K., Pedersen-White J., Stallmann-Jorgensen I.S., Huang Y., Parikh S., Liu K., Dong Y. (2012). Increased telomerase activity and vitamin D supplementation in overweight African Americans. Int. J. Obes..

[973] Gonzalo S. (2013). Novel roles of 1α,25(OH)2D3 on DNA repair provide new strategies for breast cancer treatment. J. Steroid Biochem. Mol. Biol..

[974] Gonzalez-Suarez I., Redwood A.B., Grotsky D.A., Neumann M.A., Cheng E.H., Stewart C.L., Dusso A., Gonzalo S. (2011). A new pathway that regulates 53BP1 stability implicates cathepsin L and vitamin D in DNA repair. EMBO J..

[975] Ali M., Shahin S.M., Sabri N.A., Al-Hendy A., Yang Q. (2019). Hypovitaminosis D exacerbates the DNA damage load in human uterine fibroids, which is ameliorated by vitamin D3 treatment. Acta Pharmacol. Sin..

[976] Coll-Bonfill N., de Faria R.C., Bhoopatiraju S., Gonzalo S. (2020). Calcitriol Prevents RAD51 Loss and cGAS-STING-IFN Response Triggered by Progerin. Proteomics.

[977] Omar S.S., Aly R.G., Badae N.M. (2017). Vitamin E improves testicular damage in streptozocin-induced diabetic rats, via increasing vascular endothelial growth factor and poly(ADP-ribose) polymerase-1. Andrologia.

[978] Pathak R., Bachri A., Ghosh S.P., Koturbash I., Boerma M., Binz R.K., Sawyer J.R., Hauer-Jensen M. (2016). The Vitamin E Analog Gamma-Tocotrienol (GT3) Suppresses Radiation-Induced Cytogenetic Damage. Pharm. Res..

[979] Lin H.-C., Song T.-Y., Hu M.-L. (2011). S-Adenosylhomocysteine enhances DNA damage through increased β-amyloid formation and inhibition of the DNA-repair enzyme OGG1b in microglial BV-2 cells. Toxicology.

[980] Aissa A.F., Gomes T.D., Almeida M.R., Hernandes L.C., Darin J.D., Bianchi M.L., Antunes L.M. (2013). Methionine concentration in the diet has a tissue-specific effect on chromosomal stability in female mice. Food Chem. Toxicol. Int. J. Publ. Br. Ind. Biol. Res. Assoc..

[981] Pogribny I.P., Shpyleva S.I., Muskhelishvili L., Bagnyukova T.V., James S.J., Beland F.A. (2009). Role of DNA damage and alterations in cytosine DNA methylation in rat liver carcinogenesis induced by a methyl-deficient diet. Mutat. Res..

[982] Düzenli U., Altun Z., Olgun Y., Aktaş S., Pamukoğlu A., Çetinayak H.O., Bayrak A.F., Olgun L. (2019). Role of N-acetyl cysteine and acetyl-l-carnitine combination treatment on DNA-damage-related genes induced by radiation in HEI-OC1 cells. Int. J. Radiat. Biol..

[983] Niture S.K., Velu C.S., Smith Q.R., Bhat G.J., Srivenugopal K.S. (2007). Increased expression of the MGMT repair protein mediated by cysteine prodrugs and chemopreventative natural products in human lymphocytes and tumor cell lines. Carcinogenesis.

[984] Wang B., Fu J., Yu T., Xu A., Qin W., Yang Z., Chen Y., Wang H. (2018). Contradictory effects of mitochondria-and non-mitochondria-targeted antioxidants on hepatocarcinogenesis by altering DNA repair in mice. Hepatology.

[985] Wang G., Ma H., Wang J., Khan M.F. (2019). Contribution of poly(ADP-ribose)polymerase-1 activation and apoptosis in trichloroethene-mediated autoimmunity. Toxicol. Appl. Pharmacol..

[986] Hou D., Liu Z., Xu X., Liu Q., Zhang X., Kong B., Wei J.J., Gong Y., Shao C. (2018). Increased oxidative stress mediates the antitumor effect of PARP inhibition in ovarian cancer. Redox Biol..

[987] Katiyar S.K., Vaid M., van Steeg H., Meeran S.M. (2010). Green tea polyphenols prevent UV-induced immunosuppression by rapid repair of DNA damage and enhancement of nucleotide excision repair genes. Cancer Prev. Res..

[988] Meeran S.M., Akhtar S., Katiyar S.K. (2009). Inhibition of UVB-induced skin tumor development by drinking green tea polyphenols is mediated through DNA repair and subsequent inhibition of inflammation. J. Investig. Dermatol..

[989] Wang Z., Xue R., Lei X., Lv J., Wu G., Li W., Xue L., Lei X., Zhao H., Gao H. (2012). Tea polyphenols increase X-ray repair cross-complementing protein 1 and apurinic/apyrimidinic endonuclease/redox factor-1 expression in the hippocampus of rats during cerebral ischemia/reperfusion injury. Neural Regen. Res..

[990] Chong S.Y., Chiang H.-Y., Chen T.-H., Liang Y.-J., Lo Y.-C. (2019). Green tea extract promotes DNA repair in a yeast model. Sci. Rep..

[991] Sinha D., Roy M. (2011). Antagonistic role of tea against sodium arsenite-induced oxidative DNA damage and inhibition of DNA repair in Swiss albino mice. J. Environ. Pathol. Toxicol. Oncol. Off. Organ Int. Soc. Environ. Toxicol. Cancer.

[992] Bartholome A., Kampkötter A., Tanner S., Sies H., Klotz L.-O. (2010). Epigallocatechin gallate-induced modulation of FoxO signaling in mammalian cells and C. elegans: FoxO stimulation is masked via PI3K/Akt activation by hydrogen peroxide formed in cell culture. Arch. Biochem. Biophys..

[993] Ni J., Guo X., Wang H., Zhou T., Wang X. (2018). Differences in the Effects of EGCG on Chromosomal Stability and Cell Growth between Normal and Colon Cancer Cells. Molecules.

[994] Srivastava A.K., Bhatnagar P., Singh M., Mishra S., Kumar P., Shukla Y., Gupta K.C. (2013). Synthesis of PLGA nanoparticles of tea polyphenols and their strong in vivo protective effect against chemically induced DNA damage. Int. J. Nanomed..

[995] Meeran S.M., Mantena S.K., Katiyar S.K. (2006). Prevention of ultraviolet radiation-induced immunosuppression by (−)-epigallocatechin-3-gallate in mice is mediated through interleukin 12-dependent DNA repair. Clin. Cancer Res..

[996] Hasegawa T., Shimada S., Ishida H., Nakashima M. (2013). Chafuroside B, an Oolong tea polyphenol, ameliorates UVB-induced DNA damage and generation of photo-immunosuppression related mediators in human keratinocytes. PLoS ONE.

[997] Britto S.M., Shanthakumari D., Agilan B., Radhiga T., Kanimozhi G., Prasad N.R. (2017). Apigenin prevents ultraviolet-B radiation induced cyclobutane pyrimidine dimers formation in human dermal fibroblasts. Mutat. Res..

[998] Das S., Das J., Paul A., Samadder A., Khuda-Bukhsh A.R. (2013). Apigenin, a bioactive flavonoid from Lycopodium clavatum, stimulates nucleotide excision repair genes to protect skin keratinocytes from ultraviolet B-induced reactive oxygen species and DNA damage. J. Acupunct. Meridian Stud..

[999] Ramos A.A., Pereira-Wilson C., Collins A.R. (2010). Protective effects of ursolic acid and luteolin against oxidative DNA damage include enhancement of DNA repair in Caco-2 cells. Mutat. Res..

[1000] Nagasaka M., Hashimoto R., Inoue Y., Ishiuchi K., Matsuno M., Itoh Y., Tokugawa M., Ohoka N., Morishita D., Mizukami H. (2018). Anti-Tumorigenic Activity of Chrysin from via Non-Genotoxic p53 Activation through the ATM-Chk2 Pathway. Molecules.

[1001] Ooko E., Kadioglu O., Greten H.J., Efferth T. (2017). Pharmacogenomic Characterization and Isobologram Analysis of the Combination of Ascorbic Acid and Curcumin-Two Main Metabolites of—In Cancer Cells. Front. Pharmacol..

[1002] Yang G., Qiu J., Wang D., Tao Y., Song Y., Wang H., Tang J., Wang X., Sun Y.U., Yang Z. (2018). Traditional Chinese Medicine Curcumin Sensitizes Human Colon Cancer to Radiation by Altering the Expression of DNA Repair-related Genes. Anticancer Res..

[1003] Naick H., Jin S., Baskaran R. (2016). GADD45α modulates curcumin sensitivity through c-Abl- and JNK-dependent signaling pathways in a mismatch repair-dependent manner. Mol. Cell. Biochem..

[1004] Tan X., Zhao C., Pan J., Shi Y., Liu G., Zhou B., Zheng R. (2009). In vivo non-enzymatic repair of DNA oxidative damage by polyphenols. Cell Biol. Int..

[1005] Min K., Ebeler S.E. (2009). Quercetin inhibits hydrogen peroxide-induced DNA damage and enhances DNA repair in Caco-2 cells. Food Chem. Toxicol. Int. J. Publ. Br. Ind. Biol. Res. Assoc..

[1006] Ye R., Goodarzi A.A., Kurz E.U., Saito S., Higashimoto Y., Lavin M.F., Appella E., Anderson C.W., Lees-Miller S.P. (2004). The isoflavonoids genistein and quercetin activate different stress signaling pathways as shown by analysis of site-specific phosphorylation of ATM, p53 and histone H2AX. DNA Repair.

[1007] Darband S.G., Sadighparvar S., Yousefi B., Kaviani M., Ghaderi-Pakdel F., Mihanfar A., Rahimi Y., Mobaraki K., Majidinia M. (2020). Quercetin attenuated oxidative DNA damage through NRF2 signaling pathway in rats with DMH induced colon carcinogenesis. Life Sci..

[1008] Maurya D.K., Balakrishnan S., Salvi V.P., Nair C.K.K. (2005). Protection of cellular DNA from gamma-radiation-induced damages and enhancement in DNA repair by troxerutin. Mol. Cell. Biochem..

[1009] Yashavarddhan M.H., Shukla S.K., Chaudhary P., Srivastava N.N., Joshi J., Suar M., Gupta M.L. (2017). Targeting DNA Repair through Podophyllotoxin and Rutin Formulation in Hematopoietic Radioprotection: An Study. Front. Pharmacol..

[1010] Hayder N., Bouhlel I., Skandrani I., Kadri M., Steiman R., Guiraud P., Mariotte A.M., Ghedira K., Dijoux-Franca M.G., Chekir-Ghedira L. (2008). In vitro antioxidant and antigenotoxic potentials of myricetin-3-o-galactoside and myricetin-3-o-rhamnoside from Myrtus communis: Modulation of expression of genes involved in cell defence system using cDNA microarray. Toxicol. Vitr. Int. J. Publ. Assoc. BIBRA.

[1011] Morel I., Abalea V., Cillard P., Cillard J. (2001). Repair of oxidized DNA by the flavonoid myricetin. Methods Enzymol..

[1012] Abalea V., Cillard J., Dubos M.P., Sergent O., Cillard P., Morel I. (1999). Repair of iron-induced DNA oxidation by the flavonoid myricetin in primary rat hepatocyte cultures. Free. Radic. Biol. Med..

[1013] Charles C., Nachtergael A., Ouedraogo M., Belayew A., Duez P. (2014). Effects of chemopreventive natural products on non-homologous end-joining DNA double-strand break repair. Mutat. Res. Genet. Toxicol. Environ. Mutagen..

[1014] Fu H., Katsumura Y., Lin M., Hata K., Muroya Y., Hatano Y. (2008). Fast repair activities towards dGMP hydroxyl radical adducts by silybin and its analogues. J. Radiat. Res..

[1015] Gao K., Henning S.M., Niu Y., Youssefian A.A., Seeram N.P., Xu A., Heber D. (2006). The citrus flavonoid naringenin stimulates DNA repair in prostate cancer cells. J. Nutr. Biochem..

[1016] Jagetia A., Jagetia G.C., Naringin S.J. (2007). A grapefruit flavanone, protects V79 cells against the bleomycin-induced genotoxicity and decline in survival. J. Appl. Toxicol. JAT.

[1017] Jin S., Zhou B., Luo D. (2011). Hesperidin promotes cyclobutane pyrimidine dimer repair in UVB-exposed mice epidermis. Ir. J. Med. Sci..

[1018] Guillermo-Lagae R., Deep G., Ting H., Agarwal C., Agarwal R. (2015). Silibinin enhances the repair of ultraviolet B-induced DNA damage by activating p53-dependent nucleotide excision repair mechanism in human dermal fibroblasts. Oncotarget.

[1019] Narayanapillai S., Agarwal C., Deep G., Agarwal R. (2014). Silibinin inhibits ultraviolet B radiation-induced DNA-damage and apoptosis by enhancing interleukin-12 expression in JB6 cells and SKH-1 hairless mouse skin. Mol. Carcinog..

[1020] Dhanalakshmi S., Agarwal C., Singh R.P., Agarwal R. (2005). Silibinin up-regulates DNA-protein kinase-dependent p53 activation to enhance UVB-induced apoptosis in mouse epithelial JB6 cells. J. Biol. Chem..

[1021] Roy S., Deep G., Agarwal C., Agarwal R. (2012). Silibinin prevents ultraviolet B radiation-induced epidermal damages in JB6 cells and mouse skin in a p53-GADD45α-dependent manner. Carcinogenesis.

[1022] Katiyar S.K., Mantena S.K., Meeran S.M. (2011). Silymarin protects epidermal keratinocytes from ultraviolet radiation-induced apoptosis and DNA damage by nucleotide excision repair mechanism. PLoS ONE.

[1023] Rigby C.M., Roy S., Deep G., Guillermo-Lagae R., Jain A.K., Dhar D., Orlicky D.J., Agarwal C., Agarwal R. (2017). Role of p53 in silibinin-mediated inhibition of ultraviolet B radiation-induced DNA damage, inflammation and skin carcinogenesis. Carcinogenesis.

[1024] Moore J.O., Wang Y., Stebbins W.G., Gao D., Zhou X., Phelps R., Lebwohl M., Wei H. (2006). Photoprotective effect of isoflavone genistein on ultraviolet B-induced pyrimidine dimer formation and PCNA expression in human reconstituted skin and its implications in dermatology and prevention of cutaneous carcinogenesis. Carcinogenesis.

[1025] Iovine B., Garofalo M., Orefice M., Giannini V., Gasparri F., Monfrecola G., Bevilacqua M.A. (2014). Isoflavones in aglycone solution enhance ultraviolet B-induced DNA damage repair efficiency. Clin. Exp. Dermatol..

[1026] Iovine B., Iannella M.L., Gasparri F., Monfrecola G., Bevilacqua M.A. (2011). Synergic Effect of Genistein and Daidzein on UVB-Induced DNA Damage: An Effective Photoprotective Combination. J. Biomed. Biotechnol..

[1027] Iovine B., Iannella M.L., Gasparri F., Giannini V., Monfrecola G., Bevilacqua A.M. (2012). A comparative analysis of the photo-protective effects of soy isoflavones in their aglycone and glucoside forms. Int. J. Mol. Sci..

[1028] Song L., Ma L., Cong F., Shen X., Jing P., Ying X., Zhou H., Jiang J., Fu Y., Yan H. (2015). Radioprotective effects of genistein on HL-7702 cells via the inhibition of apoptosis and DNA damage. Cancer Lett..

[1029] Vaid M., Prasad R., Singh T., Katiyar S.K. (2017). Dietary grape seed proanthocyanidins inactivate regulatory T cells by promoting NER-dependent DNA repair in dendritic cells in UVB-exposed skin. Oncotarget.

[1030] Vaid M., Sharma S.D., Katiyar S.K. (2010). Proanthocyanidins inhibit photocarcinogenesis through enhancement of DNA repair and xeroderma pigmentosum group A-dependent mechanism. Cancer Prev. Res..

[1031] Bakheet S.A., Alhuraishi A.M., Al-Harbi N.O., Al-Hosaini K.A., Al-Sharary S.D., Attia M.M., Alhoshani A.R., Al-Shabanah O.A., Al-Harbi M.M., Imam F. (2016). Alleviation of Aflatoxin B1-Induced Genomic Damage by Proanthocyanidins via Modulation of DNA Repair. J. Biochem. Mol. Toxicol..

[1032] Vaid M., Singh T., Prasad R., Elmets C.A., Xu H., Katiyar S.K. (2013). Bioactive grape proanthocyanidins enhance immune reactivity in UV-irradiated skin through functional activation of dendritic cells in mice. Cancer Prev. Res..

[1033] Kumar A., Choudhary S., Adhikari J.S., Chaudhury N.K. (2018). Sesamol ameliorates radiation induced DNA damage in hematopoietic system of whole body γ-irradiated mice. Environ. Mol. Mutagen..

[1034] Nair G.G., Nair C.K.K. (2010). Protection of cellular DNA and membrane from γ-radiation-induced damages and enhancement in DNA repair by sesamol. Cancer Biother. Radiopharm..

[1035] Lee J.-H., Guo Z., Myler L.R., Zheng S., Paull T.T. (2014). Direct activation of ATM by resveratrol under oxidizing conditions. PLoS ONE.

[1036] Yamamori T., DeRicco J., Naqvi A., Hoffman T.A., Mattagajasingh I., Kasuno K., Jung S.B., Kim C.S., Irani K. (2010). SIRT1 deacetylates APE1 and regulates cellular base excision repair. Nucleic Acids Res..

[1037] Jia J.-Y., Tan Z.-G., Liu M., Jiang Y.-G. (2017). Apurinic/apyrimidinic endonuclease 1 (APE1) contributes to resveratrol-induced neuroprotection against oxygen-glucose deprivation and re-oxygenation injury in HT22 cells: Involvement in reducing oxidative DNA damage. Mol. Med. Rep..

[1038] Gao P., Li N., Ji K., Wang Y., Xu C., Liu Y., Wang Q., Wang J., He N., Sun Z. (2019). Resveratrol targets TyrRS acetylation to protect against radiation-induced damage. FASEB J. Off. Publ. Fed. Am. Soc. Exp. Biol..

[1039] Uchiumi F., Shoji K., Sasaki Y., Sasaki M., Sasaki Y., Oyama T., Sugisawa K., Tanuma S. (2016). Characterization of the 5′-flanking region of the human TP53 gene and its response to the natural compound. Resveratrol. J. Biochem..

[1040] Mikuła-Pietrasik J., Kuczmarska A., Rubiś B., Filas V., Murias M., Zieliński P., Piwocka K., Książek K. (2012). Resveratrol delays replicative senescence of human mesothelial cells via mobilization of antioxidative and DNA repair mechanisms. Free. Radic. Biol. Med..

[1041] Matsuno Y., Atsumi Y., Alauddin M., Rana M.M., Fujimori H., Hyodo M., Shimizu A., Ikuta T., Tani H., Torigoe H. (2020). Resveratrol and its Related Polyphenols Contribute to the Maintenance of Genome Stability. Sci Rep..

[1042] Shiratake S., Nakahara T., Iwahashi H., Onodera T., Mizushina Y. (2015). Rose myrtle (Rhodomyrtus tomentosa) extract and its component, piceatannol, enhance the activity of DNA polymerase and suppress the inflammatory response elicited by UVB-induced DNA damage in skin cells. Mol. Med. Rep..

[1043] Ikeoka S., Nakahara T., Iwahashi H., Mizushina Y. (2016). The Establishment of an Assay to Measure DNA Polymerase-Catalyzed Repair of UVB-Induced DNA Damage in Skin Cells and Screening of DNA Polymerase Enhancers from Medicinal Plants. Int. J. Mol. Sci..

[1044] Maurya D.K., Salvi V.P., Nair C.K.K. (2005). Radiation protection of DNA by ferulic acid under in vitro and in vivo conditions. Mol. Cell. Biochem..

[1045] Silva J.P., Gomes A.C., Coutinho O.P. (2008). Oxidative DNA damage protection and repair by polyphenolic compounds in PC12 cells. Eur. J. Pharmacol..

[1046] Aiyer H.S., Vadhanam M.V., Stoyanova R., Caprio G.D., Clapper M.L., Gupta R.C. (2008). Dietary berries and ellagic acid prevent oxidative DNA damage and modulate expression of DNA repair genes. Int. J. Mol. Sci..

[1047] Abdelwahed A., Bouhlel I., Skandrani I., Valenti K., Kadri M., Guiraud P., Steiman R., Mariotte A.M., Ghedira K., Laporte F. (2007). Study of antimutagenic and antioxidant activities of gallic acid and 1,2,3,4,6-pentagalloylglucose from Pistacia lentiscus. Confirmation by microarray expression profiling. Chem. Interact..

[1048] Carneiro C.C., da Costa Santos S., de Souza Lino R., Bara M.T., Chaibub B.A., de Melo Reis P.R., Chaves D.A., da Silva A.J., Silva L.S., de Melo E Silva D. (2016). Chemopreventive effect and angiogenic activity of punicalagin isolated from leaves of Lafoensia pacari A. St.-Hil. Toxicol. Appl. Pharmacol..

[1049] Piao M.J., Hewage S.R., Han X., Kang K.A., Kang H.K., Lee N.H., Hyun J.W. (2015). Protective Effect of Diphlorethohydroxycarmalol against Ultraviolet B Radiation-Induced DNA Damage by Inducing the Nucleotide Excision Repair System in HaCaT Human Keratinocytes. Mar. Drugs.

[1050] Nikolić B., Mitić-Ćulafić D., Vuković-Gačić B., Knežević-Vukčević J. (2011). Modulation of genotoxicity and DNA repair by plant monoterpenes camphor, eucalyptol and thujone in *Escherichia coli* and mammalian cells. Food Chem. Toxicol. Int. J. Publ. Br. Ind. Biol. Res. Assoc..

[1051] Nikolić B., Vasilijević B., Mitić-Ćulafić D., Vuković-Gačić B., Knežević-Vukćević J. (2015). Comparative study of genotoxic, antigenotoxic and cytotoxic activities of monoterpenes camphor, eucalyptol and thujone in bacteria and mammalian cells. Chem. Interact..

[1052] Ramos A.A., Lima C.F., Pereira M.L., Fernandes-Ferreira M., Pereira-Wilson C. (2008). Antigenotoxic effects of quercetin, rutin and ursolic acid on HepG2 cells: Evaluation by the comet assay. Toxicol. Lett..

[1053] Chung Y.H., Jeong S.A., Choi H.S., Ro S., Lee J.S., Park J.K. (2018). Protective effects of ginsenoside Rg2 and astaxanthin mixture against UVB-induced DNA damage. Anim. Cells Syst..

[1054] Jeong S.J., Han S.H., Kim D.Y., Lee J.C., Kim H.S., Kim B.H., Lee J.S., Hwang E.H., Park J.K. (2007). Effects of mRg2, a mixture of ginsenosides containing 60% Rg2, on the ultraviolet B-induced DNA repair synthesis and apoptosis in NIH3T3 cells. Int. J. Toxicol..

[1055] Yang L.-X., Zhang X., Zhao G. (2016). Ginsenoside Rd Attenuates DNA Damage by Increasing Expression of DNA Glycosylase Endonuclease VIII-like Proteins after Focal Cerebral Ischemia. Chin. Med. J..

[1056] Cai B.-X., Jin S.-L., Luo D., Lin X.-F., Gao J. (2009). Ginsenoside Rb1 suppresses ultraviolet radiation-induced apoptosis by inducing DNA repair. Biol. Pharm. Bull..

[1057] Cai B.-X., Luo D., Lin X.-F., Gao J. (2008). Compound K suppresses ultraviolet radiation-induced apoptosis by inducing DNA repair in human keratinocytes. Arch. Pharmacal Res..

[1058] Afzal S., Garg S., Ishida Y., Terao K., Kaul S.C., Wadhwa R. (2019). Rat Glioma Cell-Based Functional Characterization of Anti-Stress and Protein Deaggregation Activities in the Marine Carotenoids, Astaxanthin and Fucoxanthin. Mar. Drugs.

[1059] Moskalev A., Shaposhnikov M., Zemskaya N., Belyi A., Dobrovolskaya E., Patova A., Guvatova Z., Lukyanova E., Snezhkina A., Kudryavtseva A. (2018). Transcriptome analysis reveals mechanisms of geroprotective effects of fucoxanthin in Drosophila. BMC Genom..

[1060] Seo J.Y., Masamune A., Shimosegawa T., Kim H. (2009). Protective effect of lycopene on oxidative stress-induced cell death of pancreatic acinar cells. Ann. N. Y. Acad. Sci..

[1061] Li D.-W., Wang Y.-D., Zhou S.-Y., Sun W.-P. (2016). α-lipoic acid exerts neuroprotective effects on neuronal cells by upregulating the expression of PCNA via the P53 pathway in neurodegenerative conditions. Mol. Med. Rep..

[1062] Hać A., Brokowska J., Rintz E., Bartkowski M., Węgrzyn G., Herman-Antosiewicz A. (2019). Mechanism of selective anticancer activity of isothiocyanates relies on differences in DNA damage repair between cancer and healthy cells. Eur. J. Nutr..

[1063] Fan S., Meng Q., Xu J., Jiao Y., Zhao L., Zhang X., Sarkar F.H., Brown M.L., Dritschilo A., Rosen E.M. (2013). DIM (3,3′-diindolylmethane) confers protection against ionizing radiation by a unique mechanism. Proc. Natl. Acad. Sci. USA.

[1064] King A.A., Shaughnessy D.T., Mure K., Leszczynska J., Ward W.O., Umbach D.M., Xu Z., Ducharme D., Taylor J.A., Demarini D.M. (2007). Antimutagenicity of cinnamaldehyde and vanillin in human cells: Global gene expression and possible role of DNA damage and repair. Mutat. Res..

[1065] Li M., Lang Y., Gu M.M., Shi J., Chen B., Yu L., Zhou P.K., Shang Z.F. (2020). Vanillin derivative VND3207 activates DNA-PKcs conferring protection against radiation-induced intestinal epithelial cells injury in vitro and in vivo. Toxicol. Appl. Pharmacol..

[1066] Li M., Gu M.M., Lang Y., Shi J., Chen B., Guan H., Yu L., Zhou P.K., Shang Z.F. (2019). The vanillin derivative VND3207 protects intestine against radiation injury by modulating p53/NOXA signaling pathway and restoring the balance of gut microbiota. Free Radic. Biol. Med..

[1067] John K., Keshava C., Richardson D.L., Weston A., Nath J. (2008). Transcriptional profiles of benzo(a)pyrene exposure in normal human mammary epithelial cells in the absence or presence of chlorophyllin. Mutat. Res..

[1068] Pérez-González A., Castañeda-Arriaga R., Álvarez-Idaboy J.R., Reiter R.J., Galano A. (2018). Melatonin and its metabolites as chemical agents capable of directly repairing oxidized DNA. J. Pineal Res..

[1069] Miyata R., Tanuma N., Sakuma H., Hayashi M. (2016). Circadian Rhythms of Oxidative Stress Markers and Melatonin Metabolite in Patients with Xeroderma Pigmentosum Group A. Oxidative Med. Cell. Longev..

[1070] Rezaeejam H., Shirazi A., Izadi P., Bazzaz J.T., Ghazi-Khansari M., Valizadeh M., Tabesh G.A. (2018). Radioprotective effect of melatonin on expression of and genes in rat peripheral blood. J. Cancer Res. Ther..

[1071] Rezapoor S., Shirazi A., Abbasi S., Bazzaz J.T., Izadi P., Rezaeejam H., Valizadeh M., Soleimani-Mohammadi F., Najafi M. (2017). Modulation of Radiation-induced Base Excision Repair Pathway Gene Expression by Melatonin. J. Med. Phys..

[1072] Valizadeh M., Shirazi A., Izadi P., Bazzaz J.T., Rezaeejam H. (2017). Expression Levels of Two DNA Repair-related Genes under 8 Gy Ionizing Radiation and 100 Mg/Kg Melatonin Delivery In Rat Peripheral Blood. J. Biomed. Phys. Eng..

[1073] Khan S., Adhikari J.S., Rizvi M.A., Chaudhury N.K. (2015). Radioprotective potential of melatonin against 60Co γ-ray-induced testicular injury in male C57BL/6 mice. J. Biomed. Sci..

[1074] Leem J., Bai G.Y., Kim J.S., Oh J.S. (2019). Melatonin protects mouse oocytes from DNA damage by enhancing nonhomologous end-joining repair. J. Pineal Res..

[1075] Schulten H.-J., Bakhashab S. (2019). Meta-Analysis of Microarray Expression Studies on Metformin in Cancer Cell Lines. Int. J. Mol. Sci..

[1076] Kulkarni A.S., Brutsaert E.F., Anghel V., Zhang K., Bloomgarden N., Pollak M., Mar J.C., Hawkins M., Crandall J.P., Barzilai N. (2018). Metformin regulates metabolic and nonmetabolic pathways in skeletal muscle and subcutaneous adipose tissues of older adults. Aging Cell.

[1077] Lee Y.-S., Doonan B.B., Wu J.M., Hsieh T.-C. (2016). Combined metformin and resveratrol confers protection against UVC-induced DNA damage in A549 lung cancer cells via modulation of cell cycle checkpoints and DNA repair. Oncol. Rep..

[1078] Feng T., Li L., Ling S., Fan N., Fang M., Zhang H., Fang X., Lan W., Hou Z., Meng Q. (2015). Metformin enhances radiation response of ECa109 cells through activation of ATM and AMPK. Biomed. Pharm..

[1079] da Silva B.S., Rovaris D.L., Bonotto R.M., Meyer J.B., Grohe R.E., Perassolo M.S., Palazzo R., Maluf S.W., Linden R., de Andrade F.M. (2013). The influence on DNA damage of glycaemic parameters, oral antidiabetic drugs and polymorphisms of genes involved in the DNA repair system. Mutagenesis.

[1080] Wu C.L., Qiang L., Han W., Ming M., Viollet B., He Y.Y. (2013). Role of AMPK in UVB-induced DNA damage repair and growth control. Oncogene.

[1081] Zhou K., Bellenguez C., Spencer C.C., Bennett A.J., Coleman R.L., Tavendale R., Hawley S.A., Donnelly L.A., Schofield C., Groves C.J. (2011). Common variants near ATM are associated with glycemic response to metformin in type 2 diabetes. Nat. Genet..

[1082] Habib S.L., Kasinath B.S., Arya R.R., Vexler S., Velagapudi C. (2010). Novel mechanism of reducing tumourigenesis: Upregulation of the DNA repair enzyme OGG1 by rapamycin-mediated AMPK activation and mTOR inhibition. Eur. J. Cancer.

[1083] Wang X., Shojaie A., Zhang Y., Shelley D., Lampe P.D., Levy L., Peters U., Potter J.D., White E., Lampe J.W. (2017). Exploratory plasma proteomic analysis in a randomized crossover trial of aspirin among healthy men and women. PLoS ONE.

[1084] Dibra H.K., Brown J.E., Hooley P., Nicholl I.D. (2010). Aspirin and alterations in DNA repair proteins in the SW480 colorectal cancer cell line. Oncol. Rep..

[1085] Ramchander N.C., Ryan N.A.J., Crosbie E.J., Evans D.G. (2017). Homozygous germ-line mutation of the PMS2 mismatch repair gene: A unique case report of constitutional mismatch repair deficiency (CMMRD). BMC Med. Genet..

[1086] Chiyoda T., Hart P.C., Eckert M.A., McGregor S.M., Lastra R.R., Hamamoto R., Nakamura Y., Yamada S.D., Olopade O.I., Lengyel E. (2017). Loss of BRCA1 in the Cells of Origin of Ovarian Cancer Induces Glycolysis: A Window of Opportunity for Ovarian Cancer Chemoprevention. Cancer Prev. Res..

[1087] McIlhatton M.A., Tyler J., Kerepesi L.A., Bocker-Edmonston T., Kucherlapati M.H., Edelmann W., Kucherlapati R., Kopelovich L., Fishel R. (2011). Aspirin and low-dose nitric oxide-donating aspirin increase life span in a Lynch syndrome mouse model. Cancer Prev. Res..

[1088] Georgiadis M.M., Chen Q., Meng J., Guo C., Wireman R., Reed A., Vasko M.R., Kelley M.R. (2016). Small molecule activation of apurinic/apyrimidinic endonuclease 1 reduces DNA damage induced by cisplatin in cultured sensory neurons. DNA Repair.

[1089] Zhang B., Niu H., Cai Q., Liao M., Chen K., Chen Y., Cong P. (2019). Roscovitine and Trichostatin A promote DNA damage repair during porcine oocyte maturation. Reprod. Fertil. Dev..

[1090] Tan X., Yang Z., Yang L., Miao X. (2014). Expression of DNA-repair proteins and their significance in pancreatic cancer and non-cancerous pancreatic tissues of Sprague-Dawley rats. World J. Surg. Oncol..

[1091] Bouquet F., Ousset M., Biard D., Fallone F., Dauvillier S., Frit P., Salles B., Muller C. (2011). A DNA-dependent stress response involving DNA-PK occurs in hypoxic cells and contributes to cellular adaptation to hypoxia. J. Cell Sci..

[1092] Smith S., Fox J., Mejia M., Ruangpradit W., Saberi A., Kim S., Choi Y., Oh S., Wang Y., Choi K. (2014). Histone deacetylase inhibitors selectively target homology dependent DNA repair defective cells and elevate non-homologous endjoining activity. PLoS ONE.

[1093] Di Bernardo G., Alessio N., Dell’Aversana C., Casale F., Teti D., Cipollaro M., Altucci L., Galderisi U. (2010). Impact of histone deacetylase inhibitors SAHA and MS-275 on DNA repair pathways in human mesenchymal stem cells. J. Cell. Physiol..

[1094] Majora M., Sondenheimer K., Knechten M., Uthe I., Esser C., Schiavi A., Ventura N., Krutmann J. (2018). HDAC inhibition improves autophagic and lysosomal function to prevent loss of subcutaneous fat in a mouse model of Cockayne syndrome. Sci. Transl. Med..

[1095] Golla U., Joseph D., Tomar R.S. (2016). Combined Transcriptomics and Chemical-Genetics Reveal Molecular Mode of Action of Valproic acid, an Anticancer Molecule using Budding Yeast Model. Sci. Rep..

[1096] Constantinescu D., Csoka A.B., Navara C.S., Schatten G.P. (2010). Defective DSB repair correlates with abnormal nuclear morphology and is improved with FTI treatment in Hutchinson-Gilford progeria syndrome fibroblasts. Exp. Cell Res..

[1097] Zeisel S.H. (2012). Dietary choline deficiency causes DNA strand breaks and alters epigenetic marks on DNA and histones. Mutat. Res..

[1098] Pogribny I.P., James S.J., Beland F.A. (2012). Molecular alterations in hepatocarcinogenesis induced by dietary methyl deficiency. Mol. Nutr. Food Res..

[1099] Rani V., Deep G., Singh R.K., Palle K., Yadav U.C.S. (2016). Oxidative stress and metabolic disorders: Pathogenesis and therapeutic strategies. Life Sci..

[1100] Liakos A., Lavigne M.D., Fousteri M. (2017). Nucleotide Excision Repair: From Neurodegeneration to Cancer. Adv. Exp. Med. Biol..

[1101] Williams D.T., Staples C.J. (2017). Approaches for Identifying Novel Targets in Precision Medicine: Lessons from DNA Repair. Adv. Exp. Med. Biol..

[1102] Singh A., Kukreti R., Saso L., Kukreti S. (2019). Oxidative Stress: A Key Modulator in Neurodegenerative Diseases. Molecules.

[1103] Maiuri T., Suart C.E., Hung C.L.K., Graham K.J., Bazan C.A.B., Truant R. (2019). DNA Damage Repair in Huntington’s Disease and Other Neurodegenerative Diseases. Neurotherapeutics.

[1104] Shaposhnikov M., Proshkina E., Shilova L., Zhavoronkov A., Moskalev A. (2015). Lifespan and Stress Resistance in Drosophila with Overexpressed DNA Repair Genes. Sci. Rep..

[1105] van Meter M., Mao Z., Gorbunova V., Selua A. (2011). SIRT6 overexpression induces massive apoptosis in cancer cells but not in normal cells. Cell Cycle.

[1106] Myrianthopoulos V., Evangelou K., Vasileiou P., Cooks T., Vassilakopoulos T.P., Pangalis G.A., Kouloukoussa M., Kittas C., Georgakilas A.G., Gorgoulis V.G. (2019). Senescence and senotherapeutics: A new field in cancer therapy. Pharmacol. Ther..

[1107] Kim E.-C., Kim J.-R. (2019). Senotherapeutics: Emerging strategy for healthy aging and age-related disease. BMB Rep..

[1108] Thoppil H., Riabowol K. (2020). Senolytics: A Translational Bridge between Cellular Senescence and Organismal Aging. Front. Cell Dev. Boil..

[1109] Zhu Y., Doornebal E.J., Pirtskhalava T., Giorgadze N., Wentworth M., Fuhrmann-Stroissnigg H., Niedernhofer L.J., Robbins P.D., Tchkonia T., Kirkland J.L. (2017). New agents that target senescent cells: The flavone, fisetin, and the BCL-X inhibitors, A1331852 and A1155463. Aging.

[1110] Li W., He Y., Zhang R., Zheng G., Zhou D. (2019). The curcumin analog EF24 is a novel senolytic agent. Aging.

[1111] Lim H., Park H., Kim H.P. (2015). Effects of flavonoids on senescence-associated secretory phenotype formation from bleomycin-induced senescence in BJ fibroblasts. Biochem. Pharmacol..

[1112] Wang Y., Chang J., Liu X., Zhang X., Zhang S., Zhang X., Zhou D., Zheng G. (2016). Discovery of piperlongumine as a potential novel lead for the development of senolytic agents. Aging.

[1113] Guerrero A., Herranz N., Sun B., Wagner V., Gallage S., Guiho R., Wolter K., Pombo J., Irvine E.E., Innes A.J. (2019). Cardiac glycosides are broad-spectrum senolytics. Nat. Metab..

[1114] Triana-Martínez F., Picallos-Rabina P., Da Silva-Álvarez S., Pietrocola F., Llanos S., Rodilla V., Soprano E., Pedrosa P., Ferreirós A., Barradas M. (2019). Identification and characterization of Cardiac Glycosides as senolytic compounds. Nat. Commun..

[1115] Ozsvari B., Nuttall J.R., Sotgia F., Lisanti M.P. (2018). Azithromycin and Roxithromycin define a new family of ‘senolytic’ drugs that target senescent human fibroblasts. Aging.

[1116] Nogueira-Recalde U., Lorenzo-Gómez I., Blanco F.J., Loza M.I., Grassi D., Shirinsky V., Shirinsky I., Lotz M., Robbins P.D., Domínguez E. (2019). Fibrates as drugs with senolytic and autophagic activity for osteoarthritis therapy. EBioMedicine.

[1117] Samaraweera L., Adomako A., Rodriguez-Gabin A., McDaid H.M. (2017). A Novel Indication for Panobinostat as a Senolytic Drug in NSCLC and HNSCC. Sci. Rep..

[1118] Yosef R., Pilpel N., Tokarsky-Amiel R., Biran A., Ovadya Y., Cohen S., Vadai E., Dassa L., Shahar E., Condiotti R. (2016). Directed elimination of senescent cells by inhibition of BCL-W and BCL-XL. Nat. Commun..

[1119] Baar M.P., Brandt R., Putavet D.A., Klein J., Derks K., Bourgeois B., Stryeck S., Rijksen Y., van Willigenburg H., Feijtel D.A. (2017). Targeted Apoptosis of Senescent Cells Restores Tissue Homeostasis in Response to Chemotoxicity and Aging. Cell.

[1120] Zhu Y., Tchkonia T., Fuhrmann-Stroissnigg H., Dai H.M., Ling Y.Y., Stout M.B., Pirtskhalava T., Giorgadze N., Johnson K.O., Giles C.B. (2017). Identification of a novel senolytic agent, navitoclax, targeting the Bcl-2 family of anti-apoptotic factors. Aging Cell.

[1121] Fuhrmann-Stroissnigg H., Ling Y.Y., Zhao J., McGowan S.J., Zhu Y., Brooks R.W., Grassi D., Gregg S.Q., Stripay J.L., Dorronsoro A. (2017). Identification of HSP90 inhibitors as a novel class of senolytics. Nat. Commun..

[1122] Wang R., Yu Z., Sunchu B., Shoaf J., Dang I., Zhao S., Caples K., Bradley L., Beaver L.M., Ho E. (2017). Rapamycin inhibits the secretory phenotype of senescent cells by a Nrf2-independent mechanism. Aging Cell.

[1123] Xu M., Tchkonia T., Ding H., Ogrodnik M., Lubbers E.R., Pirtskhalava T., White T.A., Johnson K.O., Stout M.B., Mezera V. (2015). JAK inhibition alleviates the cellular senescence-associated secretory phenotype and frailty in old age. Proc. Natl. Acad. Sci. USA.

[1124] Vizioli M.G., Liu T., Miller K.N., Robertson N.A., Gilroy K., Lagnado A.B., Perez-Garcia A., Kiourtis C., Dasgupta N., Lei X. (2020). Mitochondria-to-nucleus retrograde signaling drives formation of cytoplasmic chromatin and inflammation in senescence. Genes Dev..

[1125] Wiley C.D., Schaum N., Alimirah F., Lopez-Dominguez J.A., Orjalo A.V., Scott G., Desprez P.Y., Benz C., Davalos A.R., Campisi J. (2018). Small-molecule MDM2 antagonists attenuate the senescence-associated secretory phenotype. Sci. Rep..

[1126] Freund A., Patil C.K., Campisi J. (2011). p38MAPK is a novel DNA damage response-independent regulator of the senescence-associated secretory phenotype. EMBO J..

[1127] Alimbetov D., Davis T., Brook A.J., Cox L.S., Faragher R.G., Nurgozhin T., Zhumadilov Z., Kipling D. (2016). Suppression of the senescence-associated secretory phenotype (SASP) in human fibroblasts using small molecule inhibitors of p38 MAP kinase and MK2. Biogerontology.

[1128] Tasdemir N., Banito A., Roe J.S., Alonso-Curbelo D., Camiolo M., Tschaharganeh D.F., Huang C.H., Aksoy O., Bolden J.E., Chen C.C. (2016). BRD4 Connects Enhancer Remodeling to Senescence Immune Surveillance. Cancer Discov..

[1129] Yousefzadeh M.J., Zhu Y., McGowan S.J., Angelini L., Fuhrmann-Stroissnigg H., Xu M., Ling Y.Y., Melos K.I., Pirtskhalava T., Inman C.L. (2018). Fisetin is a senotherapeutic that extends health and lifespan. EBioMedicine.

[1130] Chang J., Wang Y., Shao L., Laberge R.M., Demaria M., Campisi J., Janakiraman K., Sharpless N.E., Ding S., Feng W. (2015). Clearance of senescent cells by ABT263 rejuvenates aged hematopoietic stem cells in mice. Nat. Med..

[1131] Muñoz-Espín D., Rovira M., Galiana I., Giménez C., Lozano-Torres B., Paez-Ribes M., Llanos S., Chaib S., Muñoz-Martín M., Ucero A.C. (2018). A versatile drug delivery system targeting senescent cells. EMBO Mol. Med..

[1132] Guerrero A., Guiho R., Herranz N., Uren A., Withers D.J., Martínez-Barbera J.P., Tietze L.F., Gil J. (2020). Galactose-modified duocarmycin prodrugs as senolytics. Aging Cell.

[1133] Kim K.M., Noh J.H., Bodogai M., Martindale J.L., Yang X., Indig F.E., Basu S.K., Ohnuma K., Morimoto C., Johnson P.F. (2017). Identification of senescent cell surface targetable protein DPP4. Genes Dev..

[1134] Thapa R.K., Nguyen H.T., Jeong J.H., Kim J.R., Choi H.G., Yong C.S., Kim J.O. (2017). Progressive slowdown/prevention of cellular senescence by CD9-targeted delivery of rapamycin using lactose-wrapped calcium carbonate nanoparticles. Sci. Rep..

[1135] Kang C., Xu Q., Martin T.D., Li M.Z., Demaria M., Aron L., Lu T., Yankner B.A., Campisi J., Elledge S.J. (2015). The DNA damage response induces inflammation and senescence by inhibiting autophagy of GATA4. Science.

[1136] Salminen A., Kauppinen A., Kaarniranta K. (2012). Emerging role of NF-κB signaling in the induction of senescence-associated secretory phenotype (SASP). Cell. Signal..

[1137] Loo T.M., Miyata K., Tanaka Y., Takahashi A. (2020). Cellular senescence and senescence-associated secretory phenotype via the cGAS-STING signaling pathway in cancer. Cancer Sci..

[1138] Maher P. (2015). Fisetin Acts on Multiple Pathways to Reduce the Impact of Age and Disease on CNS Function. Front. Biosci..

[1139] Griveau A., Wiel C., Ziegler D.V., Bergo M.O., Bernard D. (2020). The JAK1/2 inhibitor ruxolitinib delays premature aging phenotypes. Aging Cell.

[1140] Ito T. (2008). Roxithromycin Antagonizes Catagen Induction in Murine and Human Hair Follicles: Implication of Topical Roxithromycin as Hair Restoration Reagent. Arch. Dermatol. Res..

